# Genetic regulation of body size and morphology from adolescence to early adulthood

**DOI:** 10.1038/s41390-025-04259-8

**Published:** 2025-07-14

**Authors:** Karri Silventoinen, Robert F. Krueger, Aline Jelenkovic, Reijo Sund, Glenn I. Roisman, Jaakko Kaprio, Matt McGue

**Affiliations:** 1 Helsinki Institute for Demography and Population Health, University of Helsinki, Helsinki, Finland; 2 Department of Psychology, University of Minnesota Twin Cities, Minneapolis, MI USA; 3 Department of Genetics, Physical Anthropology and Animal Physiology, Faculty of Science and Technology, University of the Basque Country (UPV/EHU), Bilbao, Spain; 4 Institute of Clinical Medicine, University of Eastern Finland, Kuopio, Finland; 5 Institute of Child Development, University of Minnesota Twin Cities, Minneapolis, MI USA; 6 Institute for Molecular Medicine Finland (FIMM), HiLIFE, University of Helsinki, Helsinki, Finland

## Abstract

**Background:**

We analyzed the shared genetic background of extensive anthropometric measurements, determining body size and morphology.

**Methods:**

Anthropometric measurements were taken for 15 traits from 1512 US twins at an average age of 11.7 years (Minnesota, 51% females) and for 20 traits at an average age of 14.8 years for males (*N* = 624) and 18.1 years for females (*N* = 505). Genetic twin modeling was utilized to estimate the genetic correlations between these traits.

**Results:**

In mid to late adolescence, high genetic correlations were found within height-related traits and foot length (r_A_ = 0.58–1.00) as well as within adiposity-related traits (r_A_ = 0.70–0.96), except for skinfold thicknesses. Genetic correlations for craniofacial measurements were smaller (r_A_=0.26–0.80). However, almost all traits showed some genetic correlations with other traits, most of which were at least moderate (r_A_ > 0.30). Results from earlier assessments in early adolescence with fewer traits but a larger sample size were largely similar. Genetic correlations between the initial and follow-up assessments were high (r_A_ = 0.68–0.95), except for craniofacial traits, which showed somewhat lower correlations (r_A_ = 0.40–0.87).

**Conclusions:**

Shared genetic variation plays a significant role in human body size and morphology as well as their development during adolescence.

**Impact:**

- There are clusters of anthropometric traits showing high genetic correlations.
- The highest genetic correlations were found within height- and adiposity-related traits.
- Nearly all anthropometric traits share some genetic variation.
- Genetic factors importantly contribute to the growth of all traits during adolescence.
- Pleiotropic effects are important for understanding the genetic regulation of human physique.

## Introduction

Anthropometric measurements are an essential part of child health check-ups, as abnormal values can signal problems ranging from inadequate nutrition^1^ to serious health conditions.^2^ Understanding the genetic regulation of growth is important for identifying environmental and medical issues that may hinder healthy development. Extensive twin and genome-wide association (GWA) studies have revealed significant associations of genetic factors with height^3,4^ and BMI variation.^5,6^ Additionally, genetic influences have also been shown for other anthropometric traits, such as waist^7^ and chest circumference,^8^ skinfold thicknesses,^9^ and craniofacial measurements.^10^ The same genetic factors largely influence height and BMI throughout childhood and adolescence,^11^ and the role of genetic factors has also been demonstrated for the development of head^12^ and chest circumference.^8^ While previous studies have highlighted the importance of genetic factors in growth, most have focused on height and BMI. Evidence for many anthropometric traits is still lacking, particularly in terms of longitudinal change.

Despite extensive genetic research on anthropometric traits, less is still known about their shared genetic background. Early studies have identified correlation patterns among various anthropometric traits,^13^ suggesting partially correlated genetic components that affect different body parts. Later twin studies have found genetic correlations between BMI and waist circumference^7^ as well as skinfold thicknesses.^14,15^ Some genetically informative studies have also explored genetic correlations among a larger number of anthropometric traits. Studies using two-generation family data from four populations (one from India and three from Europe) found moderate genetic correlations between craniofacial measures^16,17^ and high genetic correlations between adiposity-related measures.^18^ Additionally, a study of Portuguese twins revealed very high genetic correlations between adiposity-related measures.^19^ However, these studies also found weaker yet still significant genetic correlations of adiposity-related measures with height^18,19^ and craniofacial traits.^16^ Collectively, these studies suggest that while there are clusters of anthropometric traits with high genetic correlations, nearly all body parts share some genetic variation.

A limitation in previous studies on genetic correlations among anthropometric measures is that age variation may have affected results, as partly different genetic factors affect human body parts at different ages.^11^ Furthermore, these studies lack longitudinal measures that would allow studying the role of genetic factors in growth. In this study, we will present comprehensive analyses of the genetic background of human body size and morphology assessed using 20 anthropometric measures, using the twin design. Additionally, we will analyze how genetic factors contribute to the growth of 15 of these traits.

## Data and methods

The study cohort was derived from the Minnesota Twin Family Study (MTFS) as described previously.^20^ In summary, the cohort consisted of same-sex twins born in the USA, Minnesota, between 1972 and 1984, identified through Minnesota state records. Contact information was available for 1695 male and 1729 female twins, and around 80% of these twins completed an in-person intake assessment. The twins represented the Minnesota population at the time, with 95% of them being non-Hispanic white and the majority having Northern European ancestry.

The initial anthropometric assessment was conducted when the children had a mean age of 11.7 years (ranging from 10.7 to 12.8 years) and included 1512 twin children (51% girls), comprising 252 male MZ, 233 female MZ, 124 male DZ, and 147 female DZ complete twin pairs. Measurements of 15 anthropometric traits were conducted following a specific measurement protocol (Supplementary Table S1): four measurements of body height (height, sitting height, knee height, and buttock-knee height), the length of both feet, five measures of the head (head circumference, head breadth, face height, and two measures of head length), weight, arm circumference, and waist circumference both relaxed and sucking. Additionally, we calculated body mass index (BMI) by dividing weight in kilograms by the square of height in meters (kg/m^2^). Due to the skewed distributions of weight, BMI, and waist circumference, we used the logarithmic transformation to normalize them. Furthermore, we adjusted the traits for the exact age separately for boys and girls, as the age effect was statistically significant for nearly all measures (Supplementary Table S2).

The follow-up assessment was conducted for 624 males with an average age of 14.8 years old (ranging from 13.6 to 16.9 years) and for 508 females with an average age of 18.1 years old (ranging from 16.6 to 20.3 years), including 207 male MZ, 161 female MZ, 104 male DZ and 93 female DZ complete pairs. The follow-up assessment included all measures from the initial assessment, as well as three breadth measurements (shoulder, wrist, and hip) and two skinfold measures (biceps and triceps) (Supplementary Table S1). Weight, BMI, waist circumference, and skinfold measurements were normalized using logarithmic transformation due to skewed distributions. Nearly all anthropometric traits showed a strong age effect in males but not in females because of their older age at the time of follow-up assessment (Supplementary Table S2). However, to ensure systematic results, we adjusted all traits for age. The age adjustments were made by calculating regression residuals using exact age as an independent variable separately in the initial and follow-up assessments and in males and females. Additionally, we conducted a factor analysis separately for both assessments and sexes using the principal factor estimator and Varimax rotation to analyze the underlying correlation structure. These analyses and all descriptive results were performed using Stata/MP 18.0 for Windows.

Twin modeling was used to analyze the role of genetic factors in the variation and co-variation of anthropometric traits.^21^ This technique is based on the principle that while MZ twins are virtually genetically identical at the gene sequence level, DZ twins share, on average, half of their segregating genes, like ordinary siblings. Since the correlation structure between co-twins is known, it is possible to decompose trait variance into genetic and environmental components. Additive genetic variance (A; correlation 1 within MZ and 0.5 within DZ pairs) includes the effects of all loci influencing the trait. Shared environmental variance (C; correlation 1 within both MZ and DZ pairs) encompasses the effects of all environmental factors that make co-twins similar. Unique environmental variation (E; correlation 0 within both MZ and DZ twins) includes the effects of all environmental factors that make co-twins dissimilar, including measurement error.

Based on co-twin correlations (Supplementary Table S3), we selected the additive genetic/shared environment/unique environment (ACE) model as our baseline model. Model fit statistics are presented in Supplementary Table S4. To test the assumptions of twin modeling, we compared the ACE model to the saturated model, which estimates all possible mean, variance, and covariance statistics without assumptions. The ACE models showed satisfactory fit: 11 traits had poorer fit compared to the saturated model at a conventional p-value of 0.05 and four traits (head breadth, face height, and BMI in the initial assessment and head breadth in the follow-up assessments) at a Bonferroni corrected significance level (*p* < 0.001 using 37 tests). Shared environmental factors were statistically significant for 14 traits at a *p*-value of 0.05, and for four traits at the Bonferroni corrected significance level (*p* < 0.001). Given that most traits exhibited sex differences, we stratified the models by sex.

Using univariate models, we initially calculated the proportions of variation explained by additive genetic factors (a^2^) – i.e., (narrow sense) heritability estimates – as well as shared environmental (c^2^) and unique environmental factors (e^2^). Subsequently, we utilized Cholesky decomposition, a model-free method to decompose all variation and covariation in the data into uncorrelated latent factors.^22^ This method was used to decompose the covariation between the anthropometric measures into genetic and environmental covariances. Standardizing these covariances provides estimates of additive genetic and unique environmental correlations. Since all traits showed some evidence of shared environmental variance, we utilized the ACE model even when shared environmental components were not statistically significant. However, in bivariate models, we initially estimated the additive genetic correlations using the AE model and then confirmed them using the ACE model. This approach was used because the shared environmental correlations could not be accurately calculated due to the small size of shared environmental variation. The genetic twin modeling was conducted using the OpenMx package, version 3.0.2, of R statistical software. The parameters were estimated using the linear structural equations methodology and utilizing the maximum likelihood estimator.^23^

## Results

Table 1 presents the descriptive statistics for the anthropometric traits in the initial and follow-up assessments. Growth was observed in all traits between the assessments. The relative growth was lowest for the head-related traits (2–3%) and highest for the adiposity-related traits (weight, BMI, arm circumference, and waist circumference; 15–49%). In the initial assessment, the measures were roughly similar in boys and girls. In the follow-up assessments, the values are not comparable due to the age difference.

**Table 1:** Descriptive statistics of age and anthropometric traits in the initial and follow-up assessments by sex.

	Initial assessment				Follow-up assessment			
	Male		Female		Male		Female	
	mean	SD	mean	SD	mean	SD	mean	SD
Age years	11.7	0.39	11.7	0.46	14.8	0.49	18.3	0.71
Height cm	149	7.02	151	7.31	170	7.85	166	6.4
Sitting height cm	77.1	3.48	78.2	4.12	86.8	4.47	87.1	3.25
Knee height cm	46.7	2.84	46.8	2.77	52.9	2.85	50.5	2.63
Buttock-knee length cm	51.7	3.23	52.7	3.44	59.1	3.34	58.4	2.94
Foot length left cm	23.0	1.42	22.5	1.31	25.7	1.38	23.5	1.25
Foot length right cm	23.1	1.38	22.5	1.32	25.7	1.37	23.6	1.25
Head circumference cm	54.1	1.62	54.1	1.75	55.9	1.68	55.5	1.75
Head breadth cm	14.4	0.48	14.3	0.50	14.9	0.53	14.6	0.52
Face height cm	20.7	1.09	20.0	1.12	21.8	1.07	20.8	1.03
Head length 1 cm	18.6	0.70	18.3	0.81	19.0	0.84	18.8	0.82
Head length 2 cm	16.8	0.86	16.4	0.95	17.3	0.95	17.2	0.84
Weight kg	41.3	9.53	44.5	11.26	61.7	13.03	65.2	14.73
BMI kg/m^2^	18.4	3.22	19.4	3.73	21.2	3.58	23.7	4.91
Arm circumference cm	21.8	2.88	22.6	3.24	25.9	3.29	26.6	3.67
Waist circ. relaxed cm	65.7	9.00	67.2	9.74	76.2	9.17	78.5	11.28
Waist circ. sucking cm	60.6	8.87	62.6	9.90	71.1	9.69	73.2	11.31
Shoulder breadth cm	NA	NA	NA	NA	33.8	3.07	34.2	2.88
Wrist breadth cm	NA	NA	NA	NA	5.56	0.33	5.18	0.33
Hip breadth cm	NA	NA	NA	NA	30.4	3.03	34.3	3.17
Biceps skinfold mm	NA	NA	NA	NA	8.19	5.93	11.73	6.71
Triceps skinfold mm	NA	NA	NA	NA	13.3	8.30	20.1	7.89

Table 2 presents the relative variance components of genetic and environmental factors. Additive genetic factors explained a significant proportion of the variation in all anthropometric traits, with heritability estimates generally lower for craniofacial traits (a^2^ ranged between 0.21 and 0.85) compared to the other traits (a^2^ ranged between 0.23 and 0.90). Shared environmental factors also contributed to the variation in most traits (c^2^ ranged between 0.00 and 0.63), although the shared environmental variances were not statistically significant for many traits. There were no consistent differences in the heritability estimates between males and females. In general, shared environmental factors explained more and additive genetic factors less of the trait variation in the follow-up than in the initial assessment.

**Table 2:** Relative variance components of additive genetic, shared environmental, and unique environmental factors of anthropometric traits by sex.

	Males									Females								
	Additive genetic factors			Shared environmental factors			Unique environmental factors			Additive genetic factors			Shared environmental factors			Unique environmental factors		
	a^2^	95% CI		c^2^	95% CI		e^2^	95% CI		a^2^	95% CI		c^2^	95% CI		e^2^	95% CI	
		LL	UL		LL	UL		LL	UL		LL	UL		LL	UL		LL	UL
Initial assessment																		
Height	0.78	0.57	0.94	0.15	0.00	0.36	0.07	0.06	0.09	0.71	0.54	0.93	0.22	0.00	0.40	0.07	0.05	0.08
Sitting height	0.75	0.52	0.88	0.11	0.00	0.34	0.14	0.11	0.17	0.67	0.49	0.90	0.23	0.00	0.42	0.10	0.08	0.12
Knee height	0.81	0.60	0.94	0.11	0.00	0.32	0.07	0.06	0.09	0.73	0.53	0.91	0.17	0.00	0.37	0.10	0.08	0.12
Buttock-knee length	0.39	0.22	0.61	0.44	0.22	0.60	0.17	0.14	0.21	0.73	0.53	0.89	0.14	0.00	0.34	0.13	0.10	0.16
Foot length left	0.82	0.60	0.92	0.08	0.00	0.30	0.09	0.08	0.12	0.66	0.47	0.89	0.23	0.00	0.42	0.11	0.09	0.14
Foot length right	0.83	0.61	0.93	0.09	0.00	0.31	0.08	0.07	0.10	0.64	0.45	0.88	0.23	0.00	0.42	0.13	0.10	0.16
Head circ	0.49	0.30	0.74	0.35	0.09	0.53	0.17	0.14	0.21	0.84	0.61	0.88	0.01	0.00	0.24	0.15	0.12	0.18
Head breadth	0.73	0.49	0.84	0.08	0.00	0.31	0.20	0.16	0.24	0.73	0.53	0.89	0.14	0.00	0.33	0.13	0.11	0.17
Head length 1	0.58	0.34	0.80	0.19	0.00	0.41	0.24	0.19	0.29	0.67	0.43	0.81	0.11	0.00	0.34	0.23	0.18	0.28
Head length 2	0.53	0.31	0.81	0.26	0.00	0.48	0.21	0.17	0.25	0.48	0.29	0.71	0.34	0.12	0.52	0.18	0.15	0.23
Face height	0.30	0.07	0.62	0.37	0.03	0.61	0.34	0.28	0.40	0.41	0.10	0.65	0.17	0.00	0.45	0.42	0.35	0.50
Weight	0.79	0.59	0.92	0.12	0.00	0.32	0.09	0.07	0.11	0.78	0.59	0.93	0.14	0.00	0.33	0.08	0.06	0.10
BMI	0.79	0.59	0.92	0.12	0.00	0.32	0.09	0.07	0.11	0.82	0.63	0.93	0.10	0.00	0.29	0.08	0.07	0.10
Arm circ	0.76	0.55	0.90	0.11	0.00	0.32	0.12	0.10	0.15	0.83	0.62	0.92	0.07	0.00	0.28	0.10	0.08	0.12
Waist circ. relax	0.55	0.36	0.78	0.30	0.07	0.48	0.15	0.12	0.19	0.65	0.45	0.86	0.19	0.00	0.39	0.15	0.12	0.19
Waist circ. sucking	0.68	0.47	0.87	0.17	0.00	0.38	0.15	0.12	0.19	0.68	0.48	0.88	0.18	0.00	0.38	0.14	0.11	0.17
Follow-up assessment																		
Height	0.65	0.44	0.90	0.24	0.00	0.46	0.11	0.09	0.14	0.78	0.54	0.95	0.17	0.00	0.41	0.06	0.04	0.07
Sitting height	0.67	0.44	0.88	0.18	0.00	0.41	0.15	0.12	0.19	0.87	0.83	0.90	0.00	0.00	0.19	0.13	0.10	0.17
Knee height	0.62	0.41	0.88	0.24	0.00	0.45	0.13	0.11	0.17	0.70	0.41	0.85	0.11	0.00	0.39	0.19	0.15	0.25
Buttock-knee length	0.46	0.26	0.71	0.37	0.12	0.56	0.17	0.14	0.22	0.63	0.35	0.84	0.18	0.00	0.45	0.19	0.15	0.25
Foot length left	0.69	0.45	0.86	0.14	0.00	0.38	0.17	0.13	0.21	0.90	0.87	0.92	0.00	0.00	0.19	0.10	0.08	0.13
Foot length right	0.67	0.44	0.87	0.17	0.00	0.40	0.16	0.13	0.20	0.88	0.85	0.91	0.00	0.00	0.27	0.12	0.09	0.15
Head circ	0.57	0.35	0.84	0.25	0.00	0.47	0.18	0.14	0.22	0.85	0.80	0.88	0.00	0.00	0.27	0.15	0.12	0.20
Head breadth	0.67	0.44	0.85	0.15	0.00	0.38	0.18	0.14	0.22	0.84	0.79	0.88	0.00	0.00	0.24	0.16	0.12	0.21
Head length 1	0.43	0.21	0.72	0.35	0.06	0.56	0.22	0.18	0.27	0.44	0.13	0.73	0.23	0.00	0.50	0.33	0.26	0.42
Head length 2	0.37	0.17	0.63	0.43	0.17	0.62	0.20	0.16	0.25	0.54	0.22	0.73	0.12	0.00	0.41	0.34	0.27	0.43
Face height	0.21	0.00	0.53	0.42	0.11	0.64	0.37	0.30	0.46	0.65	0.29	0.73	0.01	0.00	0.35	0.34	0.27	0.43
Shoulder breadth	0.27	0.10	0.48	0.55	0.34	0.70	0.19	0.15	0.24	0.23	0.09	0.41	0.63	0.45	0.77	0.14	0.11	0.18
Wrist breadth	0.64	0.38	0.82	0.14	0.00	0.38	0.22	0.18	0.28	0.61	0.31	0.82	0.17	0.00	0.46	0.22	0.17	0.28
Weight	0.71	0.50	0.91	0.19	0.00	0.40	0.10	0.08	0.12	0.71	0.47	0.91	0.18	0.00	0.42	0.11	0.09	0.15
BMI	0.77	0.55	0.91	0.12	0.00	0.34	0.11	0.09	0.14	0.62	0.40	0.89	0.26	0.00	0.48	0.12	0.09	0.16
Arm circ	0.79	0.56	0.91	0.10	0.00	0.33	0.12	0.09	0.15	0.67	0.39	0.83	0.11	0.00	0.38	0.22	0.17	0.29
Hip breadth	0.59	0.40	0.84	0.31	0.06	0.50	0.11	0.08	0.13	0.48	0.27	0.78	0.36	0.07	0.57	0.16	0.12	0.21
Waist circ. relax	0.61	0.39	0.85	0.22	0.00	0.44	0.18	0.14	0.22	0.83	0.78	0.87	0.00	0.00	0.27	0.17	0.13	0.22
Waist circ. sucking	0.49	0.27	0.77	0.31	0.06	0.52	0.20	0.16	0.26	0.45	0.21	0.77	0.35	0.03	0.58	0.20	0.16	0.26
Biceps skinfold	0.57	0.36	0.84	0.26	0.00	0.46	0.17	0.13	0.21	0.15	0.00	0.41	0.60	0.35	0.76	0.25	0.19	0.32
Triceps skinfold	0.42	0.23	0.67	0.40	0.16	0.58	0.18	0.14	0.22	0.24	0.00	0.54	0.47	0.19	0.68	0.30	0.23	0.38

**Fig. 1:**
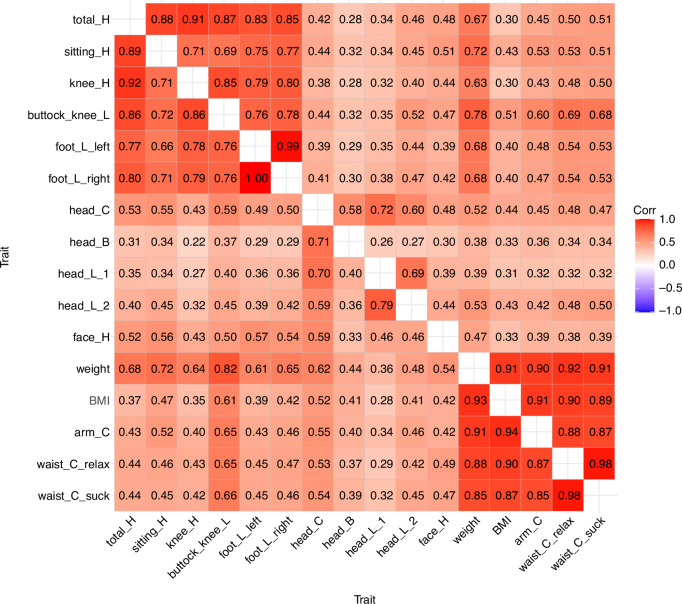
Additive genetic correlations of anthropometric traits in males (upper diagonal matrix) and females (lower diagonal matrix) in the initial assessment. B breadth, C circumference, H height, L length.

After completing the univariate analyses, we proceeded to analyze the mutual correlations between the traits in the initial assessment. Factor analysis revealed three factors in both males and females: the first factor reflected linear traits (height-related traits and foot-length), the second factor reflected volume traits (BMI, arm circumference, and waist circumference), and the third factor reflected craniofacial traits (the factor analyses available in Supplementary Table S5, the trait correlations in Supplementary Fig. S1, and their 95% CIs in Supplementary Table S6). However, all traits showed some loading on all three factors. Next, we decomposed the trait correlations into genetic and unique environmental correlations. Figure 1 presents the additive genetic correlations for boys (upper diagonal matrix) and girls (lower diagonal matrix) (95% CIs are available in Supplementary Table S7). Two clusters of genetic correlations were identified: the components of height and foot length exhibited high genetic correlations (r_A_ = 0.69–1.00), as did the adiposity-related traits (r_A_ = 0.85–0.98). However, most of the genetic correlations were moderate (r_A_≥0.30) indicating that all traits shared some genetic variation. Since these correlations may be influenced by shared environmental factors, we recalculated them allowing shared environmental correlations (Supplementary Table S8). However, the changes were generally modest, and, in some cases, the correlations increased. Shared environmental correlations could not be reliably estimated, and 95% CIs were very broad, ranging for many trait pairs from −1.00 to 1.00 (Supplementary Table S9). We also identified the unique environmental correlations between the traits, but they were lower than the additive genetic correlations (Supplementary Fig. S2; 95% confidence intervals are presented in Supplementary Table S10).

**Fig. 2:**
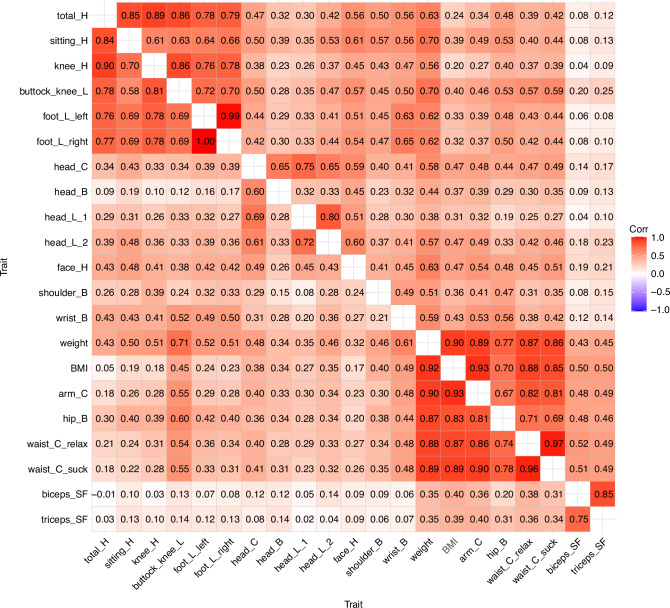
Additive genetic correlations of anthropometric traits in males (upper diagonal matrix) and females (lower diagonal matrix) in the follow-up assessments. B breadth, C circumference, H height, L length, SF skinfold.

We repeated the analyses using the more comprehensive anthropometric measurements available in the follow-up assessment. Generally, the results aligned with those from the initial assessment. In the factor analysis, we identified three factors in both males and females: the first factor reflected volume traits, the second factor reflected linear traits, and the third factor reflected craniofacial traits (the factor analysis is available in Supplementary Table S11, the trait correlation in Supplementary Fig. S3, and their 95% CIs in Supplementary Table S12). Figure 2 presents the additive genetic correlations for boys (upper diagonal matrix) and girls (lower diagonal matrix) (95% CIs are presented in Supplementary Table S13). We observed clusters of genetic correlations within the height-related traits and foot-length (r_A_ = 0.58–1.00) and adiposity-related traits (r_A_ = 0.70–0.96). However, skinfold thicknesses exhibited only moderate genetic correlations with the adiposity-related traits (r_A_ = 0.26–0.50), and the correlations with other traits were close to zero. Craniofacial traits showed somewhat weaker mutual correlations (r_A_ = 0.26–0.80), and they also had moderate genetic correlations (r_A_ ≥ 0.30) with height-related and adiposity-related traits. When we estimated the additive genetic correlations while allowing for shared environmental correlations, the changes were generally modest and in some cases the correlations increased (Supplementary Table S14). In alignment with the initial assessment, the shared environmental correlations could not be reliably estimated (Supplementary Table S15). Unique environmental correlations were lower than additive genetic correlations (Supplementary Fig. S4; 95% confidence intervals are presented in Supplementary Table S16).

Finally, we examined the correlations of anthropometric traits between the initial and follow-up assessments (Table 3). The trait correlations ranged from 0.34 to 0.90. Correlations for face height and length were lower than for other traits, but no systematic differences were observed otherwise. Additive genetic correlations were consistently higher, while unique environmental correlations were lower than the trait correlations.

**Table 3:** Trait correlations and additive genetic and unique environmental correlations between the anthropometric traits in initial and follow-up assessments by sex.

	Trait correlation			Additive genetic correlation			Unique environmental correlation		
	*r*	95% CI		r_A_	95% CI		r_E_	95% CI	
		_LL_	UL		LL	UL		LL	UL
**Males**									
Height	0.87	0.85	0.89	0.90	0.87	0.92	0.56	0.46	0.64
Sitting height	0.79	0.76	0.82	0.85	0.81	0.88	0.41	0.29	0.51
Knee height	0.80	0.77	0.83	0.85	0.82	0.88	0.37	0.26	0.48
Buttock-knee length	0.79	0.76	0.82	0.86	0.82	0.9	0.36	0.24	0.47
Foot length left	0.81	0.78	0.84	0.87	0.83	0.9	0.41	0.29	0.51
Foot length right	0.82	0.79	0.84	0.88	0.85	0.91	0.35	0.23	0.46
Head circumference	0.75	0.71	0.78	0.79	0.74	0.83	0.60	0.51	0.67
Head breadth	0.87	0.85	0.89	0.93	0.91	0.95	0.60	0.52	0.68
Face height	0.48	0.41	0.54	0.64	0.54	0.73	0.12	0.00	0.24
Head length 1	0.65	0.60	0.69	0.75	0.69	0.81	0.30	0.18	0.42
Head length 2	0.45	0.38	0.51	0.49	0.39	0.57	0.27	0.15	0.39
Weight	0.91	0.90	0.92	0.94	0.92	0.95	0.64	0.56	0.71
BMI	0.90	0.89	0.92	0.95	0.93	0.96	0.53	0.43	0.62
Arm circumference	0.82	0.79	0.84	0.86	0.82	0.89	0.52	0.42	0.61
Waist circ. relaxed	0.80	0.77	0.83	0.88	0.85	0.91	0.41	0.30	0.51
Waist circ. sucking	0.75	0.72	0.79	0.84	0.79	0.88	0.37	0.25	0.48
**Females**									
Height	0.71	0.66	0.75	0.73	0.67	0.78	0.49	0.36	0.59
Sitting height	0.68	0.63	0.73	0.73	0.67	0.79	0.29	0.15	0.43
Knee height	0.70	0.65	0.74	0.78	0.72	0.83	0.13	-0.02	0.28
Buttock-knee length	0.66	0.61	0.71	0.72	0.64	0.78	0.34	0.20	0.47
Foot length left	0.82	0.79	0.84	0.83	0.79	0.87	0.62	0.51	0.71
Foot length right	0.81	0.78	0.84	0.85	0.81	0.89	0.46	0.33	0.57
Head circumference	0.72	0.67	0.76	0.77	0.70	0.82	0.40	0.26	0.52
Head breadth	0.83	0.80	0.85	0.87	0.83	0.90	0.61	0.50	0.70
Face height	0.49	0.42	0.56	0.70	0.59	0.81	0.06	-0.09	0.21
Head length 1	0.51	0.44	0.57	0.59	0.48	0.68	0.29	0.15	0.42
Head length 2	0.34	0.26	0.42	0.40	0.27	0.51	0.23	0.08	0.37
Weight	0.78	0.74	0.81	0.80	0.75	0.84	0.52	0.39	0.62
BMI	0.79	0.76	0.82	0.82	0.78	0.86	0.43	0.29	0.54
Arm circumference	0.70	0.65	0.74	0.77	0.70	0.83	0.3	0.15	0.43
Waist circ. relaxed	0.66	0.60	0.70	0.72	0.64	0.78	0.22	0.06	0.36
Waist circ. sucking	0.62	0.56	0.67	0.68	0.59	0.75	0.28	0.14	0.42

## Discussion

In this comprehensive study of human body size and morphology, we observed that genetic factors explained a major proportion of the variation in 21 anthropometric traits. For height and BMI, the heritability estimates were high, showing that between 62% to 82% of the variation is explained by genetic differences. However, shared environmental factors also explained a portion of the variation in nearly all anthropometric traits. There was also some evidence that the proportion of shared environmental variation increased from the initial to the follow-up assessment. This result is different than what was found in large-scale twin studies on height^3^ and BMI,^5^ suggesting that shared environmental variation tends to decrease from childhood to adolescence. However, there is a lack of studies on other anthropometric measures at different ages, and the CIs of shared environmental variance in our study were wide. Differentiating between shared environmental factors and genetic factors requires considerable statistical power,^24^ often leading to the neglect of shared environmental factors in genetic twin studies. These results suggest that shared environmental factors can affect the variation of anthropometric traits along with genetic factors and should be better recognized in future studies. Interestingly, the heritability estimates for craniofacial traits were consistently lower compared to height- and adiposity-related traits, suggesting that environmental factors unique to individuals may play a role in shaping facial features. Previous studies using pedigree data have reported heritability estimates for craniofacial measures at a similar level to those in our study.^16,25^ However, measurement error is also included in unique environmental variation, which can explain the lower heritability of these traits.

For 16 of these 21 traits, we had longitudinal measures that allowed us to examine factors related to growth over adolescence, from about 11 years onward. As expected, there was substantial growth for height- and adiposity-related traits, but minimal growth for craniofacial traits, reflecting the normal physical development of children during adolescence.^26^ Genetic correlations for height- and adiposity-related traits were high, indicating that a large proportion of the genetic factors affecting these traits during puberty are the same. These genetic correlations were very similar to those found for height and BMI during adolescence in a previous large-scale twin study that pooled data from several twin cohorts.^11^ For craniofacial traits, the genetic correlations were lower than for other traits, suggesting that partly different sets of genetic factors begin to shape the head during adolescence. These results indicate that the genetic regulation of different parts of the body can vary over the course of aging. However, it is noteworthy that there can be more measurement error in craniofacial than in other measurements, which can also lead to lower genetic correlations.

We identified three clusters of genetic correlations. Firstly, height-related measures and the length of both feet exhibited high genetic correlations. Large GWA studies^27^ and whole-genome studies^28^ have identified numerous genes associated with height. Genes contributing to skeletal growth and the development of cartilage and connective tissues are enriched in these studies. Building on such genetic findings, studies using immortalized chondrogenic murine cell lines demonstrated that genes associated with height are expressed highly in growth plate chondrocytes.^27,29^ Our findings suggest that partly the same genetic factors regulate the ossification of long bones and feet bones. The second cluster encompassed adiposity-related traits, which have also shown high genetic correlations in previous pedigree^18^ and twin studies.^19^ Evidence from GWA, twin, and epidemiological studies supports the idea that eating behavior plays a significant role in mediating the influences of genetic factors on BMI.^30^ Therefore, nutrition probably influences these genetic correlations. However, unlike previous studies,^18,19^ our study found only moderate genetic correlations between skinfold thicknesses and BMI/waist circumference. A previous GWA study indicated that genetic variants linked to body fat distribution are associated with lipid metabolism and adipose tissue regulation.^31^ It is possible that these genetic effects are more pronounced in adolescence, leading to decreased genetic correlations in our data. The third cluster involved craniofacial traits, but the genetic correlations among them were weaker compared to height- and adiposity-related traits. The genetic complexity of craniofacial traits was also highlighted in a recent GWA study.^32^ These results are expected given the intricate nature of the head, which comprises both soft and bone tissues likely influenced by distinct genetic factors.

In addition to these clusters of genetic correlations, we found that nearly all traits showed some shared genetic variation, as seen in previous studies. The genetic correlations were generally higher than the trait correlations, indicating that mainly genetic factors are behind the anthropometric correlations. These results are not surprising, as pleiotropy is very common in human traits.^33^ However, there can be several mechanisms behind pleiotropic effects,^34^ and we can only speculate on which factors explain them in our study. Since all body parts are formed by the same tissues, the underlying shared genetic mechanisms that regulate the growth and development of bone, adipose, and muscle tissues can explain genetic correlations between even distinct body parts such as craniofacial and adiposity-related traits. These genetic correlations may have a background in embryonic development when different body parts develop from the same embryonic structures.^35^ It is also possible that the postnatal environment, especially nutrition, can affect different traits and explain these genetic correlations.^30^ This is evident for adiposity-related traits but may also explain correlations between height- and adiposity-related traits. Disentangling these different mechanisms requires different study designs, such as combining detailed measures of potential environmental factors with longitudinal anthropometric measures.

Since we found evidence of shared environmental factors, we also studied the shared environmental correlations between the traits. However, we found that the shared environmental variance components were too low to reliably estimate these correlations. Therefore, we cannot determine if there are environmental factors shared by co-twins that affect different anthropometric traits. We calculated all genetic correlations while also accounting for shared environmental influences, but this had a modest effect on the magnitude of genetic correlations, and in some cases, they even increased. This supports the conclusion that the genetic correlations reflect a shared genetic background between the traits rather than unspecified familial effects combining shared genetic and environmental influences.

Our study has strengths and limitations. We have detailed anthropometric measurements in genetically informative data allowing us to analyze the genetics of body morphology in detail. Additionally, we had longitudinal data for several traits, which enabled us to analyze how genetic factors impact growth during adolescence. Our dataset was not large enough to simultaneously estimate genetic and shared environmental effects with sufficient power. However, we confirmed that shared environmental factors had minimal impact on genetic correlations. Our main limitation is the lack of dual-energy X-ray absorptiometry (DEXA) or computer tomography measurements, which would have allowed us to directly measure body composition. Likewise, magnetic resonance imaging (MRI) could be used to assess, for example, the bony structure of the skull. This information would have been valuable in analyzing the extent to which different anthropometric traits share common genetic variance with body composition measures. The population of Minnesota during the data collection period was ethnically homogeneous. It is likely that in more heterogeneous populations, heritability estimates would be lower compared to this study, due to a larger shared environmental variation.

In conclusion, we found that shared genetic factors broadly affect the body size and morphology of humans. Clusters of high genetic correlations were identified for height- and adiposity-related traits, but most of the traits shared some genetic variation. Genetic factors also largely explained the correlations of these traits over adolescence. Pleiotropic effects are important for understanding the genetic regulation of human physique.

## Supplementary information

Supplementary information

## Data Availability

The data are freely available for research purposes. Any inquiries should be sent to Matt McGue (mcgue001@umn.edu).

## References

[1] Norris, S. A. et al. Nutrition in adolescent growth and development. *Lancet* **399**, 172–184 (2022). 34856190 10.1016/S0140-6736(21)01590-7

[2] Harju, S., Saari, A., Sund, R. & Sankilampi, U. Epidemiology of disorders associated with short stature in childhood: a 20-year birth cohort study in Finland. *Clin. Epidemiol.* **14**, 1205–1214 (2022). 36320440 10.2147/CLEP.S372870 PMC9618248

[3] Jelenkovic, A. et al. Genetic and environmental influences on height from infancy to early adulthood: An individual-based pooled analysis of 45 twin cohorts. *Sci. Rep.* **6**, 28496 (2016). 27333805 10.1038/srep28496 PMC4917845

[4] Yengo, L. et al. A saturated map of common genetic variants associated with human height. *Nature* **610**, 704–712 (2022). 36224396 10.1038/s41586-022-05275-y PMC9605867

[5] Silventoinen, K. et al. Genetic and environmental effects on body mass index from infancy to the onset of adulthood: an individual-based pooled analysis of 45 twin cohorts participating in the COllaborative project of Development of Anthropometrical measures in Twins (CODATwins) study. *Am. J. Clin. Nutr.* **104**, 371–379 (2016). 27413137 10.3945/ajcn.116.130252 PMC4962159

[6] Turcot, V. et al. Protein-altering variants associated with body mass index implicate pathways that control energy intake and expenditure in obesity. *Nat. Genet.* **50**, 26–41 (2018). 29273807 10.1038/s41588-017-0011-x PMC5945951

[7] Wardle, J., Carnell, S., Haworth, C. M. & Plomin, R. Evidence for a strong genetic influence on childhood adiposity despite the force of the obesogenic environment. *Am. J. Clin. Nutr.* **87**, 398–404 (2008). 18258631 10.1093/ajcn/87.2.398

[8] Silventoinen, K., Kaprio, J., Dunkel, L. & Yokoyama, Y. Genetic and environmental influences on chest circumference during infancy: a longitudinal study of Japanese twins. *Paediatr. Perinat. Epidemiol.* **26**, 553–560 (2012). 23061691 10.1111/ppe.12003

[9] Peeters, M. W. et al. Genetic and environmental determination of tracking in subcutaneous fat distribution during adolescence. *Am. J. Clin. Nutr.* **86**, 652–660 (2007). 17823430 10.1093/ajcn/86.3.652

[10] Jelenkovic, A., Poveda, A., Susanne, C. & Rebato, E. Contribution of genetics and environment to craniofacial anthropometric phenotypes in Belgian nuclear families. *Hum. Biol.* **80**, 637–654 (2008). 19728541 10.3378/1534-6617-80.6.637

[11] Silventoinen, K. et al. Changing genetic architecture of body mass index from infancy to early adulthood: an individual based pooled analysis of 25 twin cohorts. *Int. J. Obes.* **46**, 1901–1909 (2022). 10.1038/s41366-022-01202-3 PMC9492534 35945263

[12] Silventoinen, K. et al. Genetics of head circumference in infancy: a longitudinal study of Japanese twins. *Am. J. Hum. Biol.* **23**, 630–634 (2011). 21630369 10.1002/ajhb.21190

[13] Devor, E. J., McGue, M., Crawford, M. H. & Lin, P. M. Transmissible and nontransmissible components of anthropometric variation in the Alexanderwohl Mennonites: II. Resolution by path analysis. *Am. J. Phys. Anthropol.* **69**, 83–92 (1986). 3946598 10.1002/ajpa.1330690110

[14] Beunen, G. et al. Univariate and multivariate genetic analysis of subcutaneous fatness and fat distribution in early adolescence. *Behav. Genet.* **28**, 279–288 (1998). 9803020 10.1023/a:1021671313974

[15] Hasselbalch, A. L. et al. Common genetic components of obesity traits and serum leptin. *Obesity* **16**, 2723–2729 (2008). 18927547 10.1038/oby.2008.440

[16] Ghosh, S., Kasher, M., Malkina, I. & Livshits, G. Is craniofacial morphology and body composition related by common genes: Comparative analysis of two ethnically diverse populations. *Am. J. Phys. Anthropol.* **176**, 249–261 (2021). 34297355 10.1002/ajpa.24373

[17] Jelenkovic, A., Poveda, A., Susanne, C. & Rebato, E. Common genetic and environmental factors among craniofacial traits in Belgian nuclear families: comparing skeletal and soft-tissue related phenotypes. *Homo* **61**, 191–203 (2010). 20149367 10.1016/j.jchb.2009.10.003

[18] Jelenkovic, A. & Rebato, E. Association among obesity-related anthropometric phenotypes: analyzing genetic and environmental contribution. *Hum. Biol.* **84**, 127–137 (2012). 22708817 10.3378/027.084.0202

[19] Silventoinen, K. et al. Genetic regulation of body size and morphology in children: a twin study of 22 anthropometric traits. *Int J. Obes.* **47**, 181–289 (2023). 10.1038/s41366-023-01253-0 PMC10023566 36635383

[20] Iacono, W. G. & McGue, M. Minnesota twin family study. *Twin Res.* **5**, 482–487 (2002). 12537881 10.1375/136905202320906327

[21] Posthuma, D. et al. Theory and practice in quantitative genetics. *Twin Res*. **6**, 361–376 (2003). 14624720 10.1375/136905203770326367

[22] Kaprio, J. & Silventoinen, K. Advanced methods in twin studies. *Methods Mol. Biol.* **713**, 143–152 (2011). 21153617 10.1007/978-1-60327-416-6_11

[23] Neale, M. C. et al. OpenMx 2.0: Extended structural equation and statistical modeling. *Psychometrika* **81**, 535–549 (2016). 25622929 10.1007/s11336-014-9435-8 PMC4516707

[24] Visscher, P. M., Gordon, S. & Neale, M. C. Power of the classical twin design revisited: II detection of common environmental variance. *Twin Res. Hum. Genet.* **11**, 48–54 (2008). 18251675 10.1375/twin.11.1.48 PMC3996914

[25] Cole, J. B. et al. Human facial shape and size heritability and genetic correlations. *Genetics* **205**, 967–978 (2017). 27974501 10.1534/genetics.116.193185 PMC5289863

[26] Malina M., Bouchard C., Bar-Or O. Growth, Maturation and Physical Growth. 2. Champaign, IL, USA: Human Kinetics; 2004.

[27] Yengo, L. et al. Meta-analysis of genome-wide association studies for height and body mass index in ∼700000 individuals of European ancestry. *Hum. Mol. Genet.* **27**, 3641–3649 (2018). 30124842 10.1093/hmg/ddy271 PMC6488973

[28] Wainschtein, P. et al. Assessing the contribution of rare variants to complex trait heritability from whole-genome sequence data. *Nat. Genet.* **54**, 263–273 (2022). 35256806 10.1038/s41588-021-00997-7 PMC9119698

[29] Renthal, N. E., Nakka, P., Baronas, J. M., Kronenberg, H. M. & Hirschhorn, J. N. Genes with specificity for expression in the round cell layer of the growth plate are enriched in genomewide association study (GWAS) of human height. *J. Bone Min. Res.* **36**, 2300–2308 (2021). 10.1002/jbmr.4408 PMC10249375 34346115

[30] Silventoinen, K. & Konttinen, H. Obesity and eating behavior from the perspective of twin and genetic research. *Neurosci. Biobehav. Rev.* **109**, 150–165 (2020). 31959301 10.1016/j.neubiorev.2019.12.012

[31] Justice, A. E. et al. Protein-coding variants implicate novel genes related to lipid homeostasis contributing to body-fat distribution. *Nat. Genet.* **51**, 452–469 (2019). 30778226 10.1038/s41588-018-0334-2 PMC6560635

[32] White, J. D. et al. Insights into the genetic architecture of the human face. *Nat. Genet.* **53**, 45–53 (2021). 33288918 10.1038/s41588-020-00741-7 PMC7796995

[33] Watanabe, K. et al. A global overview of pleiotropy and genetic architecture in complex traits. *Nat. Genet.* **51**, 1339–1348 (2019). 31427789 10.1038/s41588-019-0481-0

[34] Paaby, A. B. & Rockman, M. V. The many faces of pleiotropy. *Trends Genet.* **29**, 66–73 (2013). 23140989 10.1016/j.tig.2012.10.010 PMC3558540

[35] Tickle C. How the embryo makes a limb: determination, polarity and identity. *J. Anat.* **227**:418–430 (2015). 10.1111/joa.12361 PMC4580101 26249743

