# Supplementary material for: Genetic regulation of body size and morphology from adolescence to early adulthood
Source: Pediatr Res. 2025 Jul 14;99(3):1164–72. doi: 10.1038/s41390-025-04259-8 (PMC13021503; doi:10.1038/s41390-025-04259-8)
Supplement: Supplementary file 1 — Supplementary information [file 41390_2025_4259_MOESM1_ESM.pdf]

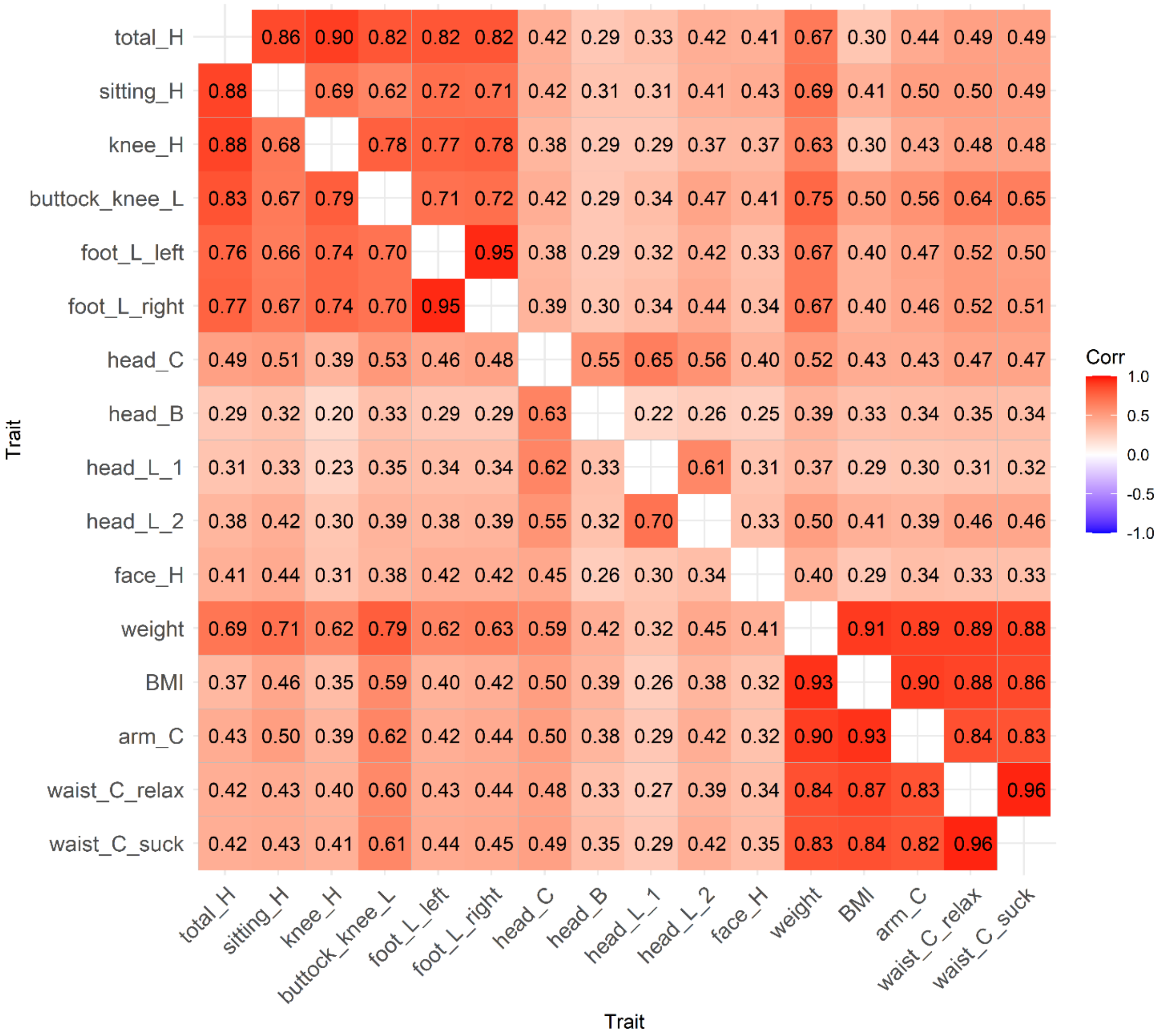


Supplemental Figure S1. Correlations of anthropometric traits in males (upper diagonal matrix) and females (lower diagonal matrix) in the initial assessment. Abbreviations: B, breadth; C, circumference; H, height; L, length

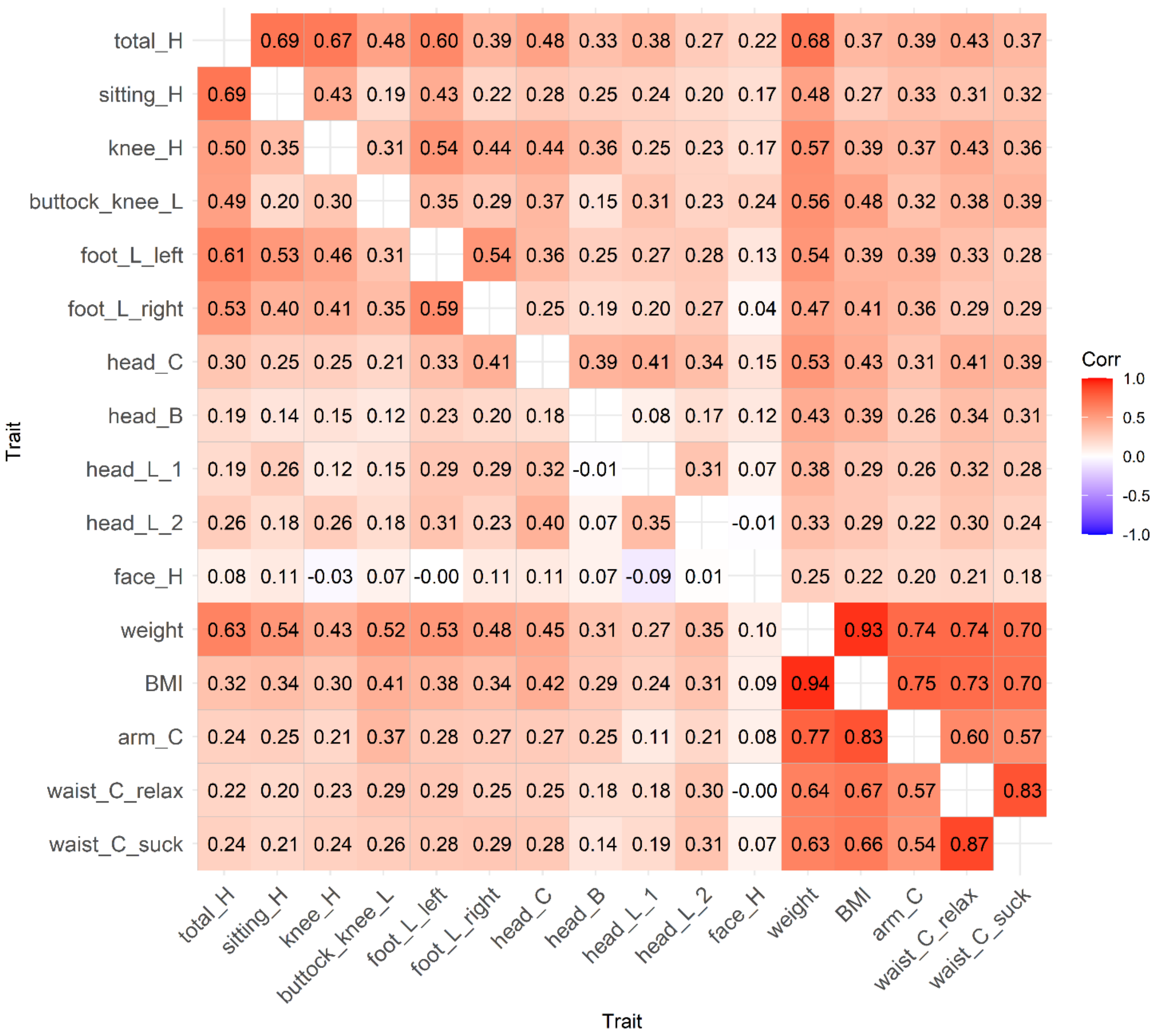


Supplemental Figure S2. Unique environmental correlations of anthropometric traits in males (upper diagonal matrix) and females (lower diagonal matrix) in the initial assessment. Abbreviations: B, breadth; C, circumference; H, height; L, length

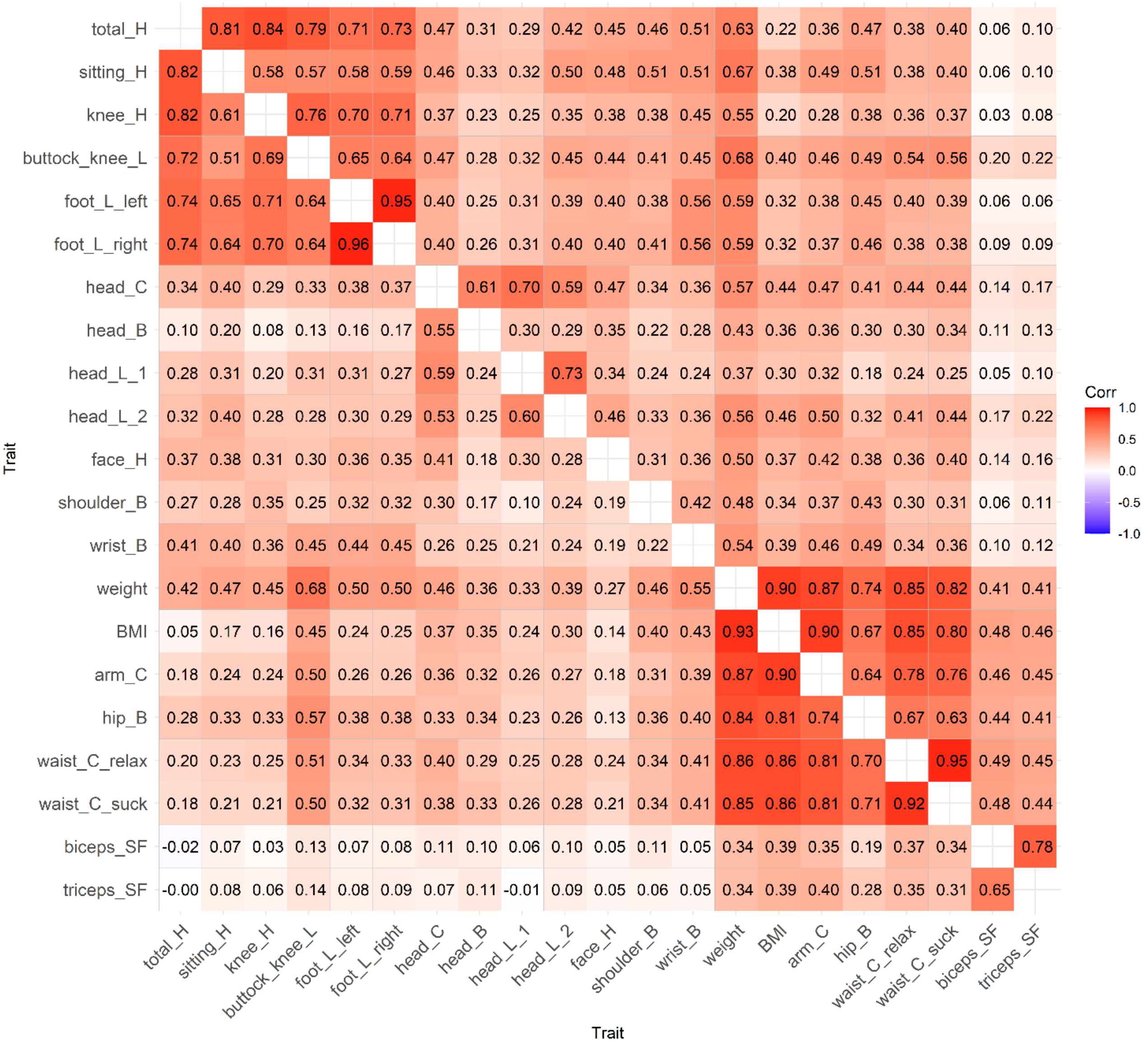


Supplemental Figure S3. Correlations of anthropometric traits in males (upper diagonal matrix) and females (lower diagonal matrix) in the follow-up assessment. Abbreviations: B, breadth; C, circumference; H, height; L, length, SF, skinfold

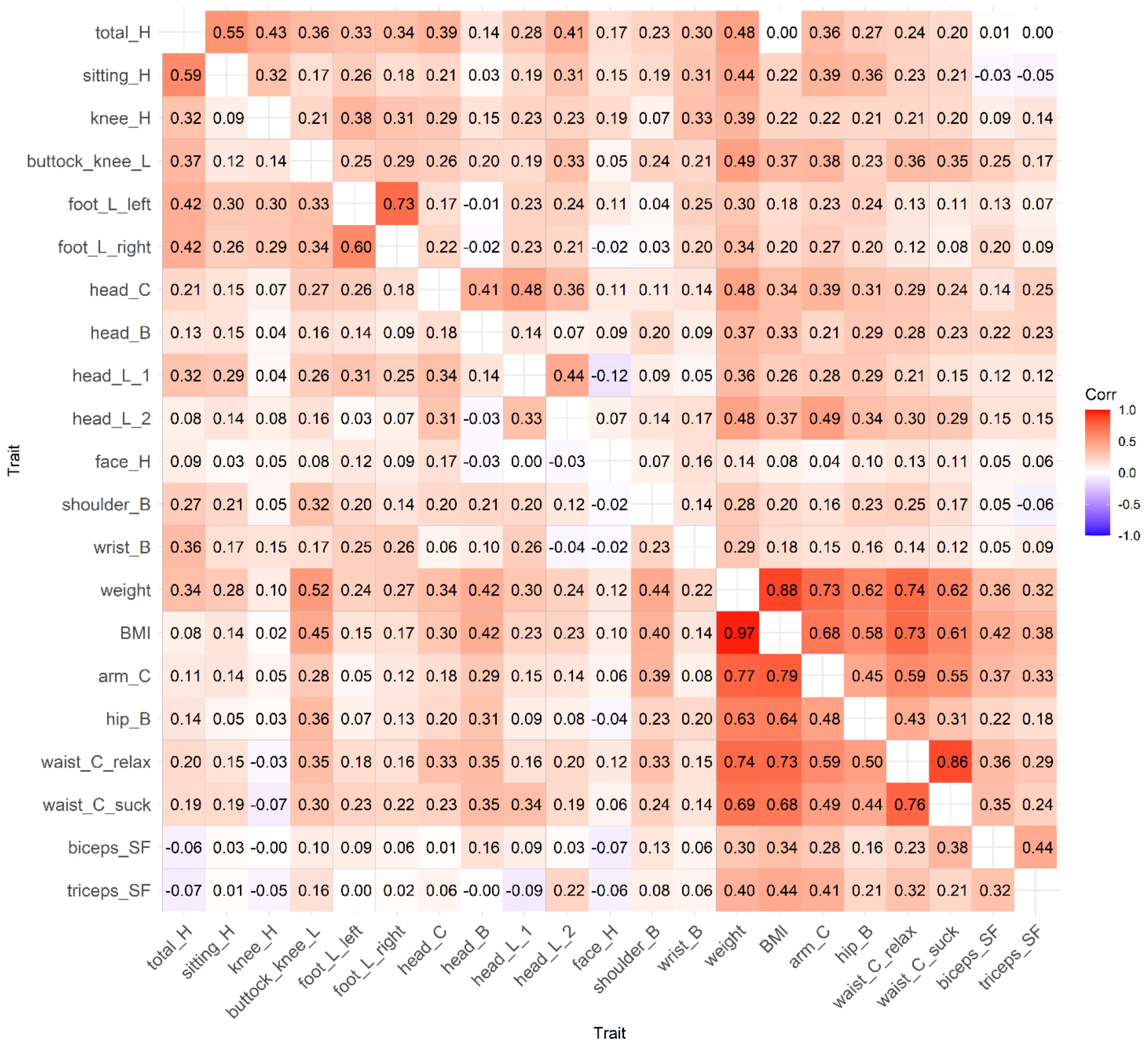


Supplemental Figure S4. Unique environmental correlations of anthropometric traits in males (upper diagonal matrix) and females (lower diagonal matrix) in the follow-up assessment. Abbreviations: B, breadth; C, circumference; H, height; L, length, SF, skinfold

Supplemental Table S1. Study protocol for anthropometric measures.

| Measure | Measurement protocol | Measurement device |
|---|---|---|
| Weight | Measured without shoes and heavy clothing removed. | scale |
| Height | The participant stands without shoes on a horizontal surface with his/her heels together, stretching upward to the fullest extent. The participant's back should be as straight as possible, which may be achieved by rounding or relaxing shoulders and manipulation the posture. Participant's heels should not leave the ground and should be placed against the wall or chair. | anthropometer |
| Sitting height | Measured from the bottom of the buttock to the top of head. One cm is added to the measured value. | anthropometer |
| Knee height | Measured from the bottom of the heel to the top of the leg on the dominant side with the thigh horizontal and having foot on the flat board. One cm is added to the measured value. | anthropometer |
| Buttock-knee length | Measured from the back of the chair to the front of the knee on the dominant side. The participant sits up straight against the back of the chair with their knees firmly against the seat. One cm is added to the measured value. | anthropometer |
| Foot length | Measured for both feet. Measured from the back of the heel to the front of the big toe, taken in stocking feet. | anthropometer |
| Face height | Measured from the tip of chin to the top of head teeth fully occluded. | anthropometer |
| Head length 1 | Measured as the maximum distance from the nasion to the back of the head. | anthropometer |
| Head length 2 | Measured as the maximum distance from the bottom of the nasion to the inion. The tissues are not compressed. | anthropometer |
| Head circumference | Measured around head immediately above eyebrows and ears. | metal tape |
| Head breadth | Measured as the breadth of maximum transverse plane, wherever is occurs, after using pressure to compress the tissues. | spreading calipers |
| Wrist breadth | Measured as the distance between the ulna and radial styloid processes using firm pressure. | spreading calipers |
| Shoulder breadth | Shoulder (biacromial) breadth is measured as the width between the most lateral borders of the acromial processes. The participant stands with heels together and hands by sides. Measure from behind. | anthropometer |
| Hip breadth | Hip (biiliac) breadth is measured as the distance between the iliac crests. The caliper is applied at a downward angle of 45 degrees to separate and compress the tissues. The participant is standing with feet 5 cm apart and arms across the chest. | anthropometer |
| Waist circumference relaxed | Measured as the horizontal plane at the umbilicus while person has their stomach relaxed. Measured in standing position directly on the skin. | metal tape |
| Waist circumference sucking | Measured as the horizontal plane at the umbilicus while person sucks in his/her stomach as far as can. Measured after waist circumference relaxed. Measured in standing position directly on the skin. | metal tape |

| | | |
|---|---|---|
| Arm circumference | Measured in a horizontal plane at the midpoint of the upper arm area – halfway between the shoulder and the elbow. The participant's arm should be flexed tight for the first measure, relaxed and by person's side for the second. | metal tape |
| Biceps skinfold | Measured in the middle of the posterior aspect of the arm over the tricep muscle. The arm should be hanging loose and relaxed. The average of two measures is recorded. | spreading calipers |
| Triceps skinfold | Measured in the middle of the posterior aspect of the arm over the tricep muscle. The arm is hanging loose and relaxed. The average of two measures is recorded. | spreading calipers |

Supplemental Table S2. Statistics for logarithmic transformation and age effects for anthropometric traits.

| Measure | Skewness | Skewness after logarithmic transformation | Logarithmic transformation used | P-value of age effect | Number of outliers |
|---|---|---|---|---|---|
| **Initial assessment** | | | | | |
| Height | 0.20681 | 0.06826 | NO | <0.00001 | 0 |
| Sitting height | 0.33770 | 0.19467 | NO | <0.00001 | 1 |
| Knee height | 0.21221 | 0.02273 | NO | <0.00001 | 2 |
| Buttock-knee length | 0.20001 | -0.01120 | NO | <0.00001 | 1 |
| Foot length left | 0.25751 | 0.06981 | NO | <0.00001 | 1 |
| Foot length right | 0.31556 | 0.11295 | NO | <0.00001 | 0 |
| Head circumference | 0.07789 | -0.03075 | NO | 0.015 | 1 |
| Head breadth | -0.01070 | -0.13002 | NO | 0.100 | 9 |
| Face height | -0.11140 | -0.32368 | NO | <0.00001 | 0 |
| Head length 1 | -0.14780 | -0.30138 | NO | 0.035 | 1 |
| Head length 2 | 0.12757 | -0.04258 | NO | 0.045 | 0 |
| Weight | 1.46121 | 0.73342 | YES | <0.00001 | 0 |
| BMI | 1.53304 | 0.97093 | YES | 0.552 | 0 |
| Arm circumference | 1.05239 | 0.64279 | NO | <0.00001 | 3 |
| Waist circ. relaxed | 1.26909 | 0.85869 | YES | <0.00001 | 0 |
| Waist circ. sucking | 1.22405 | 0.78253 | YES | <0.00001 | 0 |
| **Follow-up assessment for males** | | | | | |
| Height | 0.17294 | 0.03657 | NO | <0.00001 | 0 |
| Sitting height | -0.28346 | -0.28346 | NO | <0.00001 | 0 |
| Knee height | 0.06423 | -0.10572 | NO | <0.00001 | 0 |
| Buttock-knee length | -0.03234 | -0.19938 | NO | <0.00001 | 0 |
| Foot length left | 0.02915 | -0.10572 | NO | <0.00001 | 0 |
| Foot length right | 0.08507 | -0.10580 | NO | <0.00001 | 0 |
| Head circumference | 0.09074 | 0.00517 | NO | 0.242 | 0 |
| Head breadth | -0.06017 | -0.18740 | NO | 0.291 | 1 |
| Face height | -0.02756 | -0.16629 | NO | <0.00001 | 0 |
| Head length 1 | -0.13021 | -0.25868 | NO | <0.00001 | 1 |
| Head length 2 | 0.06905 | -0.06367 | NO | <0.00001 | 1 |
| Shoulder breadth | 0.20085 | -0.10882 | NO | <0.00001 | 0 |
| Wrist breadth | 0.10455 | -0.13601 | NO | 0.001 | 0 |
| Weight | 1.24317 | 0.52865 | YES | 0.001 | 0 |
| BMI | 1.44403 | 0.86741 | YES | 0.032 | 0 |
| Arm circumference | 0.87145 | 0.40495 | NO | <0.00001 | 0 |
| Hip breadth | 0.68054 | 0.26909 | NO | <0.00001 | 0 |
| Waist circ. relaxed | 1.41768 | 1.02827 | YES | 0.007 | 0 |
| Waist circ. sucking | 1.31779 | 0.90657 | YES | 0.003 | 0 |
| Biceps skinfold | 1.91386 | 0.45650 | YES | 0.476 | |
| Triceps skinfold | 1.57579 | 0.25389 | YES | 0.831 | 0 |

| **Follow-up assessment for females** | | | | | |
|---|---|---|---|---|---|
| Height | 0.05822 | -0.03226 | NO | 0.354 | 0 |
| Sitting height | 0.04320 | -0.05048 | NO | 0.367 | 2 |
| Knee height | 0.19595 | 0.01808 | NO | 0.947 | 2 |
| Buttock-knee length | 0.11057 | -0.06208 | NO | 0.645 | 1 |
| Foot length left | 0.13206 | -0.03484 | NO | 0.975 | 2 |
| Foot length right | 0.12175 | -0.04558 | NO | 0.670 | 2 |
| Head circumference | -0.14968 | -0.25883 | NO | 0.673 | 4 |
| Head breadth | 0.42667 | 0.27368 | NO | 0.386 | 0 |
| Face height | -0.09518 | -0.23922 | NO | 0.071 | 0 |
| Head length 1 | -0.06203 | -0.25909 | NO | 0.309 | 2 |
| Head length 2 | 0.37030 | 0.18611 | NO | 0.428 | 3 |
| Shoulder breadth | 0.34371 | 0.09186 | NO | 0.046 | 3 |
| Wrist breadth | 0.30020 | 0.07622 | NO | 0.987 | 2 |
| Weight | 1.65467 | 0.93462 | YES | 0.164 | 0 |
| BMI | 1.67313 | 1.09347 | YES | 0.061 | 0 |
| Arm circumference | 0.92533 | 0.45976 | NO | 0.001 | 0 |
| Hip breadth | 0.96026 | 0.56471 | NO | 0.419 | 2 |
| Waist circ. relaxed | 1.06768 | 0.69458 | YES | 0.012 | 0 |
| Waist circ. sucking | 1.15286 | 0.76190 | YES | 0.015 | 0 |
| Biceps skinfold | 1.16981 | -0.46723 | YES | 0.605 | 0 |
| Triceps skinfold | 0.38297 | -1.23659 | YES | 0.059 | 0 |

Supplemental Table S3. Within pair correlations for anthropometric traits in the initial and follow-up assessments by sex and zygosity.

| | Initial assessment | | | | Follow-up assessment | | | |
|---|---|---|---|---|---|---|---|---|
| | Males | | Females | | Males | | Females | |
| | MZ | DZ | MZ | DZ | MZ | DZ | MZ | DZ |
| Height | 0.93 | 0.52 | 0.93 | 0.60 | 0.90 | 0.54 | 0.95 | 0.51 |
| Sitting height | 0.87 | 0.44 | 0.90 | 0.57 | 0.86 | 0.50 | 0.88 | 0.35 |
| Knee height | 0.92 | 0.53 | 0.90 | 0.55 | 0.86 | 0.56 | 0.82 | 0.44 |
| Buttock-knee length | 0.83 | 0.64 | 0.87 | 0.53 | 0.83 | 0.61 | 0.83 | 0.46 |
| Foot length left | 0.91 | 0.48 | 0.89 | 0.56 | 0.84 | 0.48 | 0.90 | 0.40 |
| Foot length right | 0.92 | 0.51 | 0.88 | 0.54 | 0.84 | 0.50 | 0.88 | 0.45 |
| Head circumference | 0.84 | 0.58 | 0.86 | 0.44 | 0.83 | 0.54 | 0.85 | 0.40 |
| Head breadth | 0.81 | 0.52 | 0.87 | 0.57 | 0.83 | 0.56 | 0.84 | 0.41 |
| Head length 1 | 0.76 | 0.48 | 0.79 | 0.44 | 0.79 | 0.55 | 0.66 | 0.49 |
| Head length 2 | 0.81 | 0.50 | 0.82 | 0.59 | 0.82 | 0.60 | 0.66 | 0.41 |
| Face height | 0.70 | 0.41 | 0.62 | 0.32 | 0.64 | 0.50 | 0.68 | 0.32 |
| Weight | 0.90 | 0.56 | 0.92 | 0.57 | 0.90 | 0.59 | 0.89 | 0.53 |
| BMI | 0.90 | 0.57 | 0.91 | 0.55 | 0.88 | 0.56 | 0.88 | 0.58 |
| Arm circumference | 0.87 | 0.54 | 0.90 | 0.51 | 0.88 | 0.54 | 0.77 | 0.47 |
| Waist circ. relaxed | 0.84 | 0.62 | 0.85 | 0.53 | 0.82 | 0.55 | 0.84 | 0.36 |
| Waist circ. sucking | 0.84 | 0.55 | 0.86 | 0.53 | 0.79 | 0.58 | 0.80 | 0.55 |
| Shoulder breadth | NA | NA | NA | NA | 0.81 | 0.68 | 0.86 | 0.76 |
| Wrist breadth | NA | NA | NA | NA | 0.77 | 0.47 | 0.80 | 0.41 |
| Hip breadth | NA | NA | NA | NA | 0.89 | 0.62 | 0.85 | 0.58 |
| Biceps skinfold | NA | NA | NA | NA | 0.82 | 0.58 | 0.74 | 0.69 |
| Triceps skinfold | NA | NA | NA | NA | 0.82 | 0.63 | 0.68 | 0.64 |

Supplemental Table S4. Model fit statistics of anthropometric traits comparing different genetic models in the initial and follow-up assessments.

| | Saturated model | | Full ACE model[1] | | ACE model with same parameters for both sexes[2] | | Full AE model[3] | |
|---|---|---|---|---|---|---|---|---|
| | -2 LL | d.f. | Δ -2 LL | p-value | Δ -2 LL | p-value | Δ -2 LL | p-value |
| **Initial assessment** | | | | | | | | |
| Height | 8828 | 1472 | 13.2 | 0.3572 | 0.6 | 0.8916 | 5.2 | 0.0761 |
| Sitting height | 7162 | 1466 | 17.8 | 0.1206 | 9.7 | 0.0216 | 4.2 | 0.1198 |
| Knee height | 6161 | 1468 | 11.2 | 0.5088 | 3.9 | 0.2692 | 2.9 | 0.2357 |
| Buttock-knee | 6933 | 1470 | 13.4 | 0.3421 | 8.5 | 0.0360 | 14.1 | 0.0009 |
| Foot length left | 4143 | 1468 | 13.4 | 0.3429 | 7.1 | 0.0696 | 3.9 | 0.1406 |
| Foot length right | 4129 | 1472 | 10.9 | 0.5396 | 10.7 | 0.0134 | 3.8 | 0.1514 |
| Head circ. | 11866 | 1462 | 18.7 | 0.0966 | 9.3 | 0.0256 | 6.7 | 0.0353 |
| Head breadth | 8206 | 1462 | 106.5 | <0.0001 | 6.6 | 0.0858 | 1.7 | 0.4261 |
| Head length 1 | 2861 | 1470 | 13.8 | 0.3139 | 14.8 | 0.0020 | 2.4 | 0.2949 |
| Head length 2 | 3305 | 1472 | 13.9 | 0.3076 | 6.8 | 0.0777 | 11.3 | 0.0035 |
| Face height | 4102 | 1472 | 35.7 | 0.0004 | 7.2 | 0.0655 | 5.5 | 0.0637 |
| Weight | 5509 | 1470 | 31.4 | 0.0017 | 6.6 | 0.0873 | 2.5 | 0.2859 |
| BMI | 4799 | 1470 | 35.8 | 0.0003 | 9.0 | 0.0289 | 1.7 | 0.4374 |
| Arm circ. | 6651 | 1466 | 24.7 | 0.0164 | 7.0 | 0.0733 | 1.1 | 0.5664 |
| Waist circ. relax | 4327 | 1472 | 25.4 | 0.0129 | 5.4 | 0.1473 | 8.6 | 0.0137 |
| Waist circ. suck | 4515 | 1470 | 24.5 | 0.0174 | 5.8 | 0.1237 | 4.0 | 0.1363 |
| **Follow-up assessment** | | | | | | | | |
| Height | 6742 | 1102 | 23.2 | 0.0263 | 53.2 | <0.0001 | 4.1 | 0.1297 |
| Sitting height | 5472 | 1078 | 18.1 | 0.1141 | 42.3 | <0.0001 | 1.5 | 0.4794 |
| Knee height | 4865 | 1096 | 10.7 | 0.5527 | 4.4 | 0.2209 | 3.5 | 0.1770 |
| Buttock-knee len | 5242 | 1104 | 19.9 | 0.0698 | 5.5 | 0.1376 | 8.5 | 0.0143 |
| Foot length left | 3226 | 1104 | 19.8 | 0.0703 | 23.0 | <0.0001 | 0.9 | 0.6375 |
| Foot length right | 3231 | 1102 | 12.4 | 0.4177 | 12.1 | 0.0072 | 1.3 | 0.5281 |
| Head circ | 3868 | 1098 | 14.3 | 0.2836 | 2.9 | 0.4106 | 2.9 | 0.2298 |
| Head breadth | 6392 | 1104 | 67.2 | 0.0000 | 1.7 | 0.6331 | 1.1 | 0.5778 |
| Head length 1 | 7546 | 1102 | 11.3 | 0.5064 | 7.5 | 0.0564 | 7.3 | 0.0256 |
| Head length 2 | 7700 | 1102 | 24.0 | 0.0202 | 13.2 | 0.0043 | 9.6 | 0.0081 |
| Face height | 3013 | 1104 | 11.5 | 0.4840 | 3.4 | 0.3297 | 6.5 | 0.0396 |
| Shoulder breadth | 4969 | 1102 | 16.7 | 0.1607 | 5.9 | 0.1169 | 42.9 | 0.0000 |
| Wrist breadth | 5467 | 1104 | 11.4 | 0.4943 | 0.7 | 0.8653 | 1.7 | 0.4324 |
| Weight | 4057 | 1106 | 18.6 | 0.0981 | 1.7 | 0.6291 | 3.5 | 0.1722 |
| BMI | 3702 | 1100 | 17.6 | 0.1298 | 15.0 | 0.0018 | 3.9 | 0.1417 |
| Arm circ | 5286 | 1082 | 22.0 | 0.0372 | 30.5 | <0.0001 | 1.0 | 0.6094 |
| Hip breadth | 5064 | 1104 | 13.2 | 0.3538 | 14.5 | 0.0023 | 10.9 | 0.0043 |
| Waist circ. relax | 3082 | 1084 | 12.9 | 0.3799 | 18.3 | 0.0004 | 2.3 | 0.3141 |
| Waist circ. suck | 3323 | 1084 | 12.0 | 0.4422 | 6.8 | 0.0772 | 9.3 | 0.0096 |
| Biceps skinfold | 6561 | 1072 | 10.7 | 0.5580 | 9.2 | 0.0268 | 19.8 | 0.0001 |
| Triceps skinfold | 6216 | 1076 | 11.8 | 0.4632 | 27.3 | <0.0001 | 18.2 | 0.0002 |

[1]Compared to the saturated model (Δ d.f. 12)
[2]Compared to the full ACE model (Δ d.f. 3)
[3]Compared to the full ACE model (Δ d.f. 2)

Abbreviations: -2LL (-2 log-likelihood); d.f. (degrees of freedom); Δ (change); ACE (additive genetic/ shared environment/ unique environment) model; AE (additive genetic/ unique environment) model

Supplemental Table S5. Explorative factor analysis of anthropometric traits in the initial assessment in males and females.[1]

| | Males | | | Females | | |
|---|---|---|---|---|---|---|
| | 1. factor | 2. factor | 3. factor | 1. factor | 2. factor | 3. factor |
| Eigenvalue | 8.76 | 1.91 | 1.16 | 8.60 | 2.01 | 1.22 |
| % of explained variation | 69 | 15 | 9 | 68 | 16 | 10 |
| Factor loadings | | | | | | |
| Height | **0.943** | 0.214 | 0.165 | 0.219 | **0.931** | 0.145 |
| Sitting height | **0.756** | 0.309 | 0.185 | 0.305 | **0.752** | 0.233 |
| Knee height | **0.854** | 0.232 | 0.124 | 0.225 | **0.859** | 0.032 |
| Buttock-knee length | **0.714** | 0.443 | 0.170 | 0.479 | **0.721** | 0.158 |
| Foot length left | **0.857** | 0.283 | 0.121 | 0.229 | **0.837** | 0.189 |
| Foot length right | **0.858** | 0.275 | 0.141 | 0.239 | **0.838** | 0.193 |
| Head circumference | 0.246 | 0.307 | **0.791** | 0.369 | 0.322 | **0.686** |
| Head breadth | 0.193 | 0.271 | 0.387 | 0.306 | 0.153 | 0.466 |
| Head length 1 | 0.184 | 0.178 | **0.726** | 0.134 | 0.186 | **0.754** |
| Head length 2 | 0.291 | 0.314 | 0.568 | 0.267 | 0.231 | **0.657** |
| Face height | 0.310 | 0.236 | 0.305 | 0.238 | 0.347 | 0.310 |
| Weight | 0.497 | **0.837** | 0.183 | **0.832** | 0.503 | 0.160 |
| BMI | 0.107 | **0.965** | 0.144 | **0.951** | 0.172 | 0.132 |
| Arm circumference | 0.257 | **0.880** | 0.119 | **0.884** | 0.228 | 0.162 |
| Waist circ. relaxed | 0.297 | **0.890** | 0.156 | **0.912** | 0.209 | 0.114 |
| Waist circ. sucking | 0.289 | **0.883** | 0.166 | **0.888** | 0.219 | 0.156 |

[1]Varimax rotation is used. The key factor loadings for each factor are bolded.

Supplemental Table S6. Correlations of anthropometric traits in males (upper diagonal matrix) and females (lower diagonal matrix) with 95% confidence intervals in the initial assessment.

| | 1 | 2 | 3 | 4 | 5 | 6 | 7 | 8 | 9 | 10 | 11 | 12 | 13 | 14 | 15 | 16 |
|---|---|---|---|---|---|---|---|---|---|---|---|---|---|---|---|---|
| 1 | | 0.86<br>0.84,<br>0.88 | 0.90<br>0.88,<br>0.91 | 0.82<br>0.80,<br>0.85 | 0.82<br>0.79,<br>0.84 | 0.82<br>0.80,<br>0.84 | 0.42<br>0.36,<br>0.47 | 0.29<br>0.22,<br>0.36 | 0.33<br>0.27,<br>0.39 | 0.42<br>0.36,<br>0.48 | 0.41<br>0.35,<br>0.47 | 0.67<br>0.63,<br>0.71 | 0.30<br>0.24,<br>0.37 | 0.44<br>0.38,<br>0.50 | 0.49<br>0.43,<br>0.54 | 0.49<br>0.43,<br>0.54 |
| 2 | 0.88<br>0.86,<br>0.89 | | 0.69<br>0.65,<br>0.72 | 0.62<br>0.57,<br>0.66 | 0.72<br>0.68,<br>0.75 | 0.71<br>0.67,<br>0.74 | 0.42<br>0.36,<br>0.48 | 0.31<br>0.24,<br>0.37 | 0.31<br>0.25,<br>0.37 | 0.41<br>0.35,<br>0.47 | 0.43<br>0.36,<br>0.48 | 0.69<br>0.66,<br>0.73 | 0.41<br>0.35,<br>0.47 | 0.50<br>0.45,<br>0.56 | 0.50<br>0.45,<br>0.55 | 0.49<br>0.43,<br>0.54 |
| 3 | 0.88<br>0.86,<br>0.89 | 0.68<br>0.64,<br>0.71 | | 0.78<br>0.75,<br>0.81 | 0.77<br>0.74,<br>0.80 | 0.78<br>0.75,<br>0.81 | 0.38<br>0.32,<br>0.44 | 0.29<br>0.23,<br>0.36 | 0.29<br>0.23,<br>0.36 | 0.37<br>0.31,<br>0.43 | 0.37<br>0.31,<br>0.43 | 0.63<br>0.58,<br>0.67 | 0.30<br>0.24,<br>0.37 | 0.43<br>0.37,<br>0.49 | 0.48<br>0.42,<br>0.53 | 0.48<br>0.43,<br>0.54 |
| 4 | 0.83<br>0.80,<br>0.85 | 0.67<br>0.63,<br>0.71 | 0.79<br>0.76,<br>0.82 | | 0.71<br>0.68,<br>0.75 | 0.72<br>0.68,<br>0.75 | 0.42<br>0.36,<br>0.48 | 0.29<br>0.22,<br>0.35 | 0.34<br>0.27,<br>0.40 | 0.47<br>0.41,<br>0.52 | 0.41<br>0.35,<br>0.47 | 0.75<br>0.72,<br>0.78 | 0.50<br>0.44,<br>0.55 | 0.56<br>0.51,<br>0.61 | 0.64<br>0.60,<br>0.68 | 0.65<br>0.60,<br>0.69 |
| 5 | 0.76<br>0.73,<br>0.79 | 0.66<br>0.61,<br>0.69 | 0.74<br>0.71,<br>0.77 | 0.70<br>0.67,<br>0.74 | | 0.95<br>0.94,<br>0.96 | 0.38<br>0.32,<br>0.44 | 0.29<br>0.22,<br>0.35 | 0.32<br>0.26,<br>0.38 | 0.42<br>0.36,<br>0.47 | 0.33<br>0.26,<br>0.39 | 0.67<br>0.63,<br>0.71 | 0.40<br>0.34,<br>0.46 | 0.47<br>0.42,<br>0.53 | 0.52<br>0.47,<br>0.57 | 0.50<br>0.45,<br>0.55 |
| 6 | 0.77<br>0.74,<br>0.80 | 0.67<br>0.63,<br>0.71 | 0.74<br>0.71,<br>0.77 | 0.70<br>0.66,<br>0.73 | 0.95<br>0.94,<br>0.95 | | 0.39<br>0.33,<br>0.45 | 0.30<br>0.23,<br>0.36 | 0.34<br>0.27,<br>0.40 | 0.44<br>0.38,<br>0.49 | 0.34<br>0.27,<br>0.40 | 0.67<br>0.63,<br>0.71 | 0.40<br>0.34,<br>0.46 | 0.46<br>0.40,<br>0.52 | 0.52<br>0.46,<br>0.57 | 0.51<br>0.45,<br>0.56 |
| 7 | 0.49<br>0.44,<br>0.55 | 0.51<br>0.45,<br>0.56 | 0.39<br>0.33,<br>0.45 | 0.53<br>0.48,<br>0.58 | 0.46<br>0.40,<br>0.52 | 0.48<br>0.43,<br>0.54 | | 0.55<br>0.49,<br>0.59 | 0.65<br>0.61,<br>0.69 | 0.56<br>0.51,<br>0.61 | 0.40<br>0.34,<br>0.46 | 0.52<br>0.47,<br>0.57 | 0.43<br>0.37,<br>0.49 | 0.43<br>0.37,<br>0.49 | 0.47<br>0.42,<br>0.53 | 0.47<br>0.41,<br>0.52 |
| 8 | 0.29<br>0.22,<br>0.35 | 0.32<br>0.25,<br>0.38 | 0.20<br>0.13,<br>0.27 | 0.33<br>0.27,<br>0.40 | 0.29<br>0.22,<br>0.35 | 0.29<br>0.22,<br>0.35 | 0.63<br>0.59,<br>0.67 | | 0.22<br>0.16,<br>0.29 | 0.26<br>0.20,<br>0.33 | 0.25<br>0.19,<br>0.32 | 0.39<br>0.32,<br>0.45 | 0.33<br>0.27,<br>0.40 | 0.34<br>0.27,<br>0.40 | 0.35<br>0.28,<br>0.41 | 0.34<br>0.27,<br>0.40 |
| 9 | 0.31<br>0.25,<br>0.38 | 0.33<br>0.26,<br>0.39 | 0.23<br>0.17,<br>0.30 | 0.35<br>0.28,<br>0.41 | 0.34<br>0.27,<br>0.40 | 0.34<br>0.27,<br>0.40 | 0.62<br>0.57,<br>0.66 | 0.33<br>0.26,<br>0.39 | | 0.61<br>0.57,<br>0.65 | 0.31<br>0.24,<br>0.37 | 0.37<br>0.31,<br>0.43 | 0.29<br>0.23,<br>0.36 | 0.30<br>0.24,<br>0.37 | 0.31<br>0.25,<br>0.38 | 0.32<br>0.25,<br>0.38 |
| 10 | 0.38<br>0.31,<br>0.44 | 0.42<br>0.36,<br>0.48 | 0.30<br>0.23,<br>0.37 | 0.39<br>0.33,<br>0.45 | 0.38<br>0.31,<br>0.44 | 0.39<br>0.33,<br>0.45 | 0.55<br>0.50,<br>0.60 | 0.32<br>0.26,<br>0.39 | 0.70<br>0.66,<br>0.74 | | 0.33<br>0.27,<br>0.39 | 0.50<br>0.45,<br>0.56 | 0.41<br>0.35,<br>0.47 | 0.39<br>0.33,<br>0.45 | 0.46<br>0.40,<br>0.51 | 0.46<br>0.40,<br>0.52 |

| | | | | | | | | | | | | | | | | |
|---|---|---|---|---|---|---|---|---|---|---|---|---|---|---|---|---|
| 11 | 0.41 0.35, 0.47 | 0.44 0.38, 0.49 | 0.31 0.24, 0.37 | 0.38 0.32, 0.44 | 0.42 0.36, 0.48 | 0.42 0.36, 0.48 | 0.45 0.39, 0.51 | 0.26 0.19, 0.33 | 0.30 0.23, 0.36 | 0.34 0.27, 0.40 | | 0.40 0.34, 0.46 | 0.29 0.22, 0.35 | 0.34 0.27, 0.40 | 0.33 0.26, 0.39 | 0.33 0.27, 0.39 |
| 12 | 0.69 0.65, 0.72 | 0.71 0.68, 0.75 | 0.62 0.58, 0.66 | 0.79 0.76, 0.81 | 0.62 0.57, 0.66 | 0.63 0.59, 0.67 | 0.59 0.54, 0.63 | 0.42 0.36, 0.48 | 0.32 0.26, 0.39 | 0.45 0.39, 0.50 | 0.41 0.35, 0.47 | | 0.91 0.89, 0.92 | 0.89 0.87, 0.90 | 0.89 0.88, 0.91 | 0.88 0.87, 0.90 |
| 13 | 0.37 0.31, 0.43 | 0.46 0.40, 0.52 | 0.35 0.29, 0.41 | 0.59 0.54, 0.63 | 0.40 0.34, 0.46 | 0.42 0.35, 0.47 | 0.50 0.44, 0.55 | 0.39 0.33, 0.45 | 0.26 0.19, 0.32 | 0.38 0.32, 0.44 | 0.32 0.25, 0.38 | 0.93 0.92, 0.94 | | 0.90 0.88, 0.91 | 0.88 0.86, 0.89 | 0.86 0.84, 0.88 |
| 14 | 0.43 0.36, 0.48 | 0.50 0.44, 0.55 | 0.39 0.33, 0.45 | 0.62 0.58, 0.66 | 0.42 0.36, 0.48 | 0.44 0.38, 0.49 | 0.50 0.44, 0.55 | 0.38 0.32, 0.44 | 0.29 0.22, 0.35 | 0.42 0.35, 0.47 | 0.32 0.25, 0.38 | 0.90 0.88, 0.91 | 0.93 0.92, 0.94 | | 0.84 0.82, 0.86 | 0.83 0.81, 0.85 |
| 15 | 0.42 0.36, 0.47 | 0.43 0.37, 0.49 | 0.40 0.34, 0.46 | 0.60 0.55, 0.64 | 0.43 0.37, 0.49 | 0.44 0.38, 0.49 | 0.48 0.42, 0.53 | 0.33 0.27, 0.40 | 0.27 0.20, 0.33 | 0.39 0.33, 0.45 | 0.34 0.28, 0.40 | 0.84 0.82, 0.86 | 0.87 0.85, 0.89 | 0.83 0.81, 0.85 | | 0.96 0.95, 0.96 |
| 16 | 0.42 0.36, 0.48 | 0.43 0.37, 0.49 | 0.41 0.34, 0.46 | 0.61 0.56, 0.66 | 0.44 0.38, 0.50 | 0.45 0.39, 0.50 | 0.49 0.43, 0.54 | 0.35 0.28, 0.41 | 0.29 0.23, 0.36 | 0.42 0.36, 0.48 | 0.35 0.29, 0.42 | 0.83 0.80, 0.85 | 0.84 0.82, 0.86 | 0.82 0.79, 0.84 | 0.96 0.96, 0.97 | |

1=total height, 2=sitting height, 3=knee height, 4=buttock-knee length, 5=foot length left
6=foot length right, 7=head circumference, 8=head breadth, 9=head length 1, 10=head length 2, 11=face height, 13=weight, 14=BMI, 15=arm circumference, 16=waist circumference relaxed, 17=waist circumference sucking

Supplemental Table S7. Additive genetic correlations of anthropometric traits in males (upper diagonal matrix) and females (lower diagonal matrix) with 95% confidence intervals in the initial assessment under the additive genetic / unique environmental model.

| | 1 | 2 | 3 | 4 | 5 | 6 | 7 | 8 | 9 | 10 | 11 | 12 | 13 | 14 | 15 | 16 |
|---|---|---|---|---|---|---|---|---|---|---|---|---|---|---|---|---|
| 1 | | 0.88<br>0.85,<br>0.90 | 0.91<br>0.89,<br>0.93 | 0.87<br>0.84,<br>0.90 | 0.83<br>0.80,<br>0.86 | 0.85<br>0.82,<br>0.88 | 0.42<br>0.33,<br>0.50 | 0.28<br>0.18,<br>0.37 | 0.34<br>0.25,<br>0.43 | 0.46<br>0.37,<br>0.53 | 0.48<br>0.39,<br>0.56 | 0.67<br>0.62,<br>0.72 | 0.30<br>0.21,<br>0.39 | 0.45<br>0.37,<br>0.52 | 0.50<br>0.42,<br>0.57 | 0.51<br>0.43,<br>0.58 |
| 2 | 0.89<br>0.87,<br>0.91 | | 0.71<br>0.66,<br>0.76 | 0.69<br>0.62,<br>0.74 | 0.75<br>0.70,<br>0.79 | 0.77<br>0.72,<br>0.81 | 0.44<br>0.36,<br>0.52 | 0.32<br>0.22,<br>0.41 | 0.34<br>0.24,<br>0.43 | 0.45<br>0.37,<br>0.53 | 0.51<br>0.42,<br>0.59 | 0.72<br>0.66,<br>0.76 | 0.43<br>0.34,<br>0.50 | 0.53<br>0.45,<br>0.60 | 0.53<br>0.45,<br>0.60 | 0.51<br>0.43,<br>0.59 |
| 3 | 0.92<br>0.90,<br>0.93 | 0.71<br>0.66,<br>0.76 | | 0.85<br>0.81,<br>0.88 | 0.79<br>0.75,<br>0.82 | 0.80<br>0.76,<br>0.83 | 0.38<br>0.30,<br>0.47 | 0.28<br>0.18,<br>0.37 | 0.32<br>0.22,<br>0.41 | 0.40<br>0.31,<br>0.48 | 0.44<br>0.34,<br>0.52 | 0.63<br>0.57,<br>0.68 | 0.30<br>0.21,<br>0.38 | 0.43<br>0.35,<br>0.51 | 0.48<br>0.41,<br>0.56 | 0.50<br>0.42,<br>0.57 |
| 4 | 0.86<br>0.83,<br>0.89 | 0.72<br>0.67,<br>0.77 | 0.86<br>0.82,<br>0.89 | | 0.76<br>0.71,<br>0.81 | 0.78<br>0.73,<br>0.82 | 0.44<br>0.35,<br>0.52 | 0.32<br>0.22,<br>0.41 | 0.35<br>0.25,<br>0.44 | 0.52<br>0.44,<br>0.60 | 0.47<br>0.37,<br>0.55 | 0.78<br>0.73,<br>0.82 | 0.51<br>0.43,<br>0.58 | 0.60<br>0.53,<br>0.66 | 0.69<br>0.62,<br>0.74 | 0.68<br>0.62,<br>0.74 |
| 5 | 0.77<br>0.73,<br>0.81 | 0.66<br>0.61,<br>0.72 | 0.78<br>0.73,<br>0.81 | 0.76<br>0.71,<br>0.80 | | 0.99<br>0.98,<br>0.99 | 0.39<br>0.31,<br>0.48 | 0.29<br>0.19,<br>0.38 | 0.35<br>0.25,<br>0.44 | 0.44<br>0.35,<br>0.52 | 0.39<br>0.30,<br>0.48 | 0.68<br>0.62,<br>0.73 | 0.40<br>0.32,<br>0.48 | 0.48<br>0.40,<br>0.55 | 0.54<br>0.47,<br>0.61 | 0.53<br>0.45,<br>0.60 |
| 6 | 0.80<br>0.76,<br>0.83 | 0.71<br>0.65,<br>0.75 | 0.79<br>0.74,<br>0.82 | 0.76<br>0.71,<br>0.80 | 1.00<br>0.99,<br>NA | | 0.41<br>0.33,<br>0.49 | 0.30<br>0.21,<br>0.39 | 0.38<br>0.28,<br>0.46 | 0.47<br>0.39,<br>0.55 | 0.42<br>0.32,<br>0.51 | 0.68<br>0.63,<br>0.73 | 0.40<br>0.31,<br>0.47 | 0.47<br>0.39,<br>0.54 | 0.54<br>0.46,<br>0.61 | 0.53<br>0.46,<br>0.60 |
| 7 | 0.53<br>0.45,<br>0.60 | 0.55<br>0.47,<br>0.61 | 0.43<br>0.35,<br>0.51 | 0.59<br>0.52,<br>0.66 | 0.49<br>0.41,<br>0.56 | 0.50<br>0.42,<br>0.57 | | 0.58<br>0.51,<br>0.65 | 0.72<br>0.65,<br>0.77 | 0.60<br>0.52,<br>0.66 | 0.48<br>0.39,<br>0.57 | 0.52<br>0.45,<br>0.59 | 0.44<br>0.36,<br>0.52 | 0.45<br>0.37,<br>0.53 | 0.48<br>0.40,<br>0.56 | 0.47<br>0.39,<br>0.55 |
| 8 | 0.31<br>0.22,<br>0.40 | 0.34<br>0.25,<br>0.42 | 0.22<br>0.12,<br>0.31 | 0.37<br>0.28,<br>0.46 | 0.29<br>0.20,<br>0.38 | 0.29<br>0.20,<br>0.38 | 0.71<br>0.64,<br>0.76 | | 0.26<br>0.15,<br>0.36 | 0.27<br>0.16,<br>0.37 | 0.30<br>0.19,<br>0.40 | 0.38<br>0.29,<br>0.46 | 0.33<br>0.24,<br>0.42 | 0.36<br>0.26,<br>0.44 | 0.34<br>0.25,<br>0.43 | 0.34<br>0.25,<br>0.43 |
| 9 | 0.35<br>0.25,<br>0.43 | 0.34<br>0.25,<br>0.43 | 0.27<br>0.17,<br>0.36 | 0.40<br>0.31,<br>0.49 | 0.36<br>0.27,<br>0.45 | 0.36<br>0.27,<br>0.45 | 0.70<br>0.63,<br>0.75 | 0.40<br>0.31,<br>0.49 | | 0.69<br>0.62,<br>0.75 | 0.39<br>0.28,<br>0.49 | 0.39<br>0.30,<br>0.48 | 0.31<br>0.22,<br>0.40 | 0.32<br>0.22,<br>0.41 | 0.32<br>0.22,<br>0.41 | 0.32<br>0.22,<br>0.42 |
| 10 | 0.40<br>0.32,<br>0.48 | 0.45<br>0.37,<br>0.53 | 0.32<br>0.22,<br>0.40 | 0.45<br>0.36,<br>0.53 | 0.39<br>0.30,<br>0.47 | 0.42<br>0.33,<br>0.50 | 0.59<br>0.52,<br>0.65 | 0.36<br>0.27,<br>0.45 | 0.79<br>0.73,<br>0.83 | | 0.44<br>0.34,<br>0.54 | 0.53<br>0.46,<br>0.60 | 0.43<br>0.34,<br>0.51 | 0.42<br>0.33,<br>0.50 | 0.48<br>0.40,<br>0.56 | 0.50<br>0.41,<br>0.58 |

| | | | | | | | | | | | | | | | | |
|---|---|---|---|---|---|---|---|---|---|---|---|---|---|---|---|---|
| 11 | 0.52 0.43, 0.61 | 0.56 0.46, 0.64 | 0.43 0.33, 0.53 | 0.50 0.40, 0.60 | 0.57 0.47, 0.66 | 0.54 0.44, 0.63 | 0.59 0.49, 0.68 | 0.33 0.22, 0.44 | 0.46 0.35, 0.58 | 0.46 0.35, 0.56 | | 0.47 0.37, 0.55 | 0.33 0.23, 0.43 | 0.39 0.29, 0.48 | 0.38 0.28, 0.47 | 0.39 0.29, 0.48 |
| 12 | 0.68 0.63, 0.73 | 0.72 0.67, 0.77 | 0.64 0.57, 0.69 | 0.82 0.78, 0.85 | 0.61 0.55, 0.67 | 0.65 0.59, 0.70 | 0.62 0.55, 0.68 | 0.44 0.36, 0.52 | 0.36 0.27, 0.44 | 0.48 0.40, 0.55 | 0.54 0.44, 0.62 | | 0.91 0.89, 0.92 | 0.90 0.88, 0.92 | 0.92 0.90, 0.94 | 0.91 0.89, 0.93 |
| 13 | 0.37 0.28, 0.45 | 0.47 0.39, 0.54 | 0.35 0.26, 0.43 | 0.61 0.54, 0.67 | 0.39 0.31, 0.47 | 0.42 0.34, 0.50 | 0.52 0.44, 0.59 | 0.41 0.32, 0.49 | 0.28 0.18, 0.37 | 0.41 0.32, 0.49 | 0.42 0.32, 0.52 | 0.93 0.92, 0.94 | | 0.91 0.89, 0.93 | 0.90 0.88, 0.92 | 0.89 0.86, 0.91 |
| 14 | 0.43 0.35, 0.51 | 0.52 0.44, 0.59 | 0.40 0.32, 0.48 | 0.65 0.59, 0.71 | 0.43 0.35, 0.51 | 0.46 0.38, 0.53 | 0.55 0.47, 0.61 | 0.40 0.32, 0.48 | 0.34 0.24, 0.43 | 0.46 0.37, 0.54 | 0.42 0.31, 0.51 | 0.91 0.89, 0.93 | 0.94 0.93, 0.95 | | 0.88 0.85, 0.90 | 0.87 0.84, 0.90 |
| 15 | 0.44 0.36, 0.52 | 0.46 0.37, 0.53 | 0.43 0.34, 0.50 | 0.65 0.59, 0.71 | 0.45 0.36, 0.52 | 0.47 0.38, 0.54 | 0.53 0.45, 0.60 | 0.37 0.28, 0.45 | 0.29 0.19, 0.39 | 0.42 0.33, 0.50 | 0.49 0.38, 0.58 | 0.88 0.85, 0.90 | 0.90 0.88, 0.92 | 0.87 0.84, 0.90 | | 0.98 0.97, 0.99 |
| 16 | 0.44 0.36, 0.52 | 0.45 0.37, 0.53 | 0.42 0.34, 0.50 | 0.66 0.60, 0.72 | 0.45 0.37, 0.53 | 0.46 0.38, 0.54 | 0.54 0.46, 0.60 | 0.39 0.31, 0.48 | 0.32 0.22, 0.41 | 0.45 0.37, 0.53 | 0.47 0.37, 0.57 | 0.85 0.82, 0.88 | 0.87 0.84, 0.89 | 0.85 0.82, 0.88 | 0.98 0.97, 0.98 | |

1=total height, 2=sitting height, 3=knee height, 4=buttock-knee length, 5=foot length left
6=foot length right, 7=head circumference, 8=head breadth, 9=head length 1, 10=head length 2, 11=face height, 13=weight, 14=BMI, 15=arm circumference, 16=waist circumference relaxed, 17=waist circumference sucking

Supplemental Table S8. Additive genetic correlations of anthropometric traits in males (upper diagonal matrix) and females (lower diagonal matrix) with 95% confidence intervals in the initial assessment under additive genetic/ shared environment/ unique environment model.

| | 1 | 2 | 3 | 4 | 5 | 6 | 7 | 8 | 9 | 10 | 11 | 12 | 13 | 14 | 15 | 16 |
|---|---|---|---|---|---|---|---|---|---|---|---|---|---|---|---|---|
| 1 | | 0.89 0.82, 0.94 | 0.90 0.86, 0.94 | 0.96 0.86, 1.00 | 0.84 0.76, 0.90 | 0.88 0.80, 0.93 | 0.32 0.09, 0.52 | 0.10 -0.10, 0.28 | 0.43 0.19, 0.62 | 0.57 0.35, 0.75 | 0.52 0.18, 1.00 | 0.68 0.55, 0.77 | 0.32 0.13, 0.49 | 0.48 0.29, 0.63 | 0.48 0.27, 0.64 | 0.48 0.48, 0.64 |
| 2 | 0.88 0.82, 0.93 | | 0.72 0.59, 0.82 | 0.79 0.58, 0.97 | 0.73 0.63, 0.83 | 0.80 0.68, 0.89 | 0.39 0.15, 0.61 | 0.17 -0.05, 0.35 | 0.44 0.18, 0.65 | 0.54 0.29, 0.75 | 0.57 0.23, 1.00 | 0.76 0.63, 0.85 | 0.48 0.29, 0.63 | 0.56 0.37, 0.71 | 0.56 0.35, 0.71 | 0.54 0.33, 0.70 |
| 3 | 0.97 0.92, 1.00 | 0.69 0.56, 0.80 | | 0.96 0.84, 1.00 | 0.80 0.71, 0.87 | 0.82 0.74, 0.89 | 0.38 0.16, 0.58 | 0.16 -0.03, 0.34 | 0.45 0.22, 0.64 | 0.45 0.22, 0.64 | 0.52 0.18, 1.00 | 0.64 0.50, 0.74 | 0.32 0.13, 0.48 | 0.48 0.30, 0.62 | 0.47 0.27, 0.63 | 0.49 0.31, 0.65 |
| 4 | 0.84 0.78, 0.89 | 0.66 0.55, 0.77 | 0.89 0.79, 0.96 | | 0.83 0.68, 0.97 | 0.84 0.69, 0.98 | 0.39 0.08, 0.64 | 0.21 -0.08, 0.48 | 0.59 0.30, 0.81 | 0.70 0.44, 0.93 | 0.31 0.31, 0.65 | 0.77 0.64, 0.88 | 0.45 0.45, 0.64 | 0.61 0.39, 0.79 | 0.54 0.48, 0.74 | 0.54 0.54, 0.71 |
| 5 | 0.83 0.74, 0.89 | 0.68 0.54, 0.79 | 0.84 0.74, 0.92 | 0.75 0.63, 0.86 | | 0.99 0.98, 1.00 | 0.28 0.06, 0.48 | 0.17 -0.02, 0.35 | 0.44 0.20, 0.64 | 0.35 0.15, 0.56 | 0.49 0.14, 1.00 | 0.64 0.53, 0.73 | 0.33 0.18, 0.48 | 0.43 0.28, 0.58 | 0.44 0.27, 0.59 | 0.45 0.30, 0.59 |
| 6 | 0.90 0.82, 0.96 | 0.75 0.61, 0.85 | 0.88 0.79, 0.96 | 0.77 0.64, 0.88 | 1.00 NA, NA | | 0.30 0.10, 0.50 | 0.17 -0.02, 0.34 | 0.48 0.25, 0.67 | 0.45 0.23, 0.64 | 0.41 0.41, 0.91 | 0.64 0.54, 0.73 | 0.32 0.17, 0.47 | 0.41 0.27, 0.57 | 0.44 0.27, 0.59 | 0.46 0.31, 0.60 |
| 7 | 0.55 0.39, 0.68 | 0.51 0.36, 0.65 | 0.50 0.32, 0.65 | 0.61 0.46, 0.75 | 0.48 0.31, 0.64 | 0.49 0.32, 0.64 | | 0.76 0.57, 0.91 | 0.78 0.78, 0.95 | 0.55 0.55, 0.76 | 0.72 0.72, 1.00 | 0.50 0.29, 0.67 | 0.47 0.25, 0.65 | 0.49 0.26, 0.68 | 0.41 0.15, 0.62 | 0.34 0.08, 0.56 |
| 8 | 0.43 0.24, 0.58 | 0.35 0.14, 0.53 | 0.37 0.17, 0.54 | 0.44 0.24, 0.61 | 0.30 0.08, 0.48 | 0.32 0.11, 0.50 | 0.74 0.61, 0.86 | | 0.29 0.29, 0.55 | 0.17 -0.09, 0.44 | 0.33 0.33, 0.84 | 0.29 0.11, 0.46 | 0.33 0.12, 0.51 | 0.33 0.12, 0.53 | 0.24 -0.01, 0.47 | 0.24 0.02, 0.45 |
| 9 | 0.41 0.20, 0.60 | 0.30 0.07, 0.51 | 0.42 0.20, 0.62 | 0.50 0.27, 0.68 | 0.43 0.43, 0.62 | 0.44 0.44, 0.64 | 0.86 0.73, 0.99 | 0.56 0.34, 0.76 | | 0.76 0.76, 0.95 | 0.50 0.50, 1.00 | 0.56 0.35, 0.72 | 0.48 0.26, 0.66 | 0.41 0.16, 0.62 | 0.40 0.40, 0.63 | 0.39 0.34, 0.49 |
| 10 | 0.48 0.27, 0.66 | 0.39 0.15, 0.60 | 0.45 0.22, 0.64 | 0.68 0.48, 0.85 | 0.46 0.23, 0.65 | 0.50 0.26, 0.70 | 0.75 0.59, 0.88 | 0.49 0.26, 0.70 | 0.91 0.91, 1.00 | | 0.71 0.57, 1.00 | 0.59 0.39, 0.76 | 0.45 0.45, 0.64 | 0.43 0.18, 0.64 | 0.52 0.52, 0.73 | 0.54 0.54, 0.74 |

| | | | | | | | | | | | | | | | | |
|---|---|---|---|---|---|---|---|---|---|---|---|---|---|---|---|---|
| 11 | 0.61 0.61, 1.00 | 0.75 0.75, 1.00 | 0.55 0.55, 1.00 | 0.50 0.50, 0.87 | 0.54 0.54, 0.91 | 0.47 0.47, 0.85 | 0.59 0.35, 1.00 | 0.50 0.17, 0.95 | 0.41 NA, 0.97 | 0.63 NA, 1.00 | | 0.65 0.65, 1.00 | 0.54 0.54, 1.00 | 0.54 NA, 1.00 | 0.65 0.65, 1.00 | 0.54 0.54, 1.00 |
| 12 | 0.66 0.54, 0.75 | 0.67 0.56, 0.77 | 0.64 0.50, 0.75 | 0.86 0.78, 0.93 | 0.59 0.44, 0.71 | 0.65 0.50, 0.76 | 0.66 0.52, 0.76 | 0.55 0.38, 0.68 | 0.53 0.34, 0.69 | 0.63 0.44, 0.78 | 0.76 0.49, 1.00 | | 0.91 0.87, 0.94 | 0.95 0.91, 0.98 | 0.95 0.89, 0.99 | 0.92 0.86, 0.96 |
| 13 | 0.35 0.18, 0.50 | 0.41 0.23, 0.57 | 0.34 0.16, 0.50 | 0.68 0.55, 0.79 | 0.35 0.16, 0.52 | 0.39 0.20, 0.55 | 0.56 0.41, 0.68 | 0.48 0.31, 0.62 | 0.46 0.29, 0.62 | 0.55 0.35, 0.71 | 0.67 0.38, 1.00 | 0.94 0.91, 0.96 | | 0.96 0.92, 0.99 | 0.96 0.91, 1.00 | 0.91 0.84, 0.95 |
| 14 | 0.43 0.26, 0.58 | 0.50 0.32, 0.65 | 0.42 0.24, 0.58 | 0.71 0.58, 0.81 | 0.46 0.27, 0.61 | 0.47 0.29, 0.63 | 0.57 0.42, 0.70 | 0.46 0.28, 0.61 | 0.47 0.25, 0.64 | 0.59 0.50, 0.77 | 0.63 0.32, 1.00 | 0.92 0.88, 0.95 | 0.94 0.92, 0.96 | | 0.94 0.86, 1.00 | 0.94 0.87, 1.00 |
| 15 | 0.51 0.32, 0.66 | 0.49 0.29, 0.66 | 0.51 0.31, 0.67 | 0.79 0.65, 0.90 | 0.46 0.42, 0.63 | 0.51 0.51, 0.68 | 0.62 0.43, 0.76 | 0.52 0.32, 0.69 | 0.42 0.17, 0.63 | 0.55 0.55, 0.74 | 0.91 0.58, 1.00 | 0.95 0.89, 1.00 | 0.94 0.88, 0.99 | 0.91 0.83, 0.97 | | 1.00 0.99, 1.00 |
| 16 | 0.48 0.30, 0.64 | 0.46 0.26, 0.63 | 0.48 0.28, 0.64 | 0.77 0.63, 0.89 | 0.44 0.23, 0.61 | 0.47 0.27, 0.64 | 0.60 0.42, 0.74 | 0.58 0.39, 0.74 | 0.41 0.18, 0.61 | 0.53 0.31, 0.72 | 0.86 0.55, 1.00 | 0.91 0.85, 0.96 | 0.91 0.85, 0.96 | 0.88 0.80, 0.94 | 1.00 0.99, NA | |

1=total height, 2=sitting height, 3=knee height, 4=buttock-knee length, 5=foot length left
6=foot length right, 7=head circumference, 8=head breadth, 9=head length 1, 10=head length 2, 11=face height, 13=weight, 14=BMI, 15=arm circumference, 16=waist circumference relaxed, 17=waist circumference sucking

Supplemental Table S9. Shared environmental correlations of anthropometric traits in males (upper diagonal matrix) and females (lower diagonal matrix) with 95% confidence intervals in the initial assessment under additive genetic/ shared environment/ unique environment model.

| | 1 | 2 | 3 | 4 | 5 | 6 | 7 | 8 | 9 | 10 | 11 | 12 | 13 | 14 | 15 | 16 |
|---|---|---|---|---|---|---|---|---|---|---|---|---|---|---|---|---|
| 1 | | 0.82 -1.00, 1.00 | 0.97 -1.00, NA | 0.96 0.96, NA | 0.81 -1.00, 1.00 | 0.71 -1.00, 1.00 | 0.83 -0.91, 1.00 | 1.00 0.97, NA | -0.02 -1.00, 1.00 | 0.11 -1.00, 1.00 | 0.53 -1.00, 1.00 | 0.63 -1.00, 1.00 | 0.16 -1.00, 1.00 | 0.28 -1.00, 1.00 | 0.65 -1.00, 1.00 | 0.64 -1.00, 1.00 |
| 2 | 0.94 -1.00, 1.00 | | 0.65 -1.00, 1.00 | 0.76 -1.00, 1.00 | 0.96 -1.00, NA | 0.52 -1.00, 1.00 | 0.87 -1.00, NA | 1.00 -1.00, NA | -0.15 -1.00, 1.00 | 0.19 -1.00, 1.00 | 0.58 -1.00, 1.00 | 0.37 -1.00, 1.00 | -0.04 -1.00, 1.00 | 0.27 -1.00, 1.00 | 0.51 -1.00, 1.00 | 0.40 -1.00, 1.00 |
| 3 | 0.71 -1.00, 0.91 | 0.80 -1.00, NA | | 0.92 0.92, NA | 0.64 -1.00, 1.00 | 0.61 -1.00, 1.00 | 0.48 -0.41, 1.00 | 1.00 -1.00, NA | -0.33 -1.00, 1.00 | 0.27 -1.00, 1.00 | 0.42 -1.00, 1.00 | 0.56 -1.00, 1.00 | 0.10 -1.00, 1.00 | 0.04 -1.00, 1.00 | 0.67 -1.00, 1.00 | 0.55 -1.00, 1.00 |
| 4 | 1.00 -1.00, NA | 1.00 NA, NA | 0.73 -1.00, 1.00 | | 1.00 NA, NA | 1.00 0.99, NA | 0.49 0.06, 0.92 | 0.91 -1.00, NA | -0.01 -1.00, 1.00 | 0.31 -1.00, 1.00 | 0.64 0.64, 1.00 | 1.00 0.97, NA | 0.87 0.87, 1.00 | 0.84 -1.00, 1.00 | 0.93 0.93, 1.00 | 1.00 1.00, NA |
| 5 | 0.61 -1.00, 0.86 | 0.61 -1.00, 0.94 | 0.56 NA, 1.00 | 0.82 -1.00, 1.00 | | 0.97 NA, NA | 1.00 -1.00, NA | 1.00 -1.00, NA | -0.14 -1.00, 1.00 | 1.00 -1.00, NA | 0.36 -1.00, 1.00 | 1.00 -1.00, NA | 1.00 NA, NA | 1.00 -1.00, NA | 1.00 NA, NA | 1.00 -1.00, NA |
| 6 | 0.49 -1.00, 0.77 | 0.60 -1.00, 1.00 | 0.43 -1.00, 1.00 | 0.74 -1.00, 1.00 | 0.99 -1.00, NA | | 1.00 0.99, NA | 1.00 -1.00, NA | -0.13 -1.00, 1.00 | 0.72 -1.00, 1.00 | 0.73 -1.00, 1.00 | 1.00 -1.00, NA | 1.00 -1.00, NA | 1.00 -1.00, NA | 1.00 0.91, NA | 1.00 -1.00, NA |
| 7 | 1.00 -1.00, NA | 1.00 -1.00, NA | -1.00 NA, 1.00 | 1.00 -1.00, NA | 1.00 -1.00, NA | 1.00 -1.00, NA | | 0.19 -1.00, 1.00 | 0.63 -1.00, 1.00 | 0.69 0.69, 1.00 | 0.23 -1.00, 0.23 | 0.72 -1.00, 1.00 | 0.42 -1.00, 1.00 | 0.47 -1.00, 1.00 | 0.61 0.61, 1.00 | 0.83 NA, NA |
| 8 | -0.19 -1.00, 1.00 | 0.33 -1.00, 1.00 | -0.61 NA, -0.61 | 0.01 -1.00, 1.00 | 0.28 -1.00, 1.00 | 0.17 -1.00, 1.00 | 0.83 -1.00, NA | | 0.13 -1.00, 1.00 | 0.76 -1.00, 1.00 | 0.41 -1.00, 1.00 | 1.00 -1.00, NA | 0.34 -1.00, 1.00 | 0.63 -1.00, NA | 0.95 -1.00, 1.00 | 1.00 -1.00, NA |
| 9 | 0.05 -1.00, 1.00 | 0.57 -1.00, 1.00 | -0.58 -1.00, 1.00 | -0.16 -1.00, 1.00 | 0.07 -1.00, 1.00 | 0.02 -1.00, NA | -1.00 NA, 1.00 | -0.53 -1.00, 1.00 | | 0.55 -1.00, 1.00 | 0.29 NA, 1.00 | -0.43 -1.00, NA | -0.51 -1.00, NA | -0.12 -1.00, 1.00 | 0.11 -1.00, 1.00 | 0.12 -1.00, 1.00 |
| 10 | 0.25 -1.00, 1.00 | 0.63 0.63, 1.00 | 0.03 -1.00, 1.00 | -0.14 -1.00, 1.00 | 0.27 -1.00, 1.00 | 0.29 -1.00, 1.00 | 0.40 -1.00, 1.00 | 0.09 -1.00, 1.00 | 0.64 -1.00, 1.00 | | 0.15 -1.00, 1.00 | 0.41 -1.00, 1.00 | 0.45 -1.00, 1.00 | 0.45 -1.00, 1.00 | 0.42 -1.00, 1.00 | 0.44 -1.00, 1.00 |
| 11 | 0.34 | 0.08 | 0.11 | 0.54 | 0.72 | 0.78 | 1.00 | -0.23 | 0.72 | 0.23 | | 0.21 | -0.04 | 0.21 | 0.06 | 0.19 |

| | | | | | | | | | | | | | | | | |
|---|---|---|---|---|---|---|---|---|---|---|---|---|---|---|---|---|
| | -1.00,<br>1.00 | -1.00,<br>1.00 | -1.00,<br>1.00 | -1.00,<br>1.00 | -1.00,<br>1.00 | -1.00,<br>1.00 | -1.00,<br>NA | -1.00,<br>1.00 | -1.00,<br>1.00 | -1.00,<br>1.00 | | -1.00,<br>1.00 | -1.00,<br>1.00 | -1.00,<br>1.00 | -1.00,<br>NA | -1.00,<br>1.00 |
| 12 | 0.83<br>-1.00,<br>1.00 | 0.96<br>-1.00,<br>NA | 0.65<br>-1.00,<br>1.00 | 0.59<br>-1.00,<br>1.00 | 0.73<br>-1.00,<br>1.00 | 0.68<br>0.68,<br>1.00 | 0.34<br>-1.00,<br>1.00 | -0.17<br>-1.00,<br>NA | -0.77<br>-1.00,<br>1.00 | 0.13<br>-1.00,<br>1.00 | -0.31<br>-1.00,<br>1.00 | | 0.86<br>-1.00,<br>1.00 | 0.59<br>-1.00,<br>1.00 | 1.00<br>NA,<br>NA | 0.90<br>-1.00,<br>1.00 |
| 13 | 0.51<br>-1.00,<br>1.00 | 0.73<br>-1.00,<br>1.00 | 0.46<br>-1.00,<br>1.00 | 0.09<br>-1.00,<br>1.00 | 0.66<br>-1.00,<br>1.00 | 0.64<br>-1.00,<br>1.00 | -0.42<br>-1.00,<br>1.00 | -0.12<br>-1.00,<br>1.00 | -1.00<br>NA,<br>NA | 0.01<br>-1.00,<br>1.00 | -0.71<br>-1.00,<br>1.00 | 0.91<br>-1.00,<br>1.00 | | 0.57<br>0.57,<br>1.00 | 0.86<br>-1.00,<br>1.00 | 0.82<br>-1.00,<br>1.00 |
| 14 | 0.54<br>-1.00,<br>1.00 | 0.62<br>-1.00,<br>1.00 | 0.34<br>-1.00,<br>1.00 | 0.25<br>-1.00,<br>1.00 | 0.36<br>-1.00,<br>1.00 | 0.46<br>-1.00,<br>1.00 | -0.00<br>-1.00,<br>1.00 | -0.09<br>-1.00,<br>1.00 | -0.81<br>NA,<br>1.00 | 0.13<br>-1.00,<br>1.00 | -0.65<br>-1.00,<br>1.00 | 0.88<br>-1.00,<br>NA | 0.98<br>-1.00,<br>NA | | 0.80<br>-1.00,<br>1.00 | 0.52<br>-1.00,<br>1.00 |
| 15 | 0.21<br>-1.00,<br>1.00 | 0.37<br>-1.00,<br>1.00 | 0.10<br>-1.00,<br>1.00 | 0.07<br>-1.00,<br>1.00 | 0.43<br>-1.00,<br>1.00 | 0.32<br>-1.00,<br>1.00 | -0.83<br>-1.00,<br>NA | -0.31<br>-1.00,-<br>0.31 | -0.35<br>-1.00,<br>1.00 | 0.16<br>-1.00,<br>1.00 | -0.76<br>-1.00,<br>NA | 0.58<br>NA,<br>1.00 | 0.77<br>-1.00,<br>1.00 | 0.77<br>-1.00,<br>1.00 | | 0.98<br>0.98,<br>1.00 |
| 16 | 0.29<br>-1.00,<br>1.00 | 0.45<br>-1.00,<br>1.00 | 0.22<br>-1.00,<br>1.00 | 0.20<br>-1.00,<br>1.00 | 0.54<br>-1.00,<br>1.00 | 0.44<br>-1.00,<br>1.00 | -0.03<br>-1.00,<br>1.00 | -0.49<br>-1.00,-<br>0.49 | -0.12<br>-1.00,<br>1.00 | 0.30<br>-1.00,<br>1.00 | -0.71<br>-1.00,-<br>0.71 | 0.59<br>NA,<br>1.00 | 0.70<br>-1.00,<br>1.00 | 0.78<br>-1.00,<br>1.00 | 0.89<br>-1.00,<br>1.00 | |

1=total height, 2=sitting height, 3=knee height, 4=buttock-knee length, 5=foot length left
6=foot length right, 7=head circumference, 8=head breadth, 9=head length 1, 10=head length 2, 11=face height, 13=weight, 14=BMI, 15=arm circumference, 16=waist circumference relaxed, 17=waist circumference sucking

Supplemental Table S10. Unique environmental correlations of anthropometric traits in males (upper diagonal matrix) and females (lower diagonal matrix) with 95% confidence intervals in the initial assessment under the additive genetic / unique environment model.

| | 1 | 2 | 3 | 4 | 5 | 6 | 7 | 8 | 9 | 10 | 11 | 12 | 13 | 14 | 15 | 16 |
|---|---|---|---|---|---|---|---|---|---|---|---|---|---|---|---|---|
| 1 | | 0.69<br>0.62,<br>0.74 | 0.67<br>0.60,<br>0.73 | 0.48<br>0.38,<br>0.56 | 0.60<br>0.51,<br>0.67 | 0.39<br>0.28,<br>0.49 | 0.48<br>0.38,<br>0.57 | 0.33<br>0.21,<br>0.43 | 0.38<br>0.27,<br>0.48 | 0.27<br>0.15,<br>0.38 | 0.22<br>0.10,<br>0.33 | 0.68<br>0.61,<br>0.74 | 0.37<br>0.26,<br>0.47 | 0.39<br>0.28,<br>0.49 | 0.43<br>0.33,<br>0.52 | 0.37<br>0.26,<br>0.47 |
| 2 | 0.69<br>0.62,<br>0.75 | | 0.43<br>0.32,<br>0.52 | 0.19<br>0.07,<br>0.30 | 0.43<br>0.33,<br>0.52 | 0.22<br>0.10,<br>0.33 | 0.28<br>0.17,<br>0.39 | 0.25<br>0.13,<br>0.36 | 0.24<br>0.13,<br>0.35 | 0.20<br>0.08,<br>0.31 | 0.17<br>0.06,<br>0.28 | 0.48<br>0.38,<br>0.57 | 0.27<br>0.15,<br>0.38 | 0.33<br>0.21,<br>0.43 | 0.31<br>0.20,<br>0.42 | 0.32<br>0.21,<br>0.43 |
| 3 | 0.50<br>0.40,<br>0.59 | 0.35<br>0.24,<br>0.46 | | 0.31<br>0.20,<br>0.42 | 0.54<br>0.45,<br>0.62 | 0.44<br>0.34,<br>0.54 | 0.44<br>0.33,<br>0.53 | 0.36<br>0.24,<br>0.46 | 0.25<br>0.13,<br>0.36 | 0.23<br>0.11,<br>0.34 | 0.17<br>0.06,<br>0.29 | 0.57<br>0.48,<br>0.65 | 0.39<br>0.28,<br>0.49 | 0.37<br>0.26,<br>0.47 | 0.43<br>0.33,<br>0.53 | 0.36<br>0.25,<br>0.46 |
| 4 | 0.49<br>0.39,<br>0.58 | 0.20<br>0.08,<br>0.32 | 0.30<br>0.19,<br>0.41 | | 0.35<br>0.25,<br>0.45 | 0.29<br>0.17,<br>0.39 | 0.37<br>0.26,<br>0.46 | 0.15<br>0.03,<br>0.26 | 0.31<br>0.20,<br>0.41 | 0.23<br>0.11,<br>0.34 | 0.24<br>0.13,<br>0.35 | 0.56<br>0.47,<br>0.64 | 0.48<br>0.38,<br>0.57 | 0.32<br>0.21,<br>0.43 | 0.38<br>0.28,<br>0.48 | 0.39<br>0.28,<br>0.49 |
| 5 | 0.61<br>0.53,<br>0.69 | 0.53<br>0.43,<br>0.61 | 0.46<br>0.35,<br>0.55 | 0.31<br>0.19,<br>0.42 | | 0.54<br>0.46,<br>0.62 | 0.36<br>0.25,<br>0.46 | 0.25<br>0.13,<br>0.36 | 0.27<br>0.15,<br>0.38 | 0.28<br>0.16,<br>0.38 | 0.13<br>0.01,<br>0.24 | 0.54<br>0.45,<br>0.63 | 0.39<br>0.28,<br>0.49 | 0.39<br>0.28,<br>0.49 | 0.33<br>0.22,<br>0.44 | 0.28<br>0.16,<br>0.39 |
| 6 | 0.53<br>0.43,<br>0.61 | 0.40<br>0.28,<br>0.50 | 0.41<br>0.30,<br>0.50 | 0.35<br>0.24,<br>0.46 | 0.59<br>0.51,<br>0.67 | | 0.25<br>0.13,<br>0.36 | 0.19<br>0.07,<br>0.31 | 0.20<br>0.08,<br>0.31 | 0.27<br>0.15,<br>0.38 | 0.04<br>-0.08,<br>0.16 | 0.47<br>0.37,<br>0.56 | 0.41<br>0.30,<br>0.51 | 0.36<br>0.25,<br>0.46 | 0.29<br>0.18,<br>0.40 | 0.29<br>0.17,<br>0.40 |
| 7 | 0.30<br>0.18,<br>0.42 | 0.25<br>0.12,<br>0.36 | 0.25<br>0.12,<br>0.36 | 0.21<br>0.09,<br>0.33 | 0.33<br>0.21,<br>0.44 | 0.41<br>0.30,<br>0.51 | | 0.39<br>0.28,<br>0.49 | 0.41<br>0.30,<br>0.50 | 0.34<br>0.23,<br>0.44 | 0.15<br>0.03,<br>0.26 | 0.53<br>0.43,<br>0.61 | 0.43<br>0.32,<br>0.52 | 0.31<br>0.19,<br>0.41 | 0.41<br>0.31,<br>0.51 | 0.39<br>0.29,<br>0.49 |
| 8 | 0.19<br>0.07,<br>0.31 | 0.14<br>0.02,<br>0.27 | 0.15<br>0.02,<br>0.27 | 0.12<br>-0.01,<br>0.24 | 0.23<br>0.10,<br>0.35 | 0.20<br>0.07,<br>0.32 | 0.18<br>0.05,<br>0.30 | | 0.08<br>-0.05,<br>0.20 | 0.17<br>0.05,<br>0.29 | 0.12<br>0.00,<br>0.24 | 0.43<br>0.33,<br>0.53 | 0.39<br>0.28,<br>0.49 | 0.26<br>0.14,<br>0.37 | 0.34<br>0.22,<br>0.44 | 0.31<br>0.19,<br>0.41 |
| 9 | 0.19<br>0.07,<br>0.31 | 0.26<br>0.13,<br>0.37 | 0.12<br>-0.00,<br>0.25 | 0.15<br>0.02,<br>0.27 | 0.29<br>0.17,<br>0.40 | 0.29<br>0.17,<br>0.40 | 0.32<br>0.20,<br>0.42 | -0.01<br>-0.13,<br>0.12 | | 0.31<br>0.20,<br>0.41 | 0.07<br>-0.05,<br>0.19 | 0.38<br>0.27,<br>0.48 | 0.29<br>0.18,<br>0.40 | 0.26<br>0.14,<br>0.37 | 0.32<br>0.20,<br>0.42 | 0.28<br>0.16,<br>0.39 |
| 10 | 0.26<br>0.14,<br>0.37 | 0.18<br>0.06,<br>0.30 | 0.26<br>0.14,<br>0.38 | 0.18<br>0.06,<br>0.30 | 0.31<br>0.19,<br>0.42 | 0.23<br>0.11,<br>0.35 | 0.40<br>0.29,<br>0.50 | 0.07<br>-0.06,<br>0.19 | 0.35<br>0.24,<br>0.45 | | -0.01<br>-0.12,<br>0.11 | 0.33<br>0.22,<br>0.44 | 0.29<br>0.17,<br>0.40 | 0.22<br>0.11,<br>0.34 | 0.30<br>0.19,<br>0.41 | 0.24<br>0.12,<br>0.35 |

| | | | | | | | | | | | | | | | | |
|---|---|---|---|---|---|---|---|---|---|---|---|---|---|---|---|---|
| 11 | 0.08<br>-0.04,<br>0.20 | 0.11<br>-0.01,<br>0.23 | -0.03<br>-0.15,<br>0.09 | 0.07<br>-0.06,<br>0.19 | -0.00<br>-0.13,<br>0.12 | 0.11<br>-0.02,<br>0.23 | 0.11<br>-0.02,<br>0.23 | 0.07<br>-0.06,<br>0.19 | -0.09<br>-0.21,<br>0.03 | 0.01<br>-0.11,<br>0.13 | | 0.25<br>0.14,<br>0.36 | 0.22<br>0.10,<br>0.33 | 0.20<br>0.09,<br>0.31 | 0.21<br>0.10,<br>0.32 | 0.18<br>0.07,<br>0.29 |
| 12 | 0.63<br>0.55,<br>0.70 | 0.54<br>0.44,<br>0.62 | 0.43<br>0.32,<br>0.53 | 0.52<br>0.42,<br>0.61 | 0.53<br>0.44,<br>0.62 | 0.48<br>0.37,<br>0.57 | 0.45<br>0.35,<br>0.55 | 0.31<br>0.19,<br>0.42 | 0.27<br>0.15,<br>0.39 | 0.35<br>0.24,<br>0.46 | 0.10<br>-0.02,<br>0.22 | | 0.93<br>0.91,<br>0.95 | 0.74<br>0.69,<br>0.79 | 0.74<br>0.68,<br>0.79 | 0.70<br>0.63,<br>0.76 |
| 13 | 0.32<br>0.20,<br>0.43 | 0.34<br>0.23,<br>0.45 | 0.30<br>0.18,<br>0.42 | 0.41<br>0.30,<br>0.51 | 0.38<br>0.26,<br>0.48 | 0.34<br>0.23,<br>0.45 | 0.42<br>0.31,<br>0.52 | 0.29<br>0.17,<br>0.40 | 0.24<br>0.12,<br>0.36 | 0.31<br>0.19,<br>0.42 | 0.09<br>-0.04,<br>0.21 | 0.94<br>0.92,<br>0.95 | | 0.75<br>0.70,<br>0.80 | 0.73<br>0.67,<br>0.78 | 0.70<br>0.64,<br>0.76 |
| 14 | 0.24<br>0.12,<br>0.36 | 0.25<br>0.12,<br>0.36 | 0.21<br>0.09,<br>0.33 | 0.37<br>0.25,<br>0.47 | 0.28<br>0.16,<br>0.40 | 0.27<br>0.15,<br>0.38 | 0.27<br>0.15,<br>0.39 | 0.25<br>0.13,<br>0.37 | 0.11<br>-0.02,<br>0.23 | 0.21<br>0.09,<br>0.33 | 0.08<br>-0.04,<br>0.20 | 0.77<br>0.71,<br>0.82 | 0.83<br>0.78,<br>0.86 | | 0.60<br>0.52,<br>0.68 | 0.57<br>0.49,<br>0.65 |
| 15 | 0.22<br>0.10,<br>0.34 | 0.20<br>0.07,<br>0.31 | 0.23<br>0.11,<br>0.35 | 0.29<br>0.17,<br>0.40 | 0.29<br>0.17,<br>0.40 | 0.25<br>0.13,<br>0.37 | 0.25<br>0.13,<br>0.37 | 0.18<br>0.06,<br>0.30 | 0.18<br>0.06,<br>0.30 | 0.30<br>0.18,<br>0.41 | -0.00<br>-0.12,<br>0.12 | 0.64<br>0.56,<br>0.71 | 0.67<br>0.60,<br>0.74 | 0.57<br>0.48,<br>0.65 | | 0.83<br>0.79,<br>0.87 |
| 16 | 0.24<br>0.11,<br>0.35 | 0.21<br>0.09,<br>0.33 | 0.24<br>0.12,<br>0.35 | 0.26<br>0.14,<br>0.37 | 0.28<br>0.16,<br>0.39 | 0.29<br>0.17,<br>0.40 | 0.28<br>0.16,<br>0.40 | 0.14<br>0.01,<br>0.26 | 0.19<br>0.07,<br>0.31 | 0.31<br>0.19,<br>0.42 | 0.07<br>-0.05,<br>0.19 | 0.63<br>0.55,<br>0.70 | 0.66<br>0.58,<br>0.72 | 0.54<br>0.45,<br>0.63 | 0.87<br>0.84,<br>0.90 | |

1=total height, 2=sitting height, 3=knee height, 4=buttock-knee length, 5=foot length left
6=foot length right, 7=head circumference, 8=head breadth, 9=head length 1, 10=head length 2, 11=face height, 13=weight, 14=BMI, 15=arm circumference, 16=waist circumference relaxed, 17=waist circumference sucking

Supplemental Table S11. Explorative factor analysis of anthropometric traits in the follow-up assessment in males and females.[1]

| | Males | | | Females | | |
|---|---|---|---|---|---|---|
| | 1. factor | 2. factor | 3. factor | 1. factor | 2. factor | 3. factor |
| Eigenvalue | 9.55 | 2.74 | 1.36 | 8.10 | 3.24 | 1.36 |
| % of explained variation | 61 | 18 | 9 | 56 | 23 | 10 |
| Factor loadings | | | | | | |
| Height | 0.155 | **0.915** | 0.183 | 0.026 | **0.961** | 0.106 |
| Sitting height | 0.262 | **0.682** | 0.315 | 0.123 | **0.739** | 0.262 |
| Knee height | 0.126 | **0.836** | 0.092 | 0.159 | **0.807** | -0.002 |
| Buttock-knee length | 0.353 | **0.721** | 0.163 | 0.433 | **0.690** | -0.018 |
| Foot length left | 0.180 | **0.849** | 0.131 | 0.180 | **0.864** | 0.138 |
| Foot length right | 0.177 | **0.856** | 0.132 | 0.189 | **0.865** | 0.108 |
| Head circumference | 0.304 | 0.298 | **0.731** | 0.263 | 0.235 | **0.730** |
| Head breadth | 0.261 | 0.194 | 0.428 | 0.300 | 0.034 | 0.426 |
| Head length 1 | 0.125 | 0.167 | **0.801** | 0.133 | 0.180 | **0.665** |
| Head length 2 | 0.309 | 0.279 | **0.672** | 0.196 | 0.222 | **0.586** |
| Face height | 0.296 | 0.361 | 0.360 | 0.126 | 0.318 | 0.361 |
| Shoulder breadth | 0.238 | 0.422 | 0.219 | 0.290 | 0.268 | 0.077 |
| Wrist breadth | 0.277 | **0.527** | 0.202 | 0.366 | 0.406 | 0.087 |
| weight | **0.789** | 0.500 | 0.284 | **0.897** | 0.398 | 0.108 |
| BMI | **0.903** | 0.100 | 0.250 | **0.976** | 0.004 | 0.072 |
| Arm circumference | **0.821** | 0.211 | 0.275 | **0.896** | 0.105 | 0.110 |
| Hip breadth | **0.661** | 0.395 | 0.072 | **0.758** | 0.252 | 0.025 |
| Waist circ. relaxed | **0.873** | 0.262 | 0.114 | **0.892** | 0.154 | 0.135 |
| Waist circ. sucking | **0.840** | 0.267 | 0.140 | **0.887** | 0.121 | 0.152 |
| Biceps skinfold | **0.704** | -0.079 | -0.134 | 0.483 | -0.037 | -0.050 |
| Triceps skinfold | **0.667** | -0.054 | -0.072 | **0.580** | 0.009 | -0.075 |

[1]Varimax rotation is used. The key factor loadings for each factor are bolded.

Supplemental Table S12. Correlations of anthropometric traits in males (upper diagonal matrix) and females (lower diagonal matrix) with 95% confidence intervals in the follow-up assessment.

| | 1 | 2 | 3 | 4 | 5 | 6 | 7 | 8 | 9 | 10 | 11 | 12 | 13 | 14 | 15 | 16 | 17 | 18 | 19 | 20 | 21 |
|---|---|---|---|---|---|---|---|---|---|---|---|---|---|---|---|---|---|---|---|---|---|
| 1 | | .81 .78, .84 | .84 .81, .86 | .79 .76, .82 | .71 .67, .75 | .73 .69, .76 | .47 .40, .53 | .31 .24, .38 | .29 .22, .36 | .42 .36, .48 | .45 .39, .51 | .46 .40, .52 | .51 .45, .57 | .63 .58, .67 | .22 .14, .29 | .36 .29, .43 | .47 .40, .53 | .38 .31, .45 | .40 .34, .47 | .06 -.01, .14 | .10 .02, .17 |
| 2 | .82 .79, .85 | | .58 .52, .63 | .57 .51, .62 | .58 .53, .63 | .59 .53, .64 | .46 .39, .52 | .33 .26, .40 | .32 .25, .39 | .50 .44, .56 | .48 .42, .54 | .51 .45, .57 | .51 .45, .57 | .67 .63, .71 | .38 .31, .45 | .49 .43, .55 | .51 .45, .57 | .38 .31, .45 | .40 .34, .47 | .06 -.02, .14 | .10 .02, .17 |
| 3 | .82 .79, .85 | .61 .55, .66 | | .76 .73, .79 | .70 .66, .74 | .71 .67, .75 | .37 .30, .44 | .23 .16, .31 | .25 .18, .32 | .35 .28, .42 | .38 .31, .44 | .38 .31, .45 | .45 .39, .51 | .55 .49, .60 | .20 .13, .28 | .28 .20, .35 | .38 .31, .44 | .36 .29, .43 | .37 .30, .44 | .03 -.04, .11 | .08 .00, .16 |
| 4 | .72 .68, .76 | .51 .45, .58 | .69 .64, .73 | | .65 .60, .69 | .64 .59, .68 | .47 .40, .52 | .28 .21, .35 | .32 .25, .39 | .45 .39, .51 | .44 .37, .50 | .41 .35, .48 | .45 .39, .51 | .68 .64, .72 | .40 .33, .46 | .46 .40, .52 | .49 .43, .55 | .54 .48, .60 | .56 .50, .61 | .20 .12, .27 | .22 .15, .30 |
| 5 | .74 .69, .78 | .65 .59, .70 | .71 .66, .75 | .64 .58, .69 | | .95 .94, .96 | .40 .34, .47 | .25 .17, .32 | .31 .24, .38 | .39 .32, .45 | .40 .33, .46 | .38 .31, .45 | .56 .50, .61 | .59 .53, .63 | .32 .25, .39 | .38 .31, .44 | .45 .39, .51 | .40 .33, .46 | .39 .32, .45 | .06 -.02, .14 | .06 -.01, .14 |
| 6 | .74 .69, .77 | .64 .58, .69 | .70 .66, .75 | .64 .58, .69 | .96 .95, .96 | | .40 .33, .46 | .26 .18, .33 | .31 .24, .38 | .40 .33, .46 | .40 .33, .46 | .41 .34, .47 | .56 .51, .61 | .59 .53, .64 | .32 .24, .39 | .37 .30, .44 | .46 .40, .52 | .38 .31, .45 | .38 .31, .45 | .09 .01, .17 | .09 .01, .16 |
| 7 | .34 .26, .42 | .40 .33, .48 | .29 .21, .37 | .33 .25, .41 | .38 .30, .45 | .37 .29, .44 | | .61 .56, .66 | .70 .66, .74 | .59 .54, .64 | .47 .40, .53 | .34 .27, .41 | .36 .29, .43 | .57 .51, .62 | .44 .38, .50 | .47 .41, .53 | .41 .34, .47 | .44 .38, .50 | .44 .38, .50 | .14 .06, .22 | .17 .09, .24 |
| 8 | .10 .02, .19 | .20 .11, .28 | .08 -.00 .17 | .13 .04, .21 | .16 .07, .24 | .17 .08, .25 | .55 .48, .61 | | .30 .22, .37 | .29 .21, .36 | .35 .28, .42 | .22 .15, .30 | .28 .21, .35 | .43 .37, .50 | .36 .29, .43 | .36 .29, .43 | .30 .22, .37 | .30 .23, .37 | .34 .26, .40 | .11 .03, .18 | .13 .05, .21 |
| 9 | .28 .20, .36 | .31 .23, .39 | .20 .11, .28 | .31 .23, .39 | .31 .23, .39 | .27 .18, .35 | .59 .53, .65 | .24 .16, .32 | | .73 .70, .77 | .34 .27, .41 | .24 .16, .31 | .24 .17, .31 | .37 .30, .44 | .30 .23, .37 | .32 .25, .39 | .18 .10, .25 | .24 .17, .31 | .25 .18, .33 | .05 -.03, .13 | .10 .02, .17 |
| 10 | .32 .24, .40 | .40 .32, .47 | .28 .20, .36 | .28 .20, .36 | .30 .22, .38 | .29 .20, .37 | .53 .46, .59 | .25 .17, .33 | .60 .54, .65 | | .46 .39, .52 | .33 .26, .40 | .36 .29, .43 | .56 .50, .61 | .46 .40, .52 | .50 .44, .56 | .32 .25, .39 | .41 .34, .47 | .44 .38, .50 | .17 .10, .25 | .22 .14, .29 |

| | | | | | | | | | | | | | | | | | | | | | |
|---|---|---|---|---|---|---|---|---|---|---|---|---|---|---|---|---|---|---|---|---|---|
| 11 | .37<br>.30,<br>.45 | .38<br>.30,<br>.45 | .31<br>.23,<br>.39 | .30<br>.21,<br>.37 | .36<br>.28,<br>.44 | .35<br>.27,<br>.43 | .41<br>.33,<br>.48 | .18<br>.09,<br>.26 | .30<br>.21,<br>.37 | .28<br>.19,<br>.35 | | .31<br>.24,<br>.38 | .36<br>.30,<br>.43 | .50<br>.44,<br>.56 | .37<br>.30,<br>.44 | .42<br>.35,<br>.48 | .38<br>.31,<br>.44 | .36<br>.29,<br>.43 | .40<br>.33,<br>.46 | .14<br>.06,<br>.22 | .16<br>.08,<br>.24 |
| 12 | .27<br>.18,<br>.35 | .28<br>.19,<br>.36 | .35<br>.27,<br>.42 | .25<br>.17,<br>.33 | .32<br>.23,<br>.39 | .32<br>.24,<br>.40 | .30<br>.21,<br>.37 | .17<br>.09,<br>.26 | .10<br>.01,<br>.18 | .24<br>.16,<br>.32 | .19<br>.10,<br>.27 | | .42<br>.35,<br>.48 | .48<br>.41,<br>.53 | .34<br>.26,<br>.40 | .37<br>.30,<br>.44 | .43<br>.37,<br>.49 | .30<br>.22,<br>.37 | .31<br>.24,<br>.38 | .06<br>-.02,<br>.14 | .11<br>.03,<br>.19 |
| 13 | .41<br>.34,<br>.48 | .40<br>.32,<br>.47 | .36<br>.28,<br>.43 | .45<br>.38,<br>.52 | .44<br>.36,<br>.51 | .45<br>.38,<br>.52 | .26<br>.18,<br>.34 | .25<br>.16,<br>.33 | .21<br>.12,<br>.29 | .24<br>.16,<br>.32 | .19<br>.11,<br>.28 | .22<br>.13,<br>.30 | | .54<br>.48,<br>.59 | .39<br>.32,<br>.45 | .46<br>.40,<br>.52 | .49<br>.42,<br>.54 | .34<br>.26,<br>.40 | .36<br>.29,<br>.43 | .10<br>.02,<br>.18 | .12<br>.04,<br>.19 |
| 14 | .42<br>.35,<br>.49 | .47<br>.39,<br>.53 | .45<br>.38,<br>.52 | .68<br>.63,<br>.73 | .50<br>.43,<br>.57 | .50<br>.44,<br>.57 | .46<br>.39,<br>.53 | .36<br>.28,<br>.43 | .33<br>.24,<br>.40 | .39<br>.32,<br>.46 | .27<br>.19,<br>.35 | .46<br>.39,<br>.53 | .55<br>.48,<br>.61 | | .90<br>.88,<br>.91 | .87<br>.85,<br>.89 | .74<br>.71,<br>.78 | .85<br>.83,<br>.87 | .82<br>.80,<br>.85 | .41<br>.34,<br>.47 | .41<br>.34,<br>.47 |
| 15 | .05<br>-.04<br>.13 | .17<br>.09,<br>.26 | .16<br>.07,<br>.24 | .45<br>.38,<br>.52 | .24<br>.16,<br>.33 | .25<br>.16,<br>.33 | .37<br>.29,<br>.44 | .35<br>.27,<br>.42 | .24<br>.16,<br>.33 | .30<br>.22,<br>.38 | .14<br>.06,<br>.23 | .40<br>.32,<br>.47 | .43<br>.36,<br>.50 | .93<br>.91,<br>.94 | | .90<br>.88,<br>.91 | .67<br>.63,<br>.71 | .85<br>.83,<br>.87 | .80<br>.77,<br>.83 | .48<br>.42,<br>.54 | .46<br>.40,<br>.52 |
| 16 | .18<br>.09,<br>.26 | .24<br>.16,<br>.33 | .24<br>.15,<br>.32 | .50<br>.43,<br>.57 | .26<br>.17,<br>.34 | .26<br>.17,<br>.34 | .36<br>.28,<br>.44 | .32<br>.23,<br>.40 | .26<br>.17,<br>.34 | .27<br>.19,<br>.35 | .18<br>.09,<br>.27 | .31<br>.23,<br>.39 | .39<br>.32,<br>.47 | .87<br>.85,<br>.89 | .90<br>.88,<br>.92 | | .64<br>.59,<br>.69 | .78<br>.75,<br>.81 | .76<br>.73,<br>.79 | .46<br>.40,<br>.52 | .45<br>.39,<br>.51 |
| 17 | .28<br>.19,<br>.35 | .33<br>.25,<br>.41 | .33<br>.25,<br>.41 | .57<br>.50,<br>.62 | .38<br>.30,<br>.45 | .38<br>.30,<br>.45 | .33<br>.25,<br>.40 | .34<br>.26,<br>.42 | .23<br>.15,<br>.31 | .26<br>.18,<br>.34 | .13<br>.05,<br>.22 | .36<br>.28,<br>.43 | .40<br>.32,<br>.47 | .84<br>.81,<br>.86 | .81<br>.77,<br>.84 | .74<br>.70,<br>.78 | | .67<br>.63,<br>.71 | .63<br>.58,<br>.67 | .44<br>.37,<br>.50 | .41<br>.34,<br>.47 |
| 18 | .20<br>.12,<br>.29 | .23<br>.14,<br>.31 | .25<br>.17,<br>.34 | .51<br>.45,<br>.58 | .34<br>.26,<br>.42 | .33<br>.25,<br>.41 | .40<br>.32,<br>.47 | .29<br>.21,<br>.37 | .25<br>.16,<br>.33 | .28<br>.19,<br>.36 | .24<br>.15,<br>.32 | .34<br>.26,<br>.42 | .41<br>.33,<br>.48 | .86<br>.84,<br>.89 | .86<br>.83,<br>.88 | .81<br>.77,<br>.84 | .70<br>.66,<br>.75 | | .95<br>.94,<br>.95 | .49<br>.43,<br>.55 | .45<br>.38,<br>.51 |
| 19 | .18<br>.09,<br>.26 | .21<br>.12,<br>.29 | .21<br>.13,<br>.30 | .50<br>.43,<br>.57 | .32<br>.23,<br>.39 | .31<br>.23,<br>.39 | .38<br>.31,<br>.46 | .33<br>.25,<br>.41 | .26<br>.18,<br>.34 | .28<br>.19,<br>.36 | .21<br>.12,<br>.30 | .34<br>.26,<br>.41 | .41<br>.33,<br>.48 | .85<br>.82,<br>.87 | .86<br>.83,<br>.88 | .81<br>.78,<br>.84 | .71<br>.67,<br>.76 | .92<br>.91,<br>.93 | | .48<br>.41,<br>.54 | .44<br>.37,<br>.50 |
| 20 | -.02<br>.11,<br>.07 | .07<br>.02,<br>.16 | .03<br>.06,<br>.12 | .13<br>.04,<br>.21 | .07<br>.02,<br>.16 | .08<br>-02,<br>.16 | .11<br>.02,<br>.20 | .10<br>.01,<br>.19 | .06<br>-.03,<br>.15 | .10<br>.01,<br>.19 | .05<br>-.04,<br>.14 | .11<br>.01,<br>.19 | .05<br>-.04,<br>.14 | .34<br>.25,<br>.41 | .39<br>.31,<br>.46 | .35<br>.26,<br>.42 | .19<br>.10,<br>.27 | .37<br>.29,<br>.45 | .34<br>.26,<br>.42 | | .78<br>.75,<br>.81 |
| 21 | -.00<br>.09,<br>.09 | .08<br>-.01<br>.17 | .06<br>-.03<br>.15 | .14<br>.05,<br>.23 | .08<br>-.01<br>.17 | .09<br>.00,<br>.18 | .07<br>-.02<br>.16 | .11<br>.02,<br>.20 | -.01<br>-.10,<br>.08 | .09<br>-.00,<br>.18 | .05<br>-.04,<br>.14 | .06<br>-.03,<br>.15 | .05<br>-.04,<br>.14 | .34<br>.26,<br>.42 | .39<br>.31,<br>.46 | .40<br>.32,<br>.47 | .28<br>.20,<br>.36 | .35<br>.27,<br>.42 | .31<br>.23,<br>.39 | .65<br>.59,<br>.70 | |

1=total height, 2=sitting height, 3=knee height, 4=buttock-knee length, 5=foot length left, 6=foot length right, 7=head circumference, 8=head breadth, 9=head length 1, 10=head length 2, 11=face height, 12=shoulder breadth, 13=wrist breadth, 14=weight, 15=BMI, 16=arm circumference, 17=hip breadth, 18=waist circumference relaxed, 19=waist circumference sucking, 20=biceps skinfold, 21=triceps skinfold

Supplemental Table S13. Additive genetic correlations of anthropometric traits in males (upper diagonal matrix) and females (lower diagonal matrix) with 95% confidence intervals in the follow-up assessment under the additive genetic / unique environment model.

| | 1 | 2 | 3 | 4 | 5 | 6 | 7 | 8 | 9 | 10 | 11 | 12 | 13 | 14 | 15 | 16 | 17 | 18 | 19 | 20 | 21 |
|---|---|---|---|---|---|---|---|---|---|---|---|---|---|---|---|---|---|---|---|---|---|
| 1 | | .85 .82, .88 | .89 .86, .92 | .86 .82, .89 | .78 .73, .83 | .79 .74, .83 | .47 .38, .55 | .32 .22, .42 | .30 .19, .40 | .42 .32, .51 | .56 .46, .64 | .50 .42, .58 | .56 .47, .63 | .63 .56, .69 | .24 .13, .34 | .34 .24, .43 | .48 .39, .56 | .39 .29, .48 | .42 .33, .51 | .08 -.03, .18 | .12 .01, .22 |
| 2 | .84 .80, .88 | | .61 .54, .68 | .63 .55, .70 | .64 .56, .71 | .66 .59, .72 | .50 .41, .59 | .39 .28, .48 | .35 .25, .45 | .53 .44, .61 | .61 .52, .70 | .57 .49, .65 | .56 .47, .64 | .70 .63, .75 | .39 .29, .48 | .49 .40, .57 | .53 .45, .61 | .40 .30, .49 | .44 .34, .53 | .08 -.04, .19 | .13 .01, .24 |
| 3 | .90 .86, .93 | .70 .62, .77 | | .86 .81, .89 | .76 .71, .81 | .78 .73, .82 | .38 .28, .47 | .23 .12, .33 | .26 .15, .36 | .37 .27, .47 | .45 .34, .54 | .43 .34, .52 | .47 .38, .56 | .56 .48, .63 | .20 .09, .30 | .27 .17, .37 | .40 .30, .48 | .37 .27, .47 | .39 .29, .49 | .04 -.07, .15 | .09 -.02, .20 |
| 4 | .78 .72, .82 | .58 .49, .67 | .81 .75, .86 | | .72 .65, .78 | .70 .63, .76 | .50 .41, .59 | .28 .17, .38 | .35 .25, .45 | .47 .38, .56 | .57 .47, .67 | .45 .35, .53 | .50 .40, .59 | .70 .64, .76 | .40 .30, .49 | .46 .37, .54 | .53 .44, .60 | .57 .49, .65 | .59 .50, .66 | .20 .09, .31 | .25 .14, .35 |
| 5 | .76 .71, .81 | .69 .62, .76 | .78 .72, .83 | .69 .61, .75 | | .99 .98, 1.00 | .44 .34, .53 | .29 .18, .39 | .33 .22, .43 | .41 .31, .50 | .51 .40, .60 | .45 .35, .54 | .63 .55, .70 | .62 .55, .69 | .33 .23, .43 | .39 .29, .48 | .48 .39, .57 | .43 .33, .53 | .44 .33, .53 | .06 -.05, .17 | .08 -.03, .19 |
| 6 | .77 .71, .81 | .69 .62, .75 | .78 .72, .83 | .69 .62, .76 | 1.0 .99, 1.0 | | .42 .32, .51 | .30 .19, .40 | .33 .22, .43 | .44 .34, .53 | .54 .43, .63 | .47 .38, .56 | .65 .57, .72 | .62 .54, .68 | .32 .22, .42 | .37 .27, .46 | .50 .41, .58 | .42 .32, .51 | .44 .33, .53 | .08 -.03, .19 | .10 -.01, .21 |
| 7 | .34 .23, .45 | .43 .32, .53 | .33 .21, .45 | .34 .22, .45 | .39 .28, .49 | .39 .28, .49 | | .65 .57, .71 | .75 .70, .80 | .65 .57, .71 | .59 .50, .68 | .40 .30, .49 | .41 .30, .50 | .58 .51, .65 | .47 .37, .55 | .48 .39, .56 | .44 .34, .52 | .47 .37, .56 | .49 .39, .57 | .14 .03, .25 | .17 .05, .27 |
| 8 | .09 .03, .21 | .19 .06, .31 | .10 .03, .23 | .12 .01, .25 | .16 .04, .28 | .17 .05, .29 | .60 .50, .68 | | .32 .21, .42 | .33 .23, .44 | .45 .33, .55 | .23 .12, .34 | .32 .21, .43 | .44 .35, .52 | .37 .27, .46 | .39 .29, .48 | .29 .19, .39 | .30 .19, .41 | .35 .24, .45 | .09 -.03, .20 | .13 .01, .24 |
| 9 | .29 .17, .40 | .31 .18, .43 | .26 .12, .39 | .33 .20, .45 | .32 .19, .44 | .27 .14, .39 | .69 .60, .77 | .28 .14, .41 | | .80 .75, .85 | .51 .39, .61 | .28 .17, .38 | .30 .18, .40 | .38 .28, .47 | .31 .20, .41 | .32 .22, .42 | .19 .08, .29 | .25 .13, .35 | .27 .16, .38 | .04 -.08, .16 | .10 -.01, .22 |
| 10 | .39 .27, .50 | .48 .36, .59 | .36 .23, .49 | .33 .19, .45 | .39 .26, .50 | .36 .24, .48 | .61 .51, .70 | .33 .20, .46 | .72 .62, .81 | | .60 .49, .69 | .37 .27, .47 | .41 .31, .51 | .57 .49, .64 | .47 .38, .56 | .49 .40, .57 | .33 .23, .43 | .42 .32, .52 | .46 .36, .55 | .18 .06, .29 | .23 .12, .34 |
| 11 | .43 .31, .54 | .48 .35, .60 | .41 .27, .53 | .38 .25, .50 | .42 .29, .53 | .42 .29, .53 | .49 .37, .60 | .26 .12, .39 | .45 .31, .57 | .43 .28, .56 | | .41 .29, .51 | .45 .34, .56 | .63 .54, .71 | .47 .36, .57 | .54 .44, .64 | .48 .38, .57 | .45 .34, .55 | .51 .40, .61 | .19 .06, .31 | .21 .08, .33 |
| 12 | .26 .15, .37 | .28 .16, .39 | .39 .28, .50 | .24 .12, .35 | .32 .21, .42 | .33 .22, .44 | .29 .18, .40 | .15 .02, .26 | .08 -.06, .21 | .28 .15, .40 | .24 .11, .37 | | .49 .39, .58 | .51 .43, .59 | .36 .26, .46 | .41 .31, .50 | .47 .38, .56 | .31 .20, .41 | .35 .24, .45 | .08- .03, .19 | .15 .04, .26 |

| | | | | | | | | | | | | | | | | | | | | | |
|---|---|---|---|---|---|---|---|---|---|---|---|---|---|---|---|---|---|---|---|---|---|
| 13 | .43<br>.32,<br>.53 | .43<br>.32,<br>.53 | .41<br>.29,<br>.51 | .52<br>.41,<br>.61 | .49<br>.38,<br>.58 | .50<br>.39,<br>.59 | .31<br>.18,<br>.42 | .28<br>.15,<br>.39 | .20<br>.06,<br>.33 | .36<br>.22,<br>.48 | .27<br>.13,<br>.41 | .21<br>.09,<br>.33 | | .59<br>.51,<br>.66 | .43<br>.33,<br>.52 | .53<br>.44,<br>.61 | .56<br>.47,<br>.63 | .38<br>.27,<br>.48 | .42<br>.32,<br>.52 | .12<br>.00,<br>.23 | .14<br>.02,<br>.25 |
| 14 | .43<br>.34,<br>.52 | .50<br>.40,<br>.59 | .51<br>.41,<br>.60 | .71<br>.64,<br>.77 | .52<br>.42,<br>.60 | .51<br>.41,<br>.59 | .48<br>.37,<br>.57 | .34<br>.23,<br>.45 | .35<br>.23,<br>.46 | .46<br>.35,<br>.57 | .32<br>.19,<br>.44 | .46<br>.36,<br>.55 | .61<br>.52,<br>.69 | | .90<br>.88,<br>.92 | .89<br>.87,<br>.91 | .77<br>.72,<br>.81 | .87<br>.84,<br>.90 | .86<br>.83,<br>.89 | .43<br>.33,<br>.51 | .45<br>.35,<br>.53 |
| 15 | .05<br>-.06<br>.16 | .19<br>.07,<br>.31 | .18<br>.06,<br>.30 | .45<br>.34,<br>.54 | .24<br>.13,<br>.35 | .23<br>.11,<br>.34 | .38<br>.27,<br>.48 | .34<br>.23,<br>.44 | .27<br>.14,<br>.39 | .35<br>.22,<br>.46 | .17<br>.03,<br>.30 | .40<br>.29,<br>.49 | .49<br>.38,<br>.58 | .92<br>.90,<br>.94 | | .93<br>.91,<br>.94 | .70<br>.64,<br>.75 | .88<br>.85,<br>.90 | .85<br>.81,<br>.88 | .50<br>.41,<br>.58 | .50<br>.41,<br>.58 |
| 16 | .18<br>.05,<br>.29 | .26<br>.13,<br>.38 | .28<br>.14,<br>.40 | .55<br>.45,<br>.64 | .29<br>.17,<br>.41 | .28<br>.15,<br>.39 | .40<br>.28,<br>.51 | .33<br>.20,<br>.44 | .30<br>.15,<br>.43 | .34<br>.20,<br>.46 | .23<br>.08,<br>.36 | .30<br>.18,<br>.41 | .48<br>.36,<br>.58 | .90<br>.87,<br>.93 | .93<br>.91,<br>.95 | | .67<br>.61,<br>.73 | .82<br>.78,<br>.86 | .81<br>.76,<br>.85 | .48<br>.39,<br>.56 | .49<br>.40,<br>.57 |
| 17 | .30<br>.19,<br>.40 | .40<br>.28,<br>.50 | .39<br>.28,<br>.50 | .60<br>.50,<br>.67 | .42<br>.31,<br>.51 | .40<br>.29,<br>.50 | .36<br>.24,<br>.46 | .34<br>.23,<br>.45 | .28<br>.14,<br>.40 | .34<br>.21,<br>.46 | .20<br>.07,<br>.33 | .38<br>.27,<br>.48 | .44<br>.33,<br>.54 | .87<br>.83,<br>.90 | .83<br>.79,<br>.87 | .81<br>.75,<br>.86 | | .71<br>.65,<br>.77 | .69<br>.62,<br>.75 | .48<br>.39,<br>.56 | .46<br>.37,<br>.55 |
| 18 | .21<br>.09,<br>.32 | .24<br>.11,<br>.36 | .31<br>.19,<br>.43 | .54<br>.44,<br>.63 | .36<br>.25,<br>.47 | .34<br>.22,<br>.45 | .40<br>.29,<br>.51 | .28<br>.16,<br>.40 | .29<br>.15,<br>.42 | .33<br>.19,<br>.46 | .27<br>.13,<br>.40 | .34<br>.22,<br>.44 | .48<br>.37,<br>.58 | .88<br>.85,<br>.91 | .87<br>.84,<br>.90 | .86<br>.81,<br>.90 | .74<br>.68,<br>.80 | | .97<br>.96,<br>.98 | .52<br>.43,<br>.60 | .49<br>.40,<br>.57 |
| 19 | .18<br>.06,<br>.30 | .22<br>.10,<br>.34 | .28<br>.15,<br>.40 | .55<br>.44,<br>.64 | .33<br>.22,<br>.44 | .31<br>.19,<br>.43 | .41<br>.29,<br>.51 | .31<br>.19,<br>.42 | .23<br>.09,<br>.36 | .32<br>.18,<br>.45 | .26<br>.13,<br>.39 | .35<br>.23,<br>.46 | .48<br>.36,<br>.58 | .89<br>.85,<br>.91 | .89<br>.86,<br>.92 | .90<br>.86,<br>.94 | .78<br>.71,<br>.83 | .96<br>.94,<br>.97 | | .51<br>.42,<br>.59 | .49<br>.39,<br>.57 |
| 20 | -.01<br>-.13<br>.12 | .10<br>-.03<br>.23 | .03<br>-.10<br>.17 | .13<br>-.01<br>.26 | .07<br>-.07<br>.19 | .08<br>-.05<br>.21 | .12<br>-.01<br>.26 | .12<br>-.02,<br>.25 | .05<br>-.10,<br>.20 | .14<br>-.01,<br>.29 | .09<br>-.05,<br>.24 | .09<br>-.04,<br>.22 | .06<br>-.08,<br>.20 | .35<br>.22,<br>.46 | .40<br>.28,<br>.50 | .36<br>.23,<br>.47 | .20<br>.06,<br>.33 | .38<br>.26,<br>.49 | .31<br>.18,<br>.43 | | .85<br>.80,<br>.88 |
| 21 | .03<br>-.10<br>.16 | .13<br>-.00<br>.27 | .10<br>-.04<br>.24 | .14<br>.00,<br>.28 | .12<br>-.01<br>.26 | .13<br>-.01<br>.26 | .08<br>-.07<br>.21 | .14<br>.00,<br>.28 | .02<br>-.13,<br>.18 | .04<br>-.12,<br>.20 | .09<br>-.07,<br>.24 | .06<br>-.07,<br>.20 | .07<br>-.07,<br>.21 | .35<br>.23,<br>.47 | .39<br>.27,<br>.50 | .40<br>.27,<br>.51 | .31<br>.18,<br>.43 | .36<br>.23,<br>.47 | .34<br>.21,<br>.46 | .75<br>.67,<br>.82 | |

1=total height, 2=sitting height, 3=knee height, 4=buttock-knee length, 5=foot length left, 6=foot length right, 7=head circumference, 8=head breadth, 9=head length 1, 10=head length 2, 11=face height, 12=shoulder breadth, 13=wrist breadth, 14=weight, 15=BMI, 16=arm circumference, 17=hip breadth, 18=waist circumference relaxed, 19=waist circumference sucking, 20=biceps skinfold, 21=triceps skinfold

Supplemental Table S14. Additive genetic correlations of anthropometric traits in males (upper diagonal matrix) and females (lower diagonal matrix) with 95% confidence intervals in the follow-up assessment under additive genetic/ shared environment/ unique environment model.

| | 1 | 2 | 3 | 4 | 5 | 6 | 7 | 8 | 9 | 10 | 11 | 12 | 13 | 14 | 15 | 16 | 17 | 18 | 19 | 20 | 21 |
|---|---|---|---|---|---|---|---|---|---|---|---|---|---|---|---|---|---|---|---|---|---|
| 1 | | .97<br>.89,<br>1.0 | .98<br>.89,<br>1.00 | .97<br>.83,<br>1.00 | .95<br>.82,<br>NA | .92<br>.79,<br>1.0 | .37<br>.37,<br>.59 | .11<br>-.11<br>.32 | .31<br>-.01<br>.58 | .25<br>-.08<br>.51 | .64<br>.64<br>1.0 | .80<br>.52<br>1.0 | .58<br>.34,<br>.77 | .58<br>.40,<br>.72 | .18<br>-.05<br>.41 | .22<br>.01,<br>.44 | .45<br>.22,<br>.64 | .26<br>-.00,<br>.50 | .28<br>.24,<br>.54 | -.00<br>-.28,<br>.27 | .05<br>-.25,<br>.35 |
| 2 | .83<br>.76,<br>.89 | | .69<br>.49,<br>.84 | .74<br>.50,<br>.94 | .73<br>.53,<br>.89 | .74<br>.54,<br>.91 | .51<br>.51,<br>.73 | .22<br>.00,<br>.41 | .43<br>.11,<br>.70 | .41<br>.10,<br>.66 | .82<br>.81,<br>1.00 | .98<br>.71,<br>1.00 | .68<br>.44,<br>.86 | .73<br>.57,<br>.84 | .36<br>.13,<br>.57 | .42<br>.21,<br>.61 | .61<br>.41,<br>.77 | .37<br>.37,<br>.60 | .47<br>.42,<br>.71 | .04<br>-.25,<br>.32 | .15<br>-.16,<br>.46 |
| 3 | .92<br>.82,<br>NA | .70<br>.57,<br>.84 | | 1.00<br>.87,<br>1.00 | .90<br>.76,<br>1.0 | .90<br>.76,<br>1.0 | .33<br>.33,<br>.57 | .04<br>-.21,<br>.30 | .28<br>-.05,<br>.56 | .32<br>-.01,<br>.60 | .47<br>NA,<br>1.00 | .53<br>.19,<br>.88 | .36<br>.12,<br>.59 | .55<br>.34,<br>.71 | .15<br>-.10<br>.39 | .22<br>-.02<br>.45 | .37<br>.11,<br>.58 | .35<br>.35,<br>.59 | .35<br>.35,<br>.61 | .12<br>-.17,<br>.39 | .09<br>-.23,<br>.38 |
| 4 | .75<br>.61,<br>.88 | .56<br>.38,<br>.71 | .95<br>.74,<br>NA | | .71<br>.50,<br>.90 | .72<br>.49,<br>.90 | .52<br>.52,<br>.76 | .11<br>-.20,<br>.40 | .37<br>.00,<br>.68 | .23<br>-.17,<br>.55 | .73<br>.73,<br>1.00 | .64<br>.28,<br>.98 | .33<br>.05,<br>.58 | .61<br>.57,<br>.77 | .24<br>NA<br>.48 | .30<br>.06,<br>.51 | .57<br>.31,<br>.77 | .46<br>.23,<br>.68 | .39<br>.06,<br>.64 | .21<br>-.12,<br>.49 | .17<br>-.19,<br>.50 |
| 5 | .85<br>.76,<br>.92 | .70<br>.61,<br>.81 | .86<br>.73,<br>.99 | .72<br>.57,<br>.86 | | .99<br>.98,<br>1.0 | .31<br>.07,<br>.55 | .13<br>-.10,<br>.35 | .32<br>-.00,<br>.59 | .33<br>.02,<br>.61 | .38<br>NA,<br>1.00 | .67<br>.32,<br>1.00 | .70<br>.47,<br>.88 | .54<br>.48,<br>.69 | .19<br>.02,<br>.38 | .28<br>.08,<br>.47 | .37<br>.16,<br>.59 | .30<br>.07,<br>.53 | .27<br>.27,<br>.53 | -.04<br>-.33,<br>.24 | -.02<br>-.34,<br>.29 |
| 6 | .85<br>.74,<br>.92 | .71<br>.62,<br>.83 | .83<br>.69,<br>.96 | .76<br>.57,<br>.91 | 1.0<br>.99,<br>NA | | .29<br>.04,<br>.54 | .10<br>-.13,<br>.30 | .41<br>.09,<br>.68 | .41<br>.08,<br>.67 | .58<br>.58,<br>1.00 | .70<br>.36,<br>1.00 | .80<br>.59,<br>.97 | .52<br>.48,<br>.67 | .18<br>.11,<br>.37 | .27<br>.06,<br>.47 | .43<br>.20,<br>.62 | .28<br>.05,<br>.52 | .37<br>.37,<br>.64 | -.03<br>-.32,<br>.25 | .01<br>-.31,<br>.33 |
| 7 | .29<br>.09,<br>.48 | .43<br>.24,<br>.55 | .28<br>.05,<br>.51 | .28<br>.02,<br>.50 | .39<br>.28,<br>.49 | .38<br>.19,<br>.53 | | .69<br>.47,<br>.85 | .90<br>.71,<br>1.00 | .92<br>.70,<br>1.00 | .86<br>.86,<br>1.00 | .56<br>.19,<br>.94 | .26<br>-.01<br>.54 | .59<br>.37,<br>.75 | .50<br>.50,<br>.69 | .40<br>.19,<br>.61 | .55<br>.30,<br>.74 | .39<br>.39,<br>.63 | .35<br>.04,<br>.62 | .09<br>-.22,<br>.38 | .23<br>-.11,<br>.53 |
| 8 | .07<br>-14<br>.28 | .19<br>.06,<br>.31 | .16<br>-.08,<br>.41 | .13<br>-.13,<br>.38 | .16<br>.04,<br>.28 | .17<br>.05,<br>.29 | .60<br>.50,<br>.68 | | .23<br>-.11,<br>.49 | .37<br>.03,<br>.68 | .50<br>.50,<br>1.00 | .23<br>-.15,<br>.56 | .20<br>-.04<br>.48 | .37<br>.15,<br>.57 | .41<br>.17,<br>.60 | .35<br>.13,<br>.56 | .20<br>-.06,<br>.44 | .30<br>.01,<br>.54 | .25<br>.18,<br>.51 | -.04<br>-.34,<br>.24 | .13<br>-.20,<br>.42 |
| 9 | .18<br>-16<br>.50 | .22<br>-.13<br>.49 | .33<br>-.12,<br>.79 | .12<br>-.42,<br>.54 | .25<br>-.09<br>.55 | .13<br>-.28<br>.48 | .79<br>.53,<br>1.00 | .47<br>.15,<br>.87 | | .93<br>.70,<br>1.0 | 1.0<br>1.0<br>1.0 | .39<br>-.05,<br>.82 | .44<br>.09,<br>.77 | .44<br>.16,<br>.67 | .37<br>.08,<br>.62 | .28<br>-.02<br>.54 | .48<br>.19,<br>.72 | .27<br>-.08,<br>.56 | .26<br>-.13,<br>.60 | .05<br>-.29,<br>.38 | .23<br>-.16,<br>.58 |
| 10 | .40<br>.10,<br>.70 | .51<br>.29,<br>.77 | .30<br>-.06,<br>.69 | .21<br>-.22,<br>.61 | .43<br>.16,<br>.74 | .35<br>.03,<br>.69 | .78<br>.53,<br>1.00 | .43<br>.15,<br>.77 | .80<br>.42,<br>1.00 | | .71<br>.71,<br>1.00 | .56<br>.12,<br>.98 | .54<br>.21,<br>.84 | .57<br>.53,<br>.77 | .51<br>.24,<br>.73 | .34<br>.06,<br>.57 | .52<br>.23,<br>.76 | .35<br>.00,<br>.63 | .33<br>.30,<br>.64 | -.02<br>-.38,<br>.33 | .12<br>-.29,<br>.48 |

| | | | | | | | | | | | | | | | | | | | | | |
|---|---|---|---|---|---|---|---|---|---|---|---|---|---|---|---|---|---|---|---|---|---|
| 11 | .22<br>.11,<br>.45 | .39<br>.29,<br>.59 | .27<br>.17,<br>.53 | .43<br>.43,<br>.78 | .36<br>.28,<br>.53 | .34<br>.21,<br>.54 | .41<br>.36,<br>.61 | .29<br>.05,<br>.60 | .63<br>.63,<br>1.00 | .27<br>.27,<br>.61 | | .60<br>NA,<br>1.00 | .34<br>-1.0<br>1.0 | .93<br>.81,<br>NA | .77<br>.77<br>1.0 | .70<br>.70,<br>1.0 | .88<br>.68,<br>1.0 | .63<br>.63,<br>1.00 | .83<br>NA,<br>NA | .45<br>NA,<br>1.00 | .40<br>-1.0<br>1.00 |
| 12 | .32<br>-01<br>.59 | .28<br>-.07<br>.58 | .36<br>-.04,<br>.74 | .30<br>.30,<br>.63 | .43<br>.43,<br>.71 | .49<br>.17,<br>.79 | .29<br>.27,<br>.61 | .04<br>-.33,<br>.36 | .20<br>-.36,<br>.67 | .53<br>.06,<br>.98 | .20<br>-.26,<br>.67 | | .82<br>.48<br>1.0 | .76<br>.49,<br>1.0 | .49<br>NA<br>.79 | .57<br>.26,<br>.89 | .68<br>.68,<br>.99 | .39<br>.39,<br>.69 | .41<br>.41,<br>.79 | .38<br>.00,<br>.76 | .44<br>.02,<br>.89 |
| 13 | .52<br>.25,<br>.72 | .45<br>.23,<br>.66 | .55<br>.21,<br>.82 | .62<br>.29,<br>.88 | .60<br>.38,<br>.80 | .58<br>.32,<br>.78 | .50<br>.21,<br>.77 | .38<br>.09,<br>.64 | .31<br>-.18,<br>.70 | .57<br>.14,<br>1.00 | .26<br>.26,<br>.68 | .34<br>.32,<br>.69 | | .55<br>.35,<br>.74 | .37<br>.37,<br>.60 | .50<br>.30,<br>.71 | .59<br>.34,<br>.80 | .27<br>.01,<br>.54 | .34<br>.32,<br>.63 | .07<br>-.24,<br>.37 | .17<br>-.18,<br>.49 |
| 14 | .55<br>.34,<br>.70 | .54<br>.36,<br>.70 | .58<br>.32,<br>.79 | .71<br>.51,<br>.85 | .53<br>.36,<br>.67 | .47<br>.28,<br>.63 | .54<br>.32,<br>.70 | .38<br>.16,<br>.56 | .46<br>.10,<br>.78 | .67<br>.36,<br>.95 | .26<br>.26,<br>.57 | .43<br>NA,<br>.69 | .67<br>.42,<br>.87 | | .91<br>.85,<br>.94 | .90<br>.83,<br>.94 | .88<br>.78,<br>.94 | .87<br>.87,<br>.94 | .91<br>.84,<br>1.00 | .47<br>.23,<br>.66 | .64<br>.40,<br>.83 |
| 15 | .18<br>-08<br>.41 | .27<br>.03,<br>.48 | .26<br>-.05,<br>.54 | .47<br>.40,<br>.68 | .22<br>.01,<br>.42 | .14<br>-.09<br>.37 | .51<br>.28,<br>.69 | .42<br>.18,<br>.60 | .46<br>.08,<br>.81 | .60<br>.27,<br>.90 | .28<br>.28,<br>.57 | .35<br>NA,<br>.56 | .56<br>.26,<br>.80 | .92<br>.92,<br>.95 | | .97<br>.93,<br>1.0 | .81<br>.69,<br>.89 | .91<br>.91,<br>.97 | .97<br>.87,<br>1.00 | .56<br>.34,<br>.72 | .72<br>.51,<br>.89 |
| 16 | .27<br>-01<br>.52 | .29<br>.06,<br>.52 | .36<br>-.01,<br>.69 | .66<br>.38,<br>.88 | .33<br>.11,<br>.54 | .27<br>.03,<br>.50 | .49<br>.26,<br>.71 | .43<br>.19,<br>.64 | .45<br>.00,<br>.94 | .62<br>.30,<br>1.00 | .19<br>.19,<br>.54 | .26<br>-.09,<br>.60 | .54<br>.21,<br>.84 | .96<br>.88,<br>1.0 | .97<br>.90,<br>1.0 | | .73<br>.57,<br>.84 | .86<br>.74,<br>.95 | .88<br>.74,<br>.99 | .48<br>.25,<br>.67 | .70<br>.47,<br>.89 |
| 17 | .43<br>.16,<br>.66 | .56<br>.31,<br>.79 | .48<br>.14,<br>.77 | .45<br>.18,<br>.69 | .41<br>.18,<br>.62 | .29<br>.05,<br>.53 | .53<br>.26,<br>.75 | .40<br>.14,<br>.62 | .51<br>.09,<br>.95 | .79<br>.44,<br>1.00 | .37<br>-.02,<br>.76 | .47<br>.47,<br>.81 | .60<br>.27,<br>.86 | .97<br>.87,<br>1.0 | .94<br>.83,<br>1.0 | .91<br>.72,<br>1.0 | | .72<br>.72,<br>.86 | .79<br>.63,<br>.96 | .45<br>.20,<br>.66 | .64<br>.38,<br>.86 |
| 18 | .29<br>.03,<br>.49 | .24<br>.11,<br>.36 | .34<br>.06,<br>.63 | .51<br>.51,<br>.70 | .36<br>.25,<br>.51 | .33<br>.12,<br>.48 | .41<br>.25,<br>.58 | .30<br>.10,<br>.50 | .43<br>.43,<br>.88 | .53<br>.22,<br>.88 | .27<br>.27,<br>.31 | .36<br>NA,<br>.67 | .60<br>.33,<br>.86 | .86<br>.82,<br>.91 | .85<br>.79,<br>.91 | .85<br>.78,<br>.94 | .78<br>.60,<br>.92 | | .97<br>.97,<br>1.00 | .60<br>.35,<br>.78 | .61<br>.33,<br>.83 |
| 19 | .38<br>.28,<br>.64 | .39<br>.08,<br>.65 | .42<br>.42,<br>.84 | .54<br>.54,<br>.79 | .39<br>.13,<br>.63 | .28<br>.28,<br>.45 | .51<br>NA,<br>.76 | .26<br>-.06,<br>.53 | .26<br>-.32,<br>.67 | .70<br>.32,<br>1.00 | .38<br>NA,<br>.76 | .46<br>.46,<br>.91 | .63<br>.25,<br>.96 | .96<br>.86,<br>1.0 | .95<br>.95,<br>1.0 | .97<br>.81,<br>NA | .94<br>.94,<br>1.0 | 1.00<br>.94,<br>NA | | .63<br>.36,<br>.84 | .61<br>.28,<br>.88 |
| 20 | .25<br>-1<br>1.0 | .56<br>.11,<br>1.0 | .29<br>-1.0<br>1.00 | .60<br>.04,<br>1.00 | .12<br>-1.0<br>1.0 | .25<br>-1.0<br>1.0 | .32<br>-1.0<br>1.00 | .65<br>.13,<br>1.00 | .17<br>-1.0<br>1.0 | .63<br>-.03,<br>1.00 | .48<br>.48,<br>1.00 | .02<br>-1.0,<br>1.0 | .54<br>-.14,<br>1.0 | .93<br>.50,<br>NA | .96<br>.53,<br>NA | 1.0<br>.66,<br>NA | 1.00<br>NA,<br>NA | .67<br>.20,<br>1.0 | .32<br>-1.0<br>1.0 | | .77<br>.63,<br>.93 |
| 21 | .44<br>.01,<br>1.0 | .54<br>.11,<br>1.00 | .17<br>.17,<br>1.00 | .60<br>.18,<br>1.00 | .29<br>.29,<br>1.0 | .29<br>.29,<br>1.0 | .15<br>-1.0,<br>1.0 | .08<br>-1.0,<br>1.0 | .02<br>-1.0,<br>1.00 | -.08<br>-1.0,<br>1.00 | -.02<br>-1.0,<br>1.00 | .25<br>.25,<br>1.00 | .28<br>.28,<br>1.0 | .81<br>.43,<br>1.0 | .72<br>.72,<br>1.0 | .85<br>.41,<br>NA | .89<br>.42,<br>NA | .65<br>.65,<br>1.00 | .81<br>.81,<br>1.00 | 1.00<br>NA,<br>NA | |

1=total height, 2=sitting height, 3=knee height, 4=buttock-knee length, 5=foot length left, 6=foot length right, 7=head circumference, 8=head breadth, 9=head length 1, 10=head length 2, 11=face height, 12=shoulder breadth, 13=wrist breadth, 14=weight, 15=BMI, 16=arm circumference, 17=hip breadth, 18=waist circumference relaxed, 19=waist circumference sucking, 20=biceps skinfold, 21=triceps skinfold

Supplemental Table S15. Shared environmental correlations of anthropometric traits in males (upper diagonal matrix) and females (lower diagonal matrix) with 95% confidence intervals in the follow-up assessment under the additive genetic/ shared environment/ unique environment model.

| | 1 | 2 | 3 | 4 | 5 | 6 | 7 | 8 | 9 | 10 | 11 | 12 | 13 | 14 | 15 | 16 | 17 | 18 | 19 | 20 | 21 |
|---|---|---|---|---|---|---|---|---|---|---|---|---|---|---|---|---|---|---|---|---|---|
| 1 | | .51<br>-1.0<br>1.0 | .68<br>-1.0<br>.90 | .73<br>-1.0<br>1.00 | .19<br>-1.0<br>1.00 | .38<br>-1.0<br>1.00 | .74<br>-1.0<br>1.00 | 1.00<br>.39,<br>NA | .29<br>-1.0<br>1.00 | .75<br>.22,<br>1.00 | .59<br>.59,<br>1.00 | .26<br>-1.0<br>1.00 | .53<br>-1.0,<br>1.00 | .84<br>-1.0<br>1.00 | .52<br>-1.0,<br>1.00 | .97<br>-1.0<br>NA | .57<br>-1.0<br>1.00 | .78<br>-1.0<br>1.00 | .75<br>.75,<br>1.00 | .28<br>NA,<br>1.00 | .25<br>-1.0<br>1.00 |
| 2 | 1.00<br>-1.0<br>NA | | .39<br>-1.0<br>1.00 | .49<br>NA,<br>1.00 | .24<br>-1.0<br>1.00 | .33<br>-1.0<br>1.00 | .52<br>-1.0<br>1.00 | 1.00<br>1.00,<br>NA | .23<br>-1.0,<br>1.00 | .87<br>.27,<br>NA | .56<br>-1.0<br>1.00 | .20<br>-1.0<br>1.00 | .09<br>-1.0<br>1.00 | .61<br>-1.0<br>1.00 | .54<br>-1.0<br>1.00 | .84<br>-1.0<br>NA | .32<br>-1.0<br>1.00 | .52<br>-1.0<br>1.00 | .40<br>-1.0,<br>1.00 | .21<br>-1.0<br>1.00 | .10<br>-1.0<br>1.00 |
| 3 | .83<br>-1.0<br>1.00 | 1.00<br>-1.0<br>NA | | .65<br>-1.0<br>1.00 | .28<br>-1.0<br>1.00 | .37<br>-1.0<br>1.00 | .51<br>-1.0<br>1.00 | .92<br>.77,<br>NA | .24<br>-1.0<br>1.00 | .51<br>-1.0<br>1.00 | .53<br>-1.0<br>1.00 | .42<br>-1.0<br>1.00 | .92<br>-1.0<br>NA | .61<br>-1.0<br>1.00 | .41<br>-1.0<br>1.00 | .58<br>-1.0<br>1.00 | .48<br>-1.0<br>1.00 | .45<br>-1.0<br>1.00 | .52<br>-1.0<br>1.00 | -.17<br>-1.0<br>1.00 | .08<br>-1.0<br>1.00 |
| 4 | .93<br>-1.0<br>NA | 1.00<br>-1.0<br>NA | .32<br>-1.0<br>1.00 | | .94<br>-1.0<br>1.00 | .79<br>-1.0<br>1.00 | .50<br>-1.0<br>1.00 | .77<br>-1.0<br>NA | .34<br>-.33,<br>.86 | .76<br>.37,<br>1.00 | .51<br>.51,<br>.99 | .32<br>NA,<br>.62 | 1.00<br>1.00,<br>NA | .99<br>.98,<br>NA | .99<br>.99,<br>NA | 1.00<br>.79,<br>NA | .49<br>.49,<br>.90 | .84<br>.84,<br>1.00 | .90<br>.90,<br>1.00 | .19<br>-1.0<br>1.00 | .33<br>-.16,<br>.91 |
| 5 | 1.00<br>-1.0<br>NA | -1.0<br>NA,<br>1.00 | 1.00<br>-1.0<br>NA | 1.00<br>-1.0<br>NA | | 1.00<br>-1.0<br>NA | .95<br>-1.0<br>NA | 1.00<br>NA,<br>NA | .46<br>NA,<br>1.00 | .83<br>-1.0<br>1.00 | .96<br>NA,<br>NA | .30<br>-1.0<br>1.00 | .31<br>-1.0<br>1.00 | 1.00<br>-1.0,<br>NA | 1.00<br>-1.0,<br>NA | 1.00<br>-1.0,<br>NA | .97<br>-1.0,<br>NA | 1.00<br>-1.0,<br>NA | 1.00<br>NA,<br>NA | .45<br>-1.0,<br>1.00 | .37<br>-1.0,<br>1.00 |
| 6 | -.20<br>-1.0<br>1.00 | -1.0<br>NA,<br>1.00 | 1.00<br>-1.0<br>NA | 1.00<br>-1.0<br>NA | 1.00<br>NA,<br>NA | | .90<br>-1.0<br>NA | 1.00<br>.49,<br>NA | .18<br>-1.0<br>1.00 | .60<br>-1.0<br>1.00 | .66<br>-1.0<br>1.00 | .33<br>-1.0<br>1.00 | -.02<br>-1.0<br>1.00 | 1.00<br>-1.0,<br>NA | 1.00<br>NA,<br>NA | 1.00<br>-1.0,<br>NA | .78<br>-1.0,<br>NA | 1.00<br>-1.0,<br>NA | .69<br>NA,<br>1.00 | .46<br>-1.0,<br>1.00 | .32<br>-1.0,<br>1.00 |
| 7 | 1.00<br>-1.0<br>NA | 1.00<br>-1.0<br>NA | 1.00<br>-1.0<br>NA | 1.00<br>NA,<br>NA | .05<br>-1.0<br>NA | 1.00<br>-1.0<br>NA | | .56<br>-1.0<br>1.00 | .55<br>-1.0,<br>1.00 | .32<br>-1.0<br>1.00 | .46<br>-1.0<br>1.00 | .28<br>-1.0<br>1.00 | .96<br>NA,<br>NA | .60<br>-1.0,<br>1.00 | .40<br>-1.0,<br>1.00 | .92<br>-1.0,<br>NA | .19<br>-1.0,<br>1.00 | .71<br>-1.0,<br>1.00 | .81<br>NA,<br>1.00 | .27<br>-1.0,<br>1.00 | .05<br>-1.0,<br>1.00 |
| 8 | 1.00<br>-1.0<br>NA | .90<br>-1.0<br>NA | -1.0<br>NA,<br>1.00 | 1.00<br>-1.0<br>NA | .83<br>-1.0<br>NA | -.89<br>NA,<br>1.00 | 1.00<br>-1.0<br>NA | | .60<br>-1.0<br>1.00 | .36<br>-1.0<br>1.00 | .56<br>-1.0<br>NA | .33<br>-1.0<br>1.00 | .93<br>-1.0<br>NA | .75<br>-1.0,<br>1.00 | .17<br>-1.0,<br>1.00 | .67<br>-1.0,<br>NA | .60<br>-1.0,<br>1.00 | .35<br>-1.0,<br>1.00 | .73<br>-1.0,<br>1.00 | .50<br>-1.0,<br>1.00 | .14<br>-1.0,<br>1.00 |
| 9 | .62<br>NA,<br>1.00 | 1.00<br>-1.0<br>NA | .09-<br>1.00,<br>NA | .70<br>.70,<br>1.00 | 1.00<br>-1.0,<br>NA | .70<br>-1.0<br>1.00 | 1.00<br>-1.0<br>NA | -1.0<br>NA,<br>1.00 | | .70<br>.24,<br>.92 | .12<br>-.92,<br>.12 | .20<br>-.30,<br>.63 | -.02<br>-1.0<br>1.00 | .28<br>-1.0,<br>1.00 | .22<br>-1.0,<br>1.00 | .57<br>-1.0,<br>1.00 | -.31<br>-1.0,<br>.18 | .23<br>-1.0,<br>1.00 | .30<br>.30,<br>.91 | .01<br>-1.0,<br>1.00 | -.06<br>-.79,<br>.43 |
| 10 | .40<br>-1.0<br>1.00 | 1.00<br>-1.0<br>NA | .70<br>-1.0<br>NA | .74<br>-1.0<br>NA | -1.0<br>NA,<br>1.00 | .43<br>-1.0<br>1.00 | -1.0<br>NA,<br>1.00 | -1.0<br>NA,<br>1.00 | .61<br>-1.0<br>1.00 | | .56<br>.56,<br>1.00 | .27<br>-.14,<br>.57 | .26<br>-1.0<br>1.00 | .69<br>-1.0<br>1.00 | .56<br>-1.0,<br>1.00 | 1.00<br>.84,<br>NA | .08<br>-.82,<br>.47 | .60<br>-1.0,<br>1.00 | .64<br>.64,<br>1.00 | .48<br>-.07,<br>1.00 | .35<br>-.09,<br>.84 |

| | | | | | | | | | | | | | | | | | | | | | |
|---|---|---|---|---|---|---|---|---|---|---|---|---|---|---|---|---|---|---|---|---|---|
| 11 | 1.00<br>1.00,<br>NA | 1.00<br>-1.0<br>NA | 1.00<br>-1.0<br>NA | .10<br>-1.0<br>1.00 | 1.00<br>-1.0<br>NA | 1.00<br>-1.0<br>NA | 1.00<br>-1.0<br>NA | -1.0<br>NA,<br>1.00 | -.07<br>-1.0<br>1.00 | 1.00<br>-1.0<br>NA | | .34<br>.34,<br>.73 | .82<br>-1.0<br>NA | .44<br>-.54,<br>1.00 | .27<br>-1.0,<br>1.00 | .65<br>.35,<br>1.00 | .14<br>-.82,<br>.14 | .35-<br>.99,<br>1.00 | .30-<br>.60,<br>NA | -.05<br>-1.0,<br>.58 | .08<br>-.32,<br>.58 |
| 12 | .32<br>-1.0<br>1.00 | 1.00<br>-1.0<br>NA | .80<br>NA,<br>1.00 | .25<br>-1.0<br>1.00 | 1.00<br>NA,<br>NA | 1.00<br>-1.0<br>NA | 1.00<br>-1.0<br>NA | 1.00<br>-1.0<br>NA | -.03<br>-1.0,<br>1.00 | .10<br>-1.0<br>1.00 | 1.00<br>-1.0<br>NA | | .19<br>-1.0<br>1.00 | .33<br>-1.0,<br>1.00 | .33<br>-1.0,<br>1.00 | .38<br>-1.0,<br>1.00 | .33<br>.33,<br>.33 | .28<br>-1.0,<br>1.00 | .32<br>NA,<br>.84 | -.24<br>-1.0,<br>.18 | -.05<br>-.50,<br>.29 |
| 13 | .03<br>-1.0<br>1.00 | 1.00<br>NA,<br>NA | -.22<br>NA,<br>1.00 | .13<br>NA,<br>1.00 | -1.0<br>NA,<br>1.00 | -1.0<br>NA,<br>1.00 | -1.0<br>NA,<br>1.00 | -1.0<br>NA,<br>1.00 | -.07<br>-1.0<br>1.00 | -.39<br>-1.0<br>1.00 | .42<br>-1.0,<br>1.00 | .13<br>-1.0,<br>1.00 | | .79<br>-1.0,<br>1.00 | .81<br>-1.0,<br>1.00 | .76<br>-1.0,<br>NA | .53<br>-1.0,<br>1.00 | .95<br>-1.0,<br>NA | .77<br>-1.0,<br>1.00 | .30<br>-1.0,<br>1.00 | .10<br>-1.0,<br>1.00 |
| 14 | -.16<br>-1.0<br>1.00 | 1.00<br>-1.0<br>NA | .20<br>-1.0<br>1.00 | .72<br>-1.0<br>1.00 | 1.00<br>-1.0<br>NA | 1.00<br>NA,<br>NA | -1.0<br>NA,<br>NA | -1.0<br>NA,<br>1.00 | .06<br>-1.0<br>1.00 | -.39<br>-1.0<br>1.00 | 1.00<br>-1.0,<br>NA | .73<br>.73,<br>1.00 | .35<br>-1.0<br>1.00 | | .91<br>-1.0,<br>1.00 | .93<br>-1.0,<br>NA | .50<br>-1.0,<br>NA | .90<br>-1.0,<br>1.00 | .81<br>-1.0,<br>1.00 | .32<br>-1.0,<br>1.00 | .11<br>-1.0,<br>1.00 |
| 15 | -.48<br>-1.0<br>.48 | -1.0<br>NA,<br>1.00 | -.12<br>-1.0<br>1.00 | .39<br>-1.0,<br>1.00 | 1.00<br>NA,<br>NA | 1.00<br>-1.0<br>NA | -1.0<br>NA,<br>.93 | -1.0<br>NA,<br>1.00 | -.15<br>-1.0<br>NA | -.50<br>-1.0<br>1.00 | -.92<br>NA,<br>1.00 | .54<br>.54,<br>1.00 | .29<br>NA,<br>1.00 | .94<br>-1.0,<br>NA | | .65<br>.65,<br>1.00 | .40<br>-1.0,<br>1.00 | .81<br>-1.0,<br>1.00 | .64<br>-1.0,<br>1.00 | .33<br>-1.0,<br>1.00 | .06<br>-1.0,<br>1.00 |
| 16 | -.26<br>-1.0<br>1.00 | -1.0<br>NA,<br>1.00 | .04<br>-1.0<br>1.00 | .11<br>-1.0<br>1.00 | -1.0<br>NA,<br>1.00 | 1.00<br>-1.0<br>NA | -1.0<br>NA,<br>1.00 | -1.0<br>NA,<br>1.00 | -.15<br>-1.0<br>1.00 | -1.0<br>NA,<br>1.00 | .99<br>-1.0,<br>NA | .53<br>-1.0,<br>1.00 | .23<br>-1.0,<br>1.00 | .63<br>-1.0,<br>1.00 | .81<br>-1.0,<br>1.00 | | .60<br>-1.0,<br>1.00 | .84<br>-1.0,<br>1.00 | .80<br>-1.0,<br>1.00 | .61<br>-1.0,<br>1.00 | .15<br>-1.0,<br>1.00 |
| 17 | -.02<br>-1.0<br>1.00 | -1.0<br>NA,<br>1.00 | .24<br>-1.0<br>1.00 | 1.00<br>NA,<br>NA | 1.00<br>-1.0<br>NA | 1.00<br>NA,<br>NA | -1.0<br>NA,<br>1.00 | 1.00<br>NA,<br>NA | -.12<br>-1.0<br>1.00 | -.91<br>NA,<br>-.66 | -1.0<br>NA,<br>1.00 | .36<br>.36,<br>.73 | .11<br>-1.0,<br>1.00 | .73<br>-1.0,<br>1.00 | .67<br>-.97,<br>1.00 | .65<br>-1.0,<br>1.00 | | .71<br>-1.0,<br>1.00 | .52-<br>.61,<br>.86 | .54<br>.54,<br>1.00 | .22<br>-.50,<br>.61 |
| 18 | -1.0<br>NA,<br>1.00 | .86<br>NA,<br>NA | 1.00<br>-1.0<br>NA | 1.00<br>-1.0<br>NA | .99<br>-1.0<br>NA | 1.00<br>-1.0<br>NA | -1.0<br>NA,<br>1.00 | -1.0<br>NA,<br>1.00 | -1.0<br>NA,<br>1.00 | -1.0<br>NA,<br>1.00 | 1.00<br>-1.0<br>NA | 1.00<br>-1.0,<br>NA | -1.0<br>NA,<br>1.00 | 1.00<br>-1.0,<br>NA | 1.00<br>-1.0,<br>NA | 1.00<br>-1.0,<br>NA | 1.00<br>-1.0,<br>NA | | .99<br>.94,<br>NA | .34<br>-1.0,<br>1.00 | .31<br>-1.0,<br>1.00 |
| 19 | -.22<br>-1.0-<br>.22 | -1.0<br>NA,<br>1.00 | .07<br>-1.0<br>1.00 | .63<br>-1.0,<br>1.00 | 1.00<br>-1.0<br>NA | 1.00<br>NA,<br>NA | 1.00<br>-1.0<br>NA | 1.00<br>-1.0<br>NA | .18<br>-1.0<br>1.00 | -1.0<br>NA,<br>1.00 | -1.0<br>NA,<br>1.00 | .34<br>.34,<br>.50 | .19<br>-1.0<br>1.00 | .81<br>-1.0,<br>1.00 | .84<br>-1.0,<br>1.00 | .86<br>-1.0,<br>NA | .57<br>-.98,<br>.85 | 1.00<br>.95,<br>NA | | .30<br>-1.0,<br>.30 | .36<br>-.33,<br>.62 |
| 20 | -.26<br>-1.0<br>1.00 | -1.0<br>NA,<br>1.00 | -.26<br>-1.0<br>1.00 | -.43<br>-1.0<br>1.00 | 1.00<br>-1.0<br>NA | -1.0<br>NA,<br>1.00 | -1.0<br>NA,<br>NA | -1.0<br>NA,<br>1.00 | -.04<br>-1.0,<br>1.00 | -1.0<br>NA,<br>1.00 | -1.0<br>NA,<br>1.00 | .12<br>-.17,<br>.39 | -.39<br>-1.0,<br>1.00 | -.16<br>-1.0,<br>1.00 | .04<br>-1.0,<br>1.00 | -.22<br>-1.0,<br>.29 | -.42<br>-1.0,<br>.04 | 1.00<br>-1.0,<br>NA | .34<br>NA,<br>.49 | | .99<br>.76,<br>NA |
| 21 | -.52<br>NA,<br>.06 | -1.0<br>NA,<br>1.00 | .04<br>-1.0<br>1.00 | -.63<br>-1.0<br>1.00 | -1.0<br>NA,<br>1.00 | -.98<br>NA,<br>.88 | -1.0<br>NA,<br>1.00 | 1.00<br>-1.0<br>NA | .04<br>-1.0,<br>1.00 | 1.00<br>-1.0,<br>NA | 1.00<br>-1.0,<br>NA | -.02<br>-.40,<br>.30 | -.22<br>-1.0,<br>1.00 | -.25<br>-1.0,<br>1.00 | .07<br>-1.0,<br>1.00 | -.06<br>-1.0,<br>1.00 | -.19<br>-1.0,<br>.27 | -1.0<br>NA,<br>1.00 | .04<br>-.92,<br>NA | .66<br>.38,<br>.83 | |

1=total height, 2=sitting height, 3=knee height, 4=buttock-knee length, 5=foot length left, 6=foot length right, 7=head circumference, 8=head breadth, 9=head length 1, 10=head length 2, 11=face height, 12=shoulder breadth, 13=wrist breadth, 14=weight, 15=BMI, 16=arm circumference, 17=hip breadth, 18=waist circumference relaxed, 19=waist circumference sucking, 20=biceps skinfold, 21=triceps skinfold

Supplemental Table S16. Unique environmental correlations of anthropometric traits in males (upper diagonal matrix) and females (lower diagonal matrix) with 95% confidence intervals in the follow-up assessment under the additive genetic / unique environment model.

| | 1 | 2 | 3 | 4 | 5 | 6 | 7 | 8 | 9 | 10 | 11 | 12 | 13 | 14 | 15 | 16 | 17 | 18 | 19 | 20 | 21 |
|---|---|---|---|---|---|---|---|---|---|---|---|---|---|---|---|---|---|---|---|---|---|
| 1 | | .55 .45, .63 | .43 .31, .53 | .36 .24, .47 | .33 .20, .44 | .34 .22, .45 | .39 .27, .50 | .14 .01, .27 | .28 .16, .40 | .41 .30, .52 | .17 .04, .29 | .23 .11, .35 | .30 .17, .42 | .48 .37, .58 | .00 -.13, .14 | .36 .23, .47 | .27 .15, .39 | .24 .11, .36 | .20 .07, .32 | .01 -.12, .15 | .00 -.13, .14 |
| 2 | .59 .48, .68 | | .32 .20, .44 | .17 .04, .29 | .26 .13, .38 | .18 .05, .31 | .21 .08, .34 | .03 -.11, .16 | .19 .06, .31 | .31 .19, .43 | .15 .02, .27 | .19 .06, .31 | .31 .19, .43 | .44 .32, .54 | .22 .09, .35 | .39 .26, .50 | .36 .24, .47 | .23 .10, .35 | .21 .08, .34 | -.03 -.17, .10 | -.05 -.18, .09 |
| 3 | .32 .17, .45 | .09 .06, .24 | | .21 .09, .33 | .38 .27, .49 | .31 .18, .42 | .29 .16, .41 | .15 .01, .27 | .23 .10, .35 | .23 .10, .35 | .19 .06, .31 | .07 -.07, .20 | .33 .20, .44 | .39 .27, .50 | .22 .09, .35 | .22 .08, .34 | .21 .08, .34 | .21 .08, .34 | .20 .06, .32 | .09 -.04, .22 | .14 .01, .27 |
| 4 | .37 .23, .49 | .12 .04, .27 | .14 .00, .29 | | .25 .12, .37 | .29 .16, .41 | .26 .13, .38 | .20 .07, .32 | .19 .06, .31 | .33 .21, .44 | .05 -.08, .18 | .24 .11, .36 | .21 .08, .33 | .49 .38, .58 | .37 .25, .48 | .38 .26, .49 | .23 .11, .36 | .36 .24, .47 | .35 .23, .46 | .25 .12, .37 | .17 .04, .29 |
| 5 | .42 .29, .54 | .30 .15, .43 | .30 .15, .43 | .33 .19, .46 | | .73 .66, .78 | .17 .04, .30 | -.01 -.15, .12 | .23 .10, .35 | .24 .11, .36 | .11 -.02, .24 | .04 -.09, .17 | .25 .13, .37 | .30 .18, .42 | .18 .05, .31 | .23 .10, .36 | .24 .11, .36 | .13 -.00, .26 | .11 -.02, .24 | .13 -.01, .26 | .07 -.06, .20 |
| 6 | .42 .29, .54 | .26 .11, .40 | .29 .15, .43 | .34 .20, .46 | .60 .52, .68 | | .22 .09, .34 | -.02 -.16, .11 | .23 .10, .35 | .21 .08, .33 | -.02 -.15, .11 | .03 -.10, .16 | .20 .07, .32 | .34 .21, .45 | .20 .07, .33 | .27 .14, .39 | .20 .07, .33 | .12 -.02, .25 | .08 -.05, .21 | .20 .06, .32 | .09 -.04, .22 |
| 7 | .21 .06, .35 | .15 -.01 .30 | .07 -.08 .22 | .27 .12, .41 | .26 .11, .40 | .18 .03, .33 | | .41 .29, .51 | .48 .38, .58 | .36 .24, .47 | .11 -.02, .24 | .11 -.02, .24 | .14 .00, .26 | .48 .37, .58 | .34 .22, .46 | .39 .27, .49 | .31 .19, .43 | .29 .17, .41 | .24 .11, .36 | .14 .01, .27 | .25 .12, .37 |
| 8 | .13 -.03 .28 | .15 -.01 .30 | .04 -.11 .20 | .16 .00, .30 | .14 -.01 .29 | .09 -.07 .24 | .18 .02, .32 | | .14 .01, .26 | .07 -.06, .20 | .09 -.04, .21 | .20 .07, .32 | .09 -.04, .22 | .37 .25, .48 | .33 .21, .45 | .21 .08, .34 | .29 .17, .41 | .28 .16, .40 | .23 .10, .35 | .22 .08, .34 | .23 .10, .35 |
| 9 | .32 .18, .45 | .29 .14, .42 | .04 -.11 .19 | .26 .12, .40 | .31 .17, .44 | .25 .10, .39 | .34 .20, .47 | .14 -.02, .28 | | .44 .33, .54 | -.12 -.24, .01 | .09 -.04, .22 | .05 -.08, .18 | .36 .24, .47 | .26 .13, .38 | .28 .15, .40 | .29 .16, .41 | .21 .08, .34 | .15 .02, .28 | .12 -.01, .25 | .12 -.01, .25 |
| 10 | .08 -.07 , .23 | .14 -.01 , .29 | .08 -.07 .22 | .16 .01, .30 | .03 -.12 .18 | .07 -.09 .21 | .31 .17, .44 | -.03 -.18, .12 | .33 .20, .46 | | .07- .06, .19 | .14 .01, .26 | .17 .04, .30 | .48 .37, .57 | .37 .25, .48 | .49 .38, .59 | .34 .22, .45 | .30 .17, .41 | .29 .17, .41 | .15 .02, .27 | .15 .02, .27 |

| | | | | | | | | | | | | | | | | | | | | | |
|---|---|---|---|---|---|---|---|---|---|---|---|---|---|---|---|---|---|---|---|---|---|
| 11 | .09<br>-.07<br>.24 | .03<br>-.13<br>.18 | .05<br>-.10<br>.20 | .08<br>-.07<br>.23 | .12<br>-.03<br>.27 | .09<br>-.06<br>.24 | .17<br>.01,<br>.31 | -.03<br>-.18,<br>.12 | .00<br>-.14,<br>.15 | -.03<br>-.18,<br>.12 | | .07<br>-.06,<br>.19 | .16<br>.03,<br>.28 | .14<br>.02,<br>.27 | .08<br>-.05,<br>.21 | .04<br>-.09,<br>.17 | .10<br>-.03,<br>.22 | .13<br>-.00,<br>.25 | .11<br>-.02,<br>.23 | .05<br>-.08,<br>.17 | .06<br>-.07,<br>.19 |
| 12 | .27<br>.12,<br>.40 | .21<br>.06,<br>.35 | .05<br>-.10<br>.20 | .32<br>.18,<br>.45 | .20<br>.05,<br>.34 | .14<br>-.01<br>.28 | .20<br>.05,<br>.34 | .21<br>.06,<br>.35 | .20<br>.05,<br>.34 | .12<br>-.03,<br>.26 | -.02<br>-.17,<br>.13 | | .14<br>.01,<br>.26 | .28<br>.15,<br>.40 | .20<br>.07,<br>.33 | .16<br>.03,<br>.29 | .23<br>.10,<br>.35 | .25<br>.12,<br>.37 | .17<br>.05,<br>.30 | .05<br>-.08,<br>.18 | -.06<br>-.19,<br>.07 |
| 13 | .36<br>.22,<br>.48 | .17<br>.02,<br>.31 | .15<br>.01,<br>.30 | .17<br>.03,<br>.31 | .25<br>.10,<br>.38 | .26<br>.11,<br>.39 | .06<br>-.09<br>.21 | .10<br>-.05,<br>.25 | .26<br>.11,<br>.39 | -.04<br>-.19,<br>.11 | -.02<br>-.16,<br>.13 | .23<br>.08,<br>.37 | | .29<br>.17,<br>.41 | .18<br>.05,<br>.31 | .15<br>.02,<br>.28 | .16<br>.03,<br>.29 | .14<br>.01,<br>.27 | .12<br>-.02,<br>.24 | .05<br>-.09,<br>.18 | .09<br>-.04,<br>.22 |
| 14 | .34<br>.19,<br>.47 | .28<br>.13,<br>.42 | .10<br>-.05<br>.25 | .52<br>.40,<br>.62 | .24<br>.09,<br>.38 | .27<br>.12,<br>.41 | .34<br>.20,<br>.47 | .42<br>.29,<br>.54 | .30<br>.15,<br>.43 | .24<br>.09,<br>.37 | .12<br>-.04,<br>.26 | .44<br>.31,<br>.56 | .22<br>.07,<br>.36 | | .88<br>.84,<br>.91 | .73<br>.66,<br>.79 | .62<br>.53,<br>.69 | .74<br>.67,<br>.79 | .62<br>.53,<br>.70 | .36<br>.24,<br>.47 | .32<br>.20,<br>.44 |
| 15 | .08<br>-.07<br>.23 | .14<br>-.02<br>.29 | .02<br>-.13<br>.17 | .45<br>.32,<br>.57 | .15<br>-.01<br>.29 | .17<br>.02,<br>.32 | .30<br>.16,<br>.44 | .42<br>.28,<br>.53 | .23<br>.08,<br>.37 | .23<br>.08,<br>.37 | .10<br>-.05,<br>.25 | .40<br>.27,<br>.52 | .14<br>-.01,<br>.28 | .97<br>.95,<br>.97 | | .68<br>.60,<br>.75 | .58<br>.49,<br>.66 | .73<br>.66,<br>.79 | .61<br>.52,<br>.69 | .42<br>.31,<br>.53 | .38<br>.26,<br>.49 |
| 16 | .11<br>-.05<br>.26 | .14<br>-.02<br>.29 | .05<br>-.10<br>.21 | .28<br>.14,<br>.42 | .05<br>-.10<br>.21 | .12<br>-.04<br>.27 | .18<br>.03,<br>.33 | .29<br>.14,<br>.43 | .15<br>-.00,<br>.29 | .14<br>-.01,<br>.29 | .06<br>-.10,<br>.21 | .39<br>.26,<br>.51 | .08<br>-.07,<br>.23 | .77<br>.70,<br>.83 | .79<br>.73,<br>.84 | | .45<br>.33,<br>.55 | .59<br>.49,<br>.67 | .55<br>.45,<br>.63 | .37<br>.25,<br>.48 | .33<br>.20,<br>.44 |
| 17 | .14<br>-.01<br>.28 | .05<br>-.10<br>.20 | .03<br>-.12<br>.18 | .36<br>.23,<br>.49 | .07<br>-.08<br>.22 | .13<br>-.02<br>.28 | .20<br>.05,<br>.35 | .31<br>.16,<br>.44 | .09<br>-.06,<br>.24 | .08<br>-.06,<br>.23 | -.04<br>-.19,<br>.11 | .23<br>.08,<br>.36 | .20<br>.05,<br>.34 | .63<br>.53,<br>.71 | .64<br>.54,<br>.72 | .48<br>.35,<br>.58 | | .43<br>.31,<br>.53 | .31<br>.19,<br>.42 | .22<br>.09,<br>.34 | .18<br>.05,<br>.30 |
| 18 | .20<br>.05,<br>.35 | .15<br>-.00<br>.30 | -.03<br>-.18<br>.13 | .35<br>.21,<br>.48 | .18<br>.03,<br>.33 | .16<br>.01,<br>.31 | .33<br>.18,<br>.46 | .35<br>.20,<br>.48 | .16<br>.00,<br>.30 | .20<br>.05,<br>.34 | .12<br>-.03,<br>.27 | .33<br>.19,<br>.46 | .15<br>.00,<br>.30 | .74<br>.66,<br>.81 | .73<br>.65,<br>.80 | .59<br>.48,<br>.68 | .50<br>.38,<br>.60 | | .86<br>.82,<br>.89 | .36<br>.24,<br>.47 | .29<br>.17,<br>.41 |
| 19 | .19<br>.04,<br>.33 | .19<br>.04,<br>.34 | -.07<br>-.21<br>.09 | .30<br>.16,<br>.43 | .23<br>.08,<br>.37 | .22<br>.07,<br>.36 | .23<br>.08,<br>.37 | .35<br>.21,<br>.48 | .34<br>.20,<br>.46 | .19<br>.05,<br>.34 | .06<br>-.09,<br>.21 | .24<br>.10,<br>.38 | .14<br>-.01,<br>.29 | .69<br>.60,<br>.76 | .68<br>.58,<br>.75 | .49<br>.37,<br>.60 | .44<br>.31,<br>.55 | .76<br>.69,<br>.82 | | .35<br>.23,<br>.46 | .24<br>.11,<br>.36 |
| 20 | -.06<br>-.21<br>.09 | .03<br>-.13<br>.18 | -.00<br>-.16<br>.15 | .10<br>-.05<br>.25 | .09<br>-.06<br>.24 | .06<br>-.09<br>.21 | .01<br>-.14<br>.17 | .16<br>.01,<br>.31 | .09<br>-.06,<br>.24 | .03<br>-.12,<br>.18 | -.07<br>-.22,<br>.08 | .13<br>-.02,<br>.27 | .06<br>-.09,<br>.21 | .30<br>.16,<br>.44 | .34<br>.20,<br>.47 | .28<br>.14,<br>.42 | .16<br>.01,<br>.31 | .23<br>.08,<br>.37 | .38<br>.24,<br>.50 | | .44<br>.32,<br>.54 |
| 21 | -.07<br>-.22<br>.09 | .01<br>-.15<br>.16 | -.05<br>-.21<br>.10 | .16<br>.00,<br>.30 | .00<br>-.15<br>.16 | .02<br>-.13<br>.18 | .06<br>-.10<br>.21 | -.00<br>-.16,<br>.15 | -.09<br>-.24,<br>.06 | .22<br>.07,<br>.36 | -.06<br>-.21,<br>.09 | .08<br>-.07,<br>.23 | .06<br>-.09,<br>.21 | .40<br>.26,<br>.52 | .44<br>.31,<br>.55 | .41<br>.28,<br>.53 | .21<br>.07,<br>.35 | .32<br>.18,<br>.45 | .21<br>.06,<br>.35 | .32<br>.19,<br>.45 | |

1=total height, 2=sitting height, 3=knee height, 4=buttock-knee length, 5=foot length left, 6=foot length right, 7=head circumference, 8=head breadth, 9=head length 1, 10=head length 2, 11=face height, 12=shoulder breadth, 13=wrist breadth, 14=weight, 15=BMI, 16=arm circumference, 17=hip breadth, 18=waist circumference relaxed, 19=waist circumference sucking, 20=biceps skinfold, 21=triceps skinfold
